# An automated platform trial framework for A/B testing

**DOI:** 10.1016/j.conctc.2024.101388

**Published:** 2024-11-04

**Authors:** Wenru Zhou, Miranda Kroehl, Maxene Meier, Alexander Kaizer

**Affiliations:** a Department of Biostatistics and Informatics University of Colorado, United States of America; b Charter Communication, United States of America

**Keywords:** Interim monitoring, A/B testing, Error spending function, Stopping rule

## Abstract

This paper proposes a platform trial for conducting A/B tests with multiple arms and interim monitoring to investigate the impact of several factors on the expected sample size and probability of early stopping. We examined the performance of three stopping boundaries: O’Brien Fleming (OBF) stopping for either futility or difference (both), Pocock stopping for futility only, and fixed sample size design. We simulated twelve scenarios of different orders of arms based on various effect sizes, as well as considered 1 or 3 interim looks. The overall findings are summarizing in a flowchart to provide intuitive guidance for the design of the platform based on the simulation. We found that it is better to use OBF stopping for both if there is any effective variant and the trial is sufficiently powered to detect the expected effect size. If the study is underpowered to detect a difference, we recommend fixed sample size design to gather as much information as possible, however if the expected sample size is important to minimize, we recommend using Pocock boundaries with futility monitoring. Our results aimed at helping high-tech companies conduct their own studies without requiring extensive knowledge of clinical trial design and statistical methodology.

## 1 Introduction

A/B testing is a valuable tool for companies looking to evaluate the efficacy of their products. With a wide variety of new products constantly being developed, it can be challenging to determine which products are truly effective and worth investing in further. A/B testing provides a means to objectively measure the impact of product changes or innovations, allowing companies to make data-driven decisions and optimize their product offerings. A considerable number of papers have discussed the introduction, design, and application of A/B testing. For example, Kohavi et al. [1], Miller [2], and Koning et al. [3] gave a clear and thorough introduction of what A/B tests are and how to design an A/B test for large scale studies. Gupta et al. [4] introduced four core components of an A/B experimentation system, further providing guidance on important considerations in designing A/B studies. Interim monitoring approaches have been extensively applied and studied in traditional biomedical randomized controlled trials and provides the ability for an A/B test to stop prior to full enrollment for only futility, only an observed difference, or for both futility or a difference. Interim monitoring can save resources relative to a design with no interim stopping [5], [6].

However, much of the prior work in A/B testing has focused on a single A/B study. Previously, Zhou et al. [7] proposed an approach to incorporate formal interim monitoring to a single A/B testing study with a loss function based on sample size considerations to identify the “optimal” design. In many situations, there may be multiple variants a company wishes to evaluate for their performance. Further, a company may wish to “automate” this process of evaluating multiple variants so that more experiments can be conducted while minimizing the time between experiments or the need for additional human capital to continually implement A/B tests with the only change being the new variant being examined. Previous work by [8], [9] examine various modifications to A/B testing settings that could be automated, but their approaches do not focus on multiple arm comparisons.

Within biomedical literature, platform clinical trials have been proposed as a flexible design to examine multiple interventions. Berry et al. [10] examined the advantages of various platform trial designs relative to a traditional two-arm strategy, and found that platform trials identify beneficial treatments with fewer patients, fewer patient failures, less time, and with greater probability of success than a traditional two-arm strategy. In addition, the Adaptive Platform Trials Coalition broadly discussed the challenges and potential for novel application of these emerging designs in their 2019 work [11]. During the COVID-19 pandemic, platforms trials were widely used in comparing the efficacy of antiviral therapies. Kaizer et al. [12] used Trial of Early Antiviral Therapies during Non-hospitalized Outpatient Window (TREAT NOW) platform to evaluate potential agents of non-hospitalized patients with COVID-19, which is designed to accommodate testing multiple agents with the possibility to incorporate new agents in the future. A more general overview of the many versions of platform trials in the COVID-19 pandemic is discussed by Vanderbeek et al. [13]

In this paper, we propose an automatic sequential platform trial design for the evaluation of a sequence of variants evaluated through A/B tests while incorporating interim monitoring based on the choice of optimal interim monitoring strategy from our prior work [7]. We elucidate the performance of the automatic platform trial through a series of simulation studies that compare three different interim monitoring strategies before concluding with a brief discussion of the design properties and potential applications.

## 2 Background

### 2.1 Interim monitoring boundaries

We first provide a brief introduction of the interim monitoring boundaries and stopping rules implemented in this article. Further information can be found in the article by Zhou et al. [7].

First we introduce the error spending function $\alpha \left(t^{*}\right)$. It is a function of $t^{*}$, the information fraction observed at the time of the interim analysis. Practically, it is estimated by the fraction of participants enrolled at calendar time t divided by the maximum number of participants to be enrolled by the completion of the study. For example, when calendar time t=0, no participants are enrolled so the information fraction $t^{*}=\theta$ and the error spending function $\alpha \left(t^{*}=\theta \right)=0$. When the study ends, since all participants are enrolled, the information fraction $t^{*}=1$ and the error spending function $\alpha \left(t^{*}=1\right)=\alpha$.

In the context of error spending functions, Pocock boundaries can be approximated by the function $\alpha ln\left[1+\left(e-1\right)t^{*}\right]$, and for O’Brien-Fleming boundaries the approximate function is $2-2\theta \left(Z_{1-\alpha /2}/\sqrt{t^{*}}\right)$. We consider these two boundaries within this article because they are rooted in group sequential designs for randomized clinical trials and have different shapes that affect how aggressively they allow for early stopping. Additional details on these methods and their use in interim monitoring are further described in [14].

### 2.2 Stopping rules

There are many reasons to stop a study early, including safety and efficacy reasons. In studies with the goal of detecting some differences between groups, we consider three possible types of stopping rules for interim analyses:

1. Early stopping for a detectable difference only: In this scenario, during each interim analysis one assesses whether to stop the study based on evidence of a difference between the two variants in the A/B test. This is often framed as stopping for either superiority/benefit or inferiority/harm of one variant compared to the other. It is worth emphasizing that the difference here can imply efficacy or harm of the novel variant.

2. Early stopping for futility only: In this case, at each interim analysis, one evaluates whether to end the study due to evidence suggesting that continuing would not yield a detectable difference between the two variants even if the study were to reach its planned sample size.

3. Early stopping for both (either a detectable difference or futility): Here, during each interim analysis, one may decide to stop the study if a difference is detected between variants or if one determines that it is futile to continue.

## 3 Simulation study design

Our simulations consider an initial “standard” variant (labeled *S*) compared to five new variants (labeled *A*, *B*, *C*, *D*, and *E*) for a total of five sequential platform segments. The “winner” of each segment becomes the comparator for the subsequent segment. For example, in segment 1 variant *S* is compared to *A*. If *A* is declared to be better than *S*, segment 2 will compare *A* to *B*. If *A* is not determined to be significantly better, then segment 2 will compare *S* to *B*. The trial continues automatically in this manner until all five new variants have been evaluated and a final “winner” is declared for implementation.

A range of scenarios with a binary outcome are evaluated where lower response rates are considered better (e.g., reducing the call-in rate to a help center) and are summarized in Table 1. In addition to a null scenario with no effective new variant (Scenario 1), four scenarios consider a “diamond in the rough” where only one new variant is effective and the location of the effective variant changes to be at the 1st, 3rd, or 5th segment (Scenarios 1.1–1.4), and three scenarios consider the presence of two effective variants in the 2nd and 4th segment (Scenarios 2.2–2.3). In each scenario, the null response rate is considered to be 10%, with an alternative response rate of 9.5%. In addition, as a sensitivity analysis we explore the results if a stronger than expected response rate of 8% is observed. Finally, additional sensitivity analyses are presented with higher (15%) or lower (5%) null response rates and the properties if the effective variant is in the 3rd segment (Scenarios 3.1–3.4).

Interim monitoring was considered for designs with either 1- or 3-total interim analyses, corresponding to a total of at most 2- or 4-looks at the data. The choice of interim boundary (e.g., Pocock, O’Brien-Fleming, etc.) and stopping criteria (e.g., futility, difference, or both) was determined based on the optimal design selected from the loss function proposed by Zhou et al. [7]:

(1) $$L_{1}=w_{1}\frac{ESS_{null,boundary}}{SS_{fixed}}+w_{2}\frac{ESS_{alt,boundary}}{SS_{fixed}}+w_{3}\frac{MSS_{boundary}}{SS_{fixed}}$$

where $w_{1}+w_{2}+w_{3}=1$, and $SS_{fixed}$ is the sample size of a fixed sample size design without interim monitoring. This loss function is a linear combination of the weighted ratio of designs with interim monitoring relative to the fixed sample design without interim monitoring while considering the expected sample size under the null hypothesis ($ESS_{null}$), the expected sample size under the alternative hypothesis ($ESS_{alt}$), and the maximum sample size (MSS) if the study does not stop early. We consider three sets of weights with the following optimal stopping boundaries for both 2- and 4-looks of:

1. $w_{1}=w_{2}=w_{3}=\frac{1}{3}$: an equal, agnostic weighting of each component which identified the O’Brien-Fleming style boundary that stops for both difference or futility as optimal [15]. We denoted this as O’Brien Fleming with Early Stopping for Both.
2. $w_{1}=0.9$, $w_{2}=w_{3}=0.05$: weights that prioritize minimizing the sample size under a null hypothesis where the Pocock style boundary that stops only for futility is the optimal stopping boundary [16].
3. $w_{1}=w_{2}=0.05$, $w_{3}=0.9$: a priority for minimizing the maximum sample size if the study does not stop early where the optimal design is a fixed sample design that does not allow for any early stopping.

The maximum sample size needed for each design is selected to detect the difference of 10% versus 9.5% with 80% power and a 5% type I error rate for a two-sided hypothesis test. To more closely replicate the analysis approach used in many companies for both continuous and binary outcomes for large samples, we use a two-sample t-test [17]. In our motivating context, we will only declare a new variant “better” if it reduces the response, otherwise the conclusion is that the “control” variant is the winner to carry forward to the next segment of the platform trial. The maximum sample size is calculated based on the chosen interim monitoring design with boundaries identified using package “rpact” [18]. Summaries from the simulations include the expected sample size (ESS) per segment and for the overall platform trial across all five segments, the proportion of times each segment is considered the “winner”, and the proportion of simulations where each type of early stopping takes place. A total of 1000 simulations are conducted for each scenario using R v4.2.0 (Vienna, Austria) [19].

**Table 1:** Simulation settings for automated platform trial.

Design	Scenario	$\theta_{A}$	$\theta_{B}$	$\theta_{C}$	$\theta_{D}$	$\theta_{E}$
Total null ($\theta_{S}$ = 10%)	1	10.00%	10.00%	10.00%	10.00%	10.00%

Diamond in the rough ($\theta_{S}$ = 10%): only 1 A/B test is meaningful	1.1	**9.50%**	10.00%	10.00%	10.00%	10.00%
	1.2	10.00%	10.00%	**9.50%**	10.00%	10.00%
	1.3	10.00%	10.00%	10.00%	10.00%	**9.50%**
	1.4	10.00%	10.00%	**8.00%**	10.00%	10.00%

Multiple effective variants ($\theta_{S}$ = 10%)	2.1	10.00%	**9.50%**	10.00%	**9.50%**	10.00%
	2.2	10.00%	**8.00%**	10.00%	**9.50%**	10.00%
	2.3	10.00%	**9.50%**	10.00%	**8.00%**	10.00%

Sensitivity analyses ($\theta_{S}$ = 15% or 5%): Assuming the sample sizes from above	3.1	15.00%	15.00%	15.00%	15.00%	15.00%
	3.2	15.00%	15.00%	**14.25%**	15.00%	15.00%
	3.3	5.00%	5.00%	5.00%	5.00%	5.00%
	3.4	5.00%	5.00%	**4.75%**	5.00%	5.00%

## 4 Results

In this section we describe the results from the simulation studies based on the various operating characteristics and sensitivity analyses. A representative subset of simulation scenarios are presented, with complete results included in the Supplementary Materials.

### 4.1 Overall platform trial performance

The overall expected sample sizes for each scenario and each stopping boundary are summarized in Table 2, as well as the proportions for each arm being selected as the winner at the end of the trial. The column names indicate each arm, expected sample size (ESS), standard deviation (SD), scenario, and stopping boundary.

In the null scenario, the standard arm was selected as the winner in 88.5% of all simulated trials, this reflects an estimate of ${\left(1-0.025\right)}^{5}$ since any null segment would be falsely declared a winner 2.5% of the time in our two-sided hypothesis test (i.e., one-tail represents the new variant has “won” whereas the other tail represents the current standard variant has “won”). The bold numbers in Table 2 indicate segments where an effective therapy is present, which can be thought of as the “power” to detect the effect in the sequential platform trial design. For example, Scenario 1.1, 1.2, 1.3, and 2.1 should have power around 0.8, but the actual power is slightly lower than that due to Monte Carlo error.

The overall ESS is generally lower when the number of total looks increases for designs with interim monitoring. This is because more looks at the data provide additional opportunities to stop a segment early for futility or detecting a difference. One exception can be seen in Scenario 1.1 (i.e., effective variant in 2nd segment) with Pocock monitoring for futility, where the ESS increases with additional looks. This occurs because the effective variant occurring early in the platform trial leads to most subsequent platform segments to compare different response rates, resulting in a setting for a two-sided hypothesis test where stopping for a difference (i.e., efficacy, or harm, not just futility) would be needed.

**Table 2:** Proportion of simulations where each variant is the overall winner at the end of the platform trial for the 2- or 4-total analysis scenarios with the overall expected sample size (ESS) and its standard deviation (SD) across all five platform segments, bolded cells represent effective variant(s) in a scenario.

Scenario & Boundary	S	A	B	C	D	E	ESS	SD
*2-total looks*								

1. Fixed Design	0.885	0.021	0.032	0.020	0.019	0.023	276 260	0
1. OBF for both	0.883	0.023	0.029	0.019	0.020	0.026	241 106	28 169
1. Pocock for futility	0.885	0.025	0.025	0.020	0.022	0.023	206 551	30 978

1.1. Fixed Design	0.200	**0.788**	0.005	0.003	0.004	0	276 260	0
1.1. OBF for both	0.189	**0.796**	0.003	0.001	0.005	0.006	248 703	26 424
1.1. Pocock for futility	0.202	**0.786**	0.003	0	0.006	0.003	269 934	42 490

1.2. Fixed Design	0.190	0.007	0.006	**0.785**	0.003	0.009	276 260	0
1.2. OBF for both	0.198	0.007	0.007	**0.775**	0.004	0.009	246 649	26 932
1.2. Pocock for futility	0.199	0.005	0.005	**0.782**	0.003	0.006	247 490	32 652

2.1. Fixed Design	0.040	0	**0.788**	0.004	**0.167**	0.001	276 260	0
2.1. OBF for both	0.040	0	**0.776**	0.002	**0.181**	0.001	248 281	27 337
2.1. Pocock for futility	0.039	0.002	**0.777**	0.002	**0.180**	0	252 532	29 077

*4-total looks*								

1. Fixed Design	0.885	0.021	0.032	0.020	0.019	0.023	276 260	0
1. OBF for both	0.879	0.025	0.032	0.018	0.021	0.025	210 409	21 903
1. Pocock for futility	0.892	0.029	0.021	0.013	0.023	0.022	188 003	33 986

1.1. Fixed Design	0.200	**0.788**	0.005	0.003	0.004	0	276 260	0
1.1. OBF for both	0.195	**0.791**	0.005	0.001	0.005	0.003	222 341	24 285
1.1. Pocock for futility	0.175	**0.810**	0.003	0.003	0.005	0.004	279 716	54 751

1.2. Fixed Design	0.190	0.007	0.006	**0.785**	0.003	0.009	276 260	0
1.2. OBF for both	0.199	0.006	0.008	**0.779**	0.002	0.006	218 194	22 763
1.2. Pocock for futility	0.182	0.004	0.004	**0.803**	0.002	0.005	246 466	41 206

2.1. Fixed Design	0.040	0	**0.788**	0.004	**0.167**	0.001	276 260	0
2.1. OBF for both	0.042	0.001	**0.778**	0.001	**0.178**	0	218 905	23 193
2.1. Pocock for futility	0.037	0	**0.797**	0.003	**0.162**	0.001	251 867	34 920

### 4.2 Segment-specific expected sample size

**Fig. 1:**
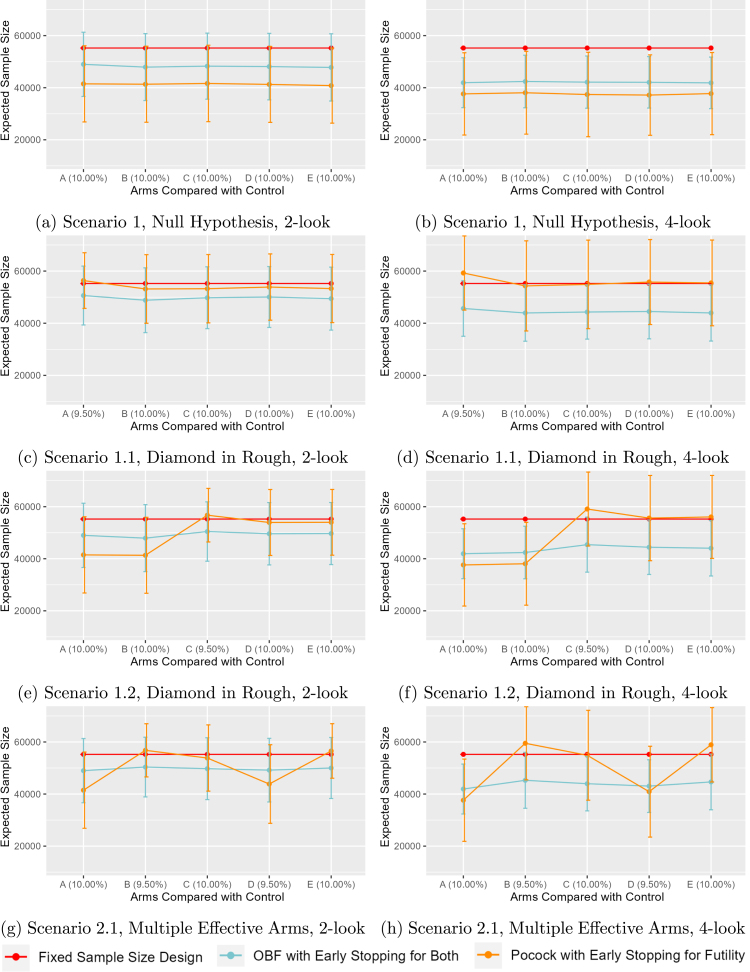
Expected Sample Size versus Comparison, 2- vs 4-total Looks.

In Scenario 1, which has no effective variant in any segment, it can be seen in Fig. 1, Fig. 1 that the ESS is minimized when using the Pocock design that only allows for futility with either 2- or 4-total looks, respectively. However, the improvement in ESS is less with 4-total looks, suggesting that the O’Brien-Fleming monitoring with early stopping for futility or some difference becomes more similar to Pocock across all null arms as the number of planned interim looks increases. In both scenarios, designs with interim monitoring outperform the fixed sample design with lower ESS.

The “Diamond in the Rough” scenarios with only one effective variant demonstrate the benefit of using the O’Brien-Fleming monitoring for both futility and differences. When variant *A* in the first segment is the effective variant (Scenario 1.1), the ESS for 2-total looks in Fig. 1(c) shows that the Pocock ESS is similar to the fixed sample design across all segments, but with large variability, whereas the O’Brien-Fleming is constantly the lowest. This pattern is accentuated for 4-total looks in Fig. 1(d). When variant *C* in the third segment is the effective variant (Scenario 1.2), Fig. 1, Fig. 1 show that the first two segments with 10% null effects have identical performance to the null Scenario 1 with Pocock having the lowest ESS, but a similar pattern to Scenario 1.1 in the third or later segments with O’Brien-Fleming having the lowest ESS.

For remaining Scenarios (1.3, 1.4, 2.2, and 2.3) results are presented in the Supplementary Materials. Similar trends are observed with respect to the ordering of the effective segments. When a variant has an alternative response rate of 8%, the effect on the ESS is further exaggerated with all O’Brien-Fleming stopping boundary results having the same conclusion of terminating early for difference for the 8% effect and terminating early for futility for the subsequent 10% null effect in later variants. In contrast, the Pocock design never stops early because there is a difference (e.g., 8% versus 10%).

The sensitivity scenarios where the null response rate is actually 15% (Fig. 3, Fig. 3) show similar patterns to the expected design with a 10% null response when an effective variant is present in the third segment in that Pocock has the lowest ESS in the first two null segments, but then has the highest ESS in segments three, four, or five. In contrast, the O’Brien-Fleming design that stops for both futility or a difference has fairly stable ESS across all segments, with the lowest ESS in the third segment onward. The sensitivity analysis with the 5% null (Fig. 3, Fig. 3) has similar results when the null is present, but it can be seen that the ESS is similar to the O’Brien-Fleming in the third segment with 2-total looks and similar in the fourth and fifth with 4-total looks. This is likely a consequence of the simulation being underpowered relative to the expected difference of 0.5% (i.e., 10% versus 9.5%) in the main scenarios.

### 4.3 Proportion of trials stopping at interim looks

**Fig. 2:**
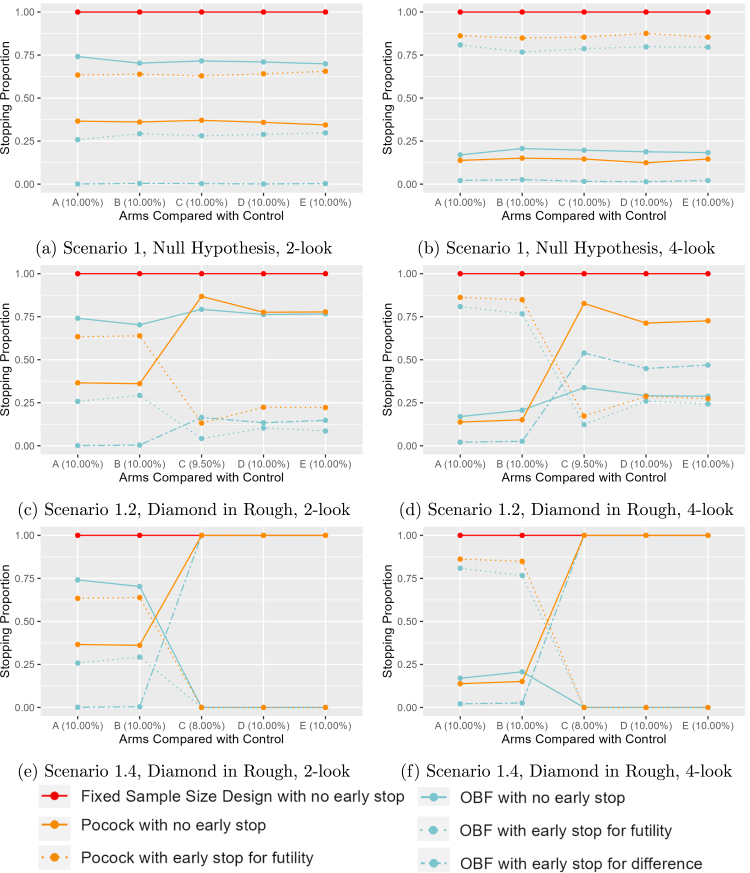
Proportion of simulations with given stopping criteria for 2- and 4-total looks. Red represents Fixed Sample Size Design; Cyan represents O’Brien Fleming with Early Stopping for Both; Orange represents Pocock with Early Stopping for Futility Solid line represents No Early Stop; Dotted line represents Early Stop for Futility; Dot-Dashed line represents Early Stop for Difference.

We present a smaller subset of results in Figs. 2(a)–2(f) to demonstrate the change in proportion of simulated trials that stop early for futility, for difference, or never stop early with respect to 2- versus 4-total looks and varying the location of an effective variant. The y-axis is the proportion for each reason the trial could stop (i.e., early stopping for futility, early stopping for a difference, or no early stopping). For the fixed sample size design with no early stopping, the only way to “stop” the trial is to complete the full enrollment. For the boundary called O’Brien Flemming with early stop for both, there are three possible ways to terminate the trial: the trial comes to the end without early termination, the trial is terminated early since a difference is observed, or the trial is terminated early due to futility.

In Scenario 1, which has no effective variant in any segment, it can be seen in Fig. 2, Fig. 2 that the O’Brien-Fleming design rarely stops for a difference with its plotted line near 0% for all segments. However, the proportion of OBF with the choice of no early stop is approximately 75% in 2- interim looks, which is much higher than the proportion below 25% in 4-interim looks. This indications that with 2-interim looks it is more likely to continue to its maximum sample size in approximately 75% of simulations, but this pattern flips for 4-total looks with approximately 75% of simulations terminating early due to futility. This is due to the conservative nature of the O’Brien-Fleming design, but with an increasing number of interim looks there is a greater chance to determine the given null A/B test is futile. The Pocock boundary stops for futility approximately 63% and 88% with 2- and 4-total looks, respectively.

The limitations of only stopping for futility become readily apparent in any scenario with at least one effective therapy. For example, in Scenario 1.2 presented in Fig. 2, Fig. 2, with a single effective variant with a response of 9.5% in the third segment, it can be seen that the Pocock design increases to full enrollment in over 70% of comparisons at or after the segment with the effective variant. This implies that it does terminate for futility in some trials, which may be due to the effective variant not being identified as better (i.e., a type II error) with future segments essentially being a null comparison of 10% versus 10% or due to a false conclusion of futility. In contrast, the O’Brien-Fleming design sees an increase in its probability of stopping for a difference of approximately 13% with 2-total looks and 50% with 4-total looks since the line of OBF with early stop for difference increased from 0% to approximately 13% in 2-interim looks and from 0% to approximately 50% in 4-interim looks.

In Scenario 1.4, the effective therapy is larger than expected at 8% in the third segment, and all designs stop for the same reason for the third segment onward (Fig. 2, Fig. 2). The fixed sample and Pocock designs never terminate early, since there is a strong effect that is always detected in the third segment. In contrast, the O’Brien-Fleming design always terminates early for a detected difference.

The Supplementary Materials includes figures summarizing the proportion of trials stopping for a given reason for the scenarios not presented here. Briefly, they each present similar effects to the three representative scenarios. The O’Brien-Fleming design is less likely to stop early for either futility or some difference with only 2-looks compared to 4-looks. The Pocock design is less likely to terminate early on or after the segment with an effective variant.

### 4.4 Position of effective therapies

The position of the effective arm may affect the trial’s operating characteristics when only one variant is effective (Fig. 1(c), 1(d), 1(e), 1(f) of Scenario 1.1 and 1.2). For O’Brien Fleming stopping for both futility or a difference, the position of the effective variant does not have a large influence on ESS. However, for Pocock with only futility stopping, a later effective variant has much lower ESS than an earlier effective variant. This can be explained as: If we compare the effective arm and standard arm at the beginning, then for all of the comparisons, we are testing some difference, but the design only allows early stopping for futility and therefore the ESS will be higher than if no interim analyses were applied.

The position of multiple effective arm can also affect the trial’s operating characteristics when two variants are effective (Fig. 1(g), 1(h) of Scenario 2.1). For OBF stopping for futility or some difference, earlier positioning of the most effective arm reduces ESS more than later positions in the platform sequence. This is because once the most effective arm is chosen, we have higher chance to stop for a difference comparing to a less effective arm. Similar to the one effective variant scenarios, only Pocock stopping for futility indicates that the most effective arm should actually be as late as possible, since the interim monitoring strategy does not stop for a difference.

**Fig. 3:**
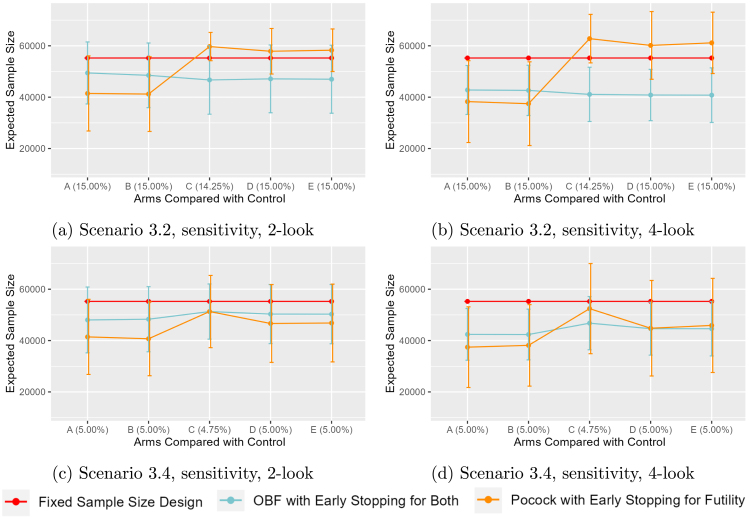
Sensitivity Analysis Collection. Expected Sample Size versus Comparison, 2- vs 4-total Looks. Red represents Fixed Sample Size Design; Cyan represents O’Brien Fleming with Early Stopping for Both; Orange represents Pocock with Early Stopping for Futility Solid line represents No Early Stop; Dotted line represents Early Stop for Futility; Dot-Dashed line represents Early Stop for Difference.

## 5 Discussion

Unlike human clinical trials where each trial is carefully designed, high-tech companies may run thousands of A/B tests simultaneously. It is practically impossible to custom design every individual A/B test. Therefore an automatic platform is needed, which can be used for all A/B tests in a given development pipeline. Our proposed automated sequential platform trial design is flexible and can accommodate various outcomes and forms of interim monitoring. Although this article uses binary outcomes to illustrate the concept, the platform could also handle continuous or count outcomes. This is because interim monitoring methods like Pocock and O’Brien-Fleming style stopping boundaries have been applied to all forms of outcomes in traditional clinical trials. For illustration, we presented three strategies based on the “optimal” monitoring plan as determined by a loss function proposed in [7]: a fixed sample design with no interim monitoring, a Pocock-style design with early stopping for only futility, and an O’Brien-Fleming style design that allows early stopping for both futility or a difference. When no differences exist and prior to any effective variant, Pocock boundaries tended to result in greatest improvement in the expected sample size. However, the O’Brien-Fleming design resulted in a lower average sample size after an effective variant was present in the platform trial pipeline.

**Fig. 4:**
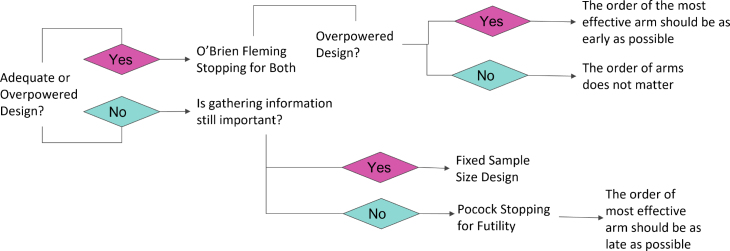
Flow chart for identification of optimal sequential platform trial design based on the context of the given A/B testing environment.

To further summarize our results and provide practical guidance of analytic teams in industry that may be conducting multiple A/B tests, we provide a summary flow chart in Fig. 4. This flow chart provides a series of prompts asking about the potential power and reasons for a study. If a study is expected to be underpowered, it is ideal to allow early termination for futility to most rapidly move through the platform pipeline unless gathering additional safety information or descriptive summaries are useful, in which case a fixed design would be optimal to ensure this information is collected. In contrast, if you expect to be adequately or over-powered, a design that facilitates stopping for both futility or a difference is optimal, but the order of the arms with an effective variant may not be as important in an adequately powered study based on our O’Brien-Fleming results. In contrast, an overpowered study benefits from placing the effective variant as early as possible to facilitate rapid early termination for futility of all subsequent segments of the platform. Although we cannot know exactly which variant is the most efficient in advance, one can still try to propose the order of variants in the pipeline to reflect any prior information or more effective hypothesized performance. For O’Brien Fleming with early stopping for both, the most efficacious arm should be placed as early as possible regardless of the number of effective arms. Since the O’Brien Fleming boundary is more conservative at early interim analysis and is more liberal at late interim analysis within any given platform segment, we also recommend that the number of interim analysis is greater than two. However, for Pocock boundary with early stopping for futility, the most effective arm should be as late as possible.

This study has some potential limitations worth discussing. First, many platform trial designs consider some form of shared control arm for their comparisons wherein there may be concerns with regards to corrections for multiple comparisons and stricter control of the family-wise type I error rate [20], [21], [22]. However, we highlight that in our sequential platform trial design we prospectively enroll a new control group and that our improved efficiencies are found in the reduction of delayed time often required between separate standlone trials. In practice, if one wishes to still control the family-wise error rate there are multiple approaches that can be easily applied such as Bonferroni, Simes, step up, step down, or fallback methods; but we believe these incorporate an unnecessary penalty in our sequential platform trial design compared to a series of standalone two-arm trials that would not traditionally have such corrections. Another potential limitation is that we did not consider a design where all variants are evaluated simultaneously, such as a multi-arm, multi-stage design with a common control. This design may be more efficient if there is adequate resources (e.g., sample size) to conduct the study, but may also be limited in that it does not traditionally facilitate all pairwise comparisons of arms to identify the overall “winner” [23].

Currently, this study does not address practical issues such as the correlation in randomization. In A/B tests, the simplest approach is to assume user independence when randomizing. However, users may be related or belong to the same unit of analysis (e.g., family, company, or organization), leading to potentially important dependencies. Future work will extend the proposed platform trial design to accommodate these considerations. This article focuses on the even sample sizes among all interim analyses. In the future, it is worth considering the uneven sample sizes in each interim analyses. Finally, the proposed design considered a sequential platform, however the potential for increased efficiency with multi-arm platform trials with a shared control arm or incorporating adaptive elements related to the results of earlier segments present future directions of research in A/B testing.

In summary, we propose an automated sequential platform trial design to seamlessly conduct a series of A/B tests. The platform trial design is flexible and could accommodate any type of outcome or interim monitoring strategy. However, we recommend using an O’Brien-Fleming boundary that facilitates stopping for either futility or a difference with at least 3-interim looks to increase the potential efficiency of the overall trial across multiple sequential segments.

## CRediT authorship contribution statement

**Wenru Zhou:** Writing – review & editing, Writing – original draft, Visualization, Validation, Software, Project administration, Methodology, Investigation, Formal analysis, Data curation, Conceptualization. **Miranda Kroehl:** Resources, Investigation, Conceptualization. **Maxene Meier:** Resources, Investigation, Conceptualization. **Alexander Kaizer:** Writing – review & editing, Validation, Supervision, Resources, Project administration, Methodology, Investigation, Funding acquisition, Conceptualization.

## Declaration of competing interest

The authors declare that they have no known competing financial interests or personal relationships that could have appeared to influence the work reported in this paper.

## Data Availability

Data will be made available on request.

## Appendix A Supplementary data

MMC S1 Detailed simulated results supporting the findings on the automated platform trial framework.
