# Supplementary material for: An automated platform trial framework for A/B testing
Source: Contemp Clin Trials Commun. 2024 Nov 4;42:101388. doi: 10.1016/j.conctc.2024.101388 (PMC11602995; doi:10.1016/j.conctc.2024.101388)
Supplement: MMC S1 — Detailed simulated results supporting the findings on the automated platform trial framework. [file mmc1.pdf]

Supplementary Materials for An Automated Platform Trial Framework for A/B Testing

Wenru Zhou[1], Miranda Kroehl[2], Maxene Meier[2], and Alexander Kaizer[1]

[1]Department of Biostatistics and Informatics University of Colorado

[2]Charter Communication

October 23, 2024

0.1 Percent of Arm Being Selected as Optimal

Table 1 - Table 2 illustrated the expected sample size and standard deviation for each different scenarios and boundaries for 2- and 4-total analysis. In addition, they summarized the percent of each arm being selected as the optimal choice.

0.2 Track of Comparisons between Five Arms

Table 3 - Table 14 summarized the track of each comparison between five arms. Take Table 3, look 2, scenario 1, and fixed sample size design for example: At the beginning stage of the platform, the outcomes from S (control) and A (treatment) are compared. Then S wins

in 97.7% of the simulated trials, while A wins in 2.3% of the simulated trials. For the next round of comparison, where the outcome from winner is compared with the outcome from B, S wins 94.3% of the simulated trials, A wins 2.2% of the trials, and B wins 3.4% of the trials. For the next round of comparison, where the outcome from winner is compared with tHe outcome from C, S wins 92.5% of the simulated trials, A wins 2.1% simulated trials, B wins 3.4% simulated trials, and C wins 2% simulated trials. For the next round of comparison, where the outcome from winner is compared with the outcome from D, the percentage of being selected as winner for S, A, B, C, D are 90.6%, 2.1%, 3.4%, 2%, and 1.9%. And after comparing with E, the final percentages of winners from S to E are: 88.5%, 2.1%, 3.2%, 2%, 1.9%, 2.3%.

0.3 Plots of Expected Sample Size and Early Stopping Percentages

Besides the plots in main paper document, all plots of Expected Sample Size versus Comparisons between Arms and Early Stopping Percentages versus Comparisons between Arms are at the end of this supplementary material.

Table 1: Percent of Arm Being Selected as Optimal for the 2-total analysis

**S**	**A**	**B**	**C**	**D**	**E**	**ESS**	**SD**	**Scenario&Boundary**
0.885	0.021	0.032	0.02	0.019	0.023	276260	0	1. Fixed Design
0.883	0.023	0.029	0.019	0.02	0.026	241105.953	28168.5133	1. OBF for both
0.885	0.025	0.025	0.02	0.022	0.023	206551.37	30977.9871	1. Pocock for futility
0.2	**0.788**	0.005	0.003	0.004	0	276260	0	1.1. Fixed Design
0.189	**0.796**	0.003	0.001	0.005	0.006	248702.94	26424.24	1.1. OBF for both
0.202	**0.786**	0.003	0	0.006	0.003	269933.56	42489.94	1.1. Pocock for futility
0.19	0.007	0.006	**0.785**	0.003	0.009	276260	0	1.2. Fixed Design
0.198	0.007	0.007	**0.775**	0.004	0.009	246648.94	26931.68	1.2. OBF for both
0.199	0.005	0.005	**0.782**	0.003	0.006	247490.13	32652.41	1.2. Pocock for futility
0.189	0.005	0.007	0	0.003	**0.796**	276260	0	1.3. Fixed Design
0.185	0.007	0.007	0	0.005	**0.796**	244172.89	28042.71	1.3. OBF for both
0.206	0.006	0.007	0.002	0.004	**0.775**	222404.51	30607.28	1.3. Pocock for futility
0	0	0	**1**	0	0	276260	0	1.4. Fixed Design
0	0	0	**1**	0	0	181314.83	17644.78	1.4. OBF for both
0	0	0	**1**	0	0	265043.99	19982.07	1.4. Pocock for futility
0.04	0	**0.788**	0.004	**0.167**	0.001	276260	0	2.1. Fixed Design
0.04	0	**0.776**	0.002	**0.181**	0.001	248280.89	27337.35	2.1. OBF for both
0.039	0.002	**0.777**	0.002	**0.18**	0	252531.55	29077.46	2.1. Pocock for futility
0	0	**1**	0	0	0	276260	0	2.2. Fixed Design
0	0	**1**	0	0	0	161534.52	12332.58	2.2. OBF for both
0	0	**1**	0	0	0	284450.42	14636.84	2.2. Pocock for futility
0	0	0	0	**1**	0	276260	0	2.3. Fixed Design
0	0	0	0	**1**	0	205371.96	20907.73	2.3. OBF for both
0	0	0	0	**1**	0	273608.33	24589.85	2.3. Pocock for futility
0.88	0.014	0.029	0.025	0.027	0.025	276260	0	3.1. Fixed Design
0.885	0.016	0.029	0.023	0.023	0.024	241809.38	28752.22	3.1. OBF for both
0.875	0.021	0.021	0.031	0.027	0.025	205913.6	31454.18	3.1. Pocock for futility
0.053	0	0.001	**0.943**	0.001	0.002	276260	0	3.2. Fixed Design
0.061	0.002	0.001	**0.934**	0	0.002	238770.58	28622.93	3.2. OBF for both
0.057	0	0.001	**0.942**	0	0	258544.81	26832.57	3.2. Pocock for futility
0.893	0.018	0.031	0.013	0.022	0.023	276260	0	3.3. Fixed Design
0.889	0.011	0.035	0.019	0.02	0.026	243385.05	28144.04	3.3. OBF for both
0.903	0.014	0.023	0.011	0.019	0.03	205579.53	32007.58	3.3. Pocock for futility
0.464	0.009	0.018	**0.482**	0.013	0.014	276260	0	3.4. Fixed Design
0.463	0.006	0.019	**0.488**	0.011	0.013	248224.61	26888.57	3.4. OBF for both
0.462	0.01	0.013	**0.491**	0.007	0.017	226899.27	36476.91	3.4. Pocock for futility

Table 2: Percent of Arm Being Selected as Optimal for the 4-total analysis

**S**	**A**	**B**	**C**	**D**	**E**	**ESS**	**SD**	**Scenario&Boundary**
0.885	0.021	0.032	0.02	0.019	0.023	276260	0	1. Fixed Design
0.879	0.025	0.032	0.018	0.021	0.025	210408.543	21902.583	1. OBF for both
0.892	0.029	0.021	0.013	0.023	0.022	188002.719	33986.4159	1. Pocock for futility
0.2	**0.788**	0.005	0.003	0.004	0	276260	0	1.1. Fixed Design
0.195	**0.791**	0.005	0.001	0.005	0.003	222340.645	24284.74	1.1. OBF for both
0.175	**0.81**	0.003	0.003	0.005	0.004	279716.065	54750.7283	1.1. Pocock for futility
0.19	0.007	0.006	**0.785**	0.003	0.009	276260	0	1.2. Fixed Design
0.199	0.006	0.008	**0.779**	0.002	0.006	218194.157	22763.4157	1.2. OBF for both
0.182	0.004	0.004	**0.803**	0.002	0.005	246466.317	41206.0019	1.2. Pocock for futility
0.189	0.005	0.007	0	0.003	**0.796**	276260	0	1.3. Fixed Design
0.189	0.008	0.008	0.002	0.004	**0.789**	213989.639	22504.6124	1.3. OBF for both
0.186	0.003	0.007	0.001	0.004	**0.799**	209637.829	33921.9787	1.3. Pocock for futility
0	0	0	**1**	0	0	276260	0	1.4. Fixed Design
0	0	0	**1**	0	0	129780.172	14711.8259	1.4. OBF for both
0	0	0	**1**	0	0	270899.351	22032.1813	1.4. Pocock for futility
0.04	0	**0.788**	0.004	**0.167**	0.001	276260	0	2.1. Fixed Design
0.042	0.001	**0.778**	0.001	**0.178**	0	218904.574	23192.8692	2.1. OBF for both
0.037	0	**0.797**	0.003	**0.162**	0.001	251866.961	34920.3629	2.1. Pocock for futility
0	0	**1**	0	0	0	276260	0	2.2. Fixed Design
0	0	**1**	0	0	0	107886.889	13330.7165	2.2. OBF for both
0	0	**1**	0	0	0	297902.571	15804.7528	2.2. Pocock for futility
0	0	0	0	**1**	0	276260	0	2.3. Fixed Design
0	0	0	0	**1**	0	166172.254	19668.5461	2.3. OBF for both
0	0	0	0	**1**	0	282172.382	31768.9891	2.3. Pocock for futility
0.88	0.014	0.029	0.025	0.027	0.025	276260	0	3.1. Fixed Design
0.881	0.016	0.029	0.03	0.025	0.019	211959.924	21298.5302	3.1. OBF for both
0.864	0.029	0.032	0.02	0.028	0.027	188718.467	34897.9337	3.1. Pocock for futility
0.053	0	0.001	**0.943**	0.001	0.002	276260	0	3.2. Fixed Design
0.058	0.001	0.002	**0.938**	0	0.001	208161.213	22887.7412	3.2. OBF for both
0.062	0.002	0.003	**0.932**	0	0.001	259837.791	34101.2569	3.2. Pocock for futility
0.893	0.018	0.031	0.013	0.022	0.023	276260	0	3.3. Fixed Design
0.879	0.019	0.033	0.019	0.022	0.028	211524.928	21739.7369	3.3. OBF for both
0.883	0.014	0.032	0.018	0.027	0.026	189336.613	35060.2886	3.3. Pocock for futility
0.464	0.009	0.018	**0.482**	0.013	0.014	276260	0	3.4. Fixed Design
0.451	0.013	0.018	**0.494**	0.009	0.015	220774.876	22804.3367	3.4. OBF for both
0.437	0.006	0.024	**0.505**	0.01	0.018	218682.281	43521.1688	3.4. Pocock for futility

Table 3: Track of Comparisons Between Arms, Scenario 1

Look	Boundary	Comparison	S	A	B	C	D	E
2	1. Fixed Design	A	1	0	0	0	0	0
2	1. Fixed Design	B	0.977	0.023	0	0	0	0
2	1. Fixed Design	C	0.943	0.022	0.035	0	0	0
2	1. Fixed Design	D	0.925	0.021	0.034	0.02	0	0
2	1. Fixed Design	E	0.906	0.021	0.034	0.02	0.019	0
2	1. Fixed Design	Winner	0.885	0.021	0.032	0.02	0.019	0.023
2	1. OBF for both	A	1	0	0	0	0	0
2	1. OBF for both	B	0.975	0.025	0	0	0	0
2	1. OBF for both	C	0.946	0.024	0.03	0	0	0
2	1. OBF for both	D	0.929	0.023	0.029	0.019	0	0
2	1. OBF for both	E	0.909	0.023	0.029	0.019	0.02	0
2	1. OBF for both	Winner	0.883	0.023	0.029	0.019	0.02	0.026
2	1. Pocock for futility	A	1	0	0	0	0	0
2	1. Pocock for futility	B	0.972	0.028	0	0	0	0
2	1. Pocock for futility	C	0.947	0.026	0.027	0	0	0
2	1. Pocock for futility	D	0.927	0.026	0.027	0.02	0	0
2	1. Pocock for futility	E	0.907	0.026	0.025	0.02	0.022	0
2	1. Pocock for futility	Winner	0.885	0.025	0.025	0.02	0.022	0.023
4	1. Fixed Design	A	1	0	0	0	0	0
4	1. Fixed Design	B	0.977	0.023	0	0	0	0
4	1. Fixed Design	C	0.943	0.022	0.035	0	0	0
4	1. Fixed Design	D	0.925	0.021	0.034	0.02	0	0
4	1. Fixed Design	E	0.906	0.021	0.034	0.02	0.019	0
4	1. Fixed Design	Winner	0.885	0.021	0.032	0.02	0.019	0.023
4	1. OBF for both	A	1	0	0	0	0	0
4	1. OBF for both	B	0.971	0.029	0	0	0	0
4	1. OBF for both	C	0.939	0.026	0.035	0	0	0
4	1. OBF for both	D	0.922	0.026	0.034	0.018	0	0
4	1. OBF for both	E	0.902	0.025	0.033	0.018	0.022	0
4	1. OBF for both	Winner	0.879	0.025	0.032	0.018	0.021	0.025
4	1. Pocock for futility	A	1	0	0	0	0	0
4	1. Pocock for futility	B	0.97	0.03	0	0	0	0
4	1. Pocock for futility	C	0.948	0.03	0.022	0	0	0
4	1. Pocock for futility	D	0.934	0.03	0.022	0.014	0	0
4	1. Pocock for futility	E	0.913	0.029	0.021	0.014	0.023	0
4	1. Pocock for futility	Winner	0.892	0.029	0.021	0.013	0.023	0.022

Table 4: Track of Comparisons Between Arms, Scenario 1.1

Look	Boundary	Comparison	S	A	B	C	D	E
2	1.1. Fixed Design	A	1	0	0	0	0	0
2	1.1. Fixed Design	B	0.212	0.788	0	0	0	0
2	1.1. Fixed Design	C	0.207	0.788	0.005	0	0	0
2	1.1. Fixed Design	D	0.204	0.788	0.005	0.003	0	0
2	1.1. Fixed Design	E	0.2	0.788	0.005	0.003	0.004	0
2	1.1. Fixed Design	Winner	0.2	0.788	0.005	0.003	0.004	0
2	1.1. OBF for both	A	1	0	0	0	0	0
2	1.1. OBF for both	B	0.204	0.796	0	0	0	0
2	1.1. OBF for both	C	0.201	0.796	0.003	0	0	0
2	1.1. OBF for both	D	0.2	0.796	0.003	0.001	0	0
2	1.1. OBF for both	E	0.195	0.796	0.003	0.001	0.005	0
2	1.1. OBF for both	Winner	0.189	0.796	0.003	0.001	0.005	0.006
2	1.1. Pocock for futility	A	1	0	0	0	0	0
2	1.1. Pocock for futility	B	0.214	0.786	0	0	0	0
2	1.1. Pocock for futility	C	0.21	0.786	0.004	0	0	0
2	1.1. Pocock for futility	D	0.21	0.786	0.004	0	0	0
2	1.1. Pocock for futility	E	0.205	0.786	0.003	0	0.006	0
2	1.1. Pocock for futility	Winner	0.202	0.786	0.003	0	0.006	0.003
4	1.1. Fixed Design	A	1	0	0	0	0	0
4	1.1. Fixed Design	B	0.212	0.788	0	0	0	0
4	1.1. Fixed Design	C	0.207	0.788	0.005	0	0	0
4	1.1. Fixed Design	D	0.204	0.788	0.005	0.003	0	0
4	1.1. Fixed Design	E	0.2	0.788	0.005	0.003	0.004	0
4	1.1. Fixed Design	Winner	0.2	0.788	0.005	0.003	0.004	0
4	1.1. OBF for both	A	1	0	0	0	0	0
4	1.1. OBF for both	B	0.209	0.791	0	0	0	0
4	1.1. OBF for both	C	0.203	0.791	0.006	0	0	0
4	1.1. OBF for both	D	0.202	0.791	0.006	0.001	0	0
4	1.1. OBF for both	E	0.198	0.791	0.005	0.001	0.005	0
4	1.1. OBF for both	Winner	0.195	0.791	0.005	0.001	0.005	0.003
4	1.1. Pocock for futility	A	1	0	0	0	0	0
4	1.1. Pocock for futility	B	0.19	0.81	0	0	0	0
4	1.1. Pocock for futility	C	0.187	0.81	0.003	0	0	0
4	1.1. Pocock for futility	D	0.184	0.81	0.003	0.003	0	0
4	1.1. Pocock for futility	E	0.179	0.81	0.003	0.003	0.005	0
4	1.1. Pocock for futility	Winner	0.175	0.81	0.003	0.003	0.005	0.004

Table 5: Track of Comparisons Between Arms, Scenario 1.2

Look	Boundary	Comparison	S	A	B	C	D	E
2	1.2. Fixed Design	A	1	0	0	0	0	0
2	1.2. Fixed Design	B	0.977	0.023	0	0	0	0
2	1.2. Fixed Design	C	0.943	0.022	0.035	0	0	0
2	1.2. Fixed Design	D	0.2	0.007	0.008	0.785	0	0
2	1.2. Fixed Design	E	0.197	0.007	0.008	0.785	0.003	0
2	1.2. Fixed Design	Winner	0.19	0.007	0.006	0.785	0.003	0.009
2	1.2. OBF for both	A	1	0	0	0	0	0
2	1.2. OBF for both	B	0.975	0.025	0	0	0	0
2	1.2. OBF for both	C	0.946	0.024	0.03	0	0	0
2	1.2. OBF for both	D	0.211	0.007	0.007	0.775	0	0
2	1.2. OBF for both	E	0.207	0.007	0.007	0.775	0.004	0
2	1.2. OBF for both	Winner	0.198	0.007	0.007	0.775	0.004	0.009
2	1.2. Pocock for futility	A	1	0	0	0	0	0
2	1.2. Pocock for futility	B	0.972	0.028	0	0	0	0
2	1.2. Pocock for futility	C	0.947	0.026	0.027	0	0	0
2	1.2. Pocock for futility	D	0.207	0.006	0.005	0.782	0	0
2	1.2. Pocock for futility	E	0.204	0.006	0.005	0.782	0.003	0
2	1.2. Pocock for futility	Winner	0.199	0.005	0.005	0.782	0.003	0.006
4	1.2. Fixed Design	A	1	0	0	0	0	0
4	1.2. Fixed Design	B	0.977	0.023	0	0	0	0
4	1.2. Fixed Design	C	0.943	0.022	0.035	0	0	0
4	1.2. Fixed Design	D	0.2	0.007	0.008	0.785	0	0
4	1.2. Fixed Design	E	0.197	0.007	0.008	0.785	0.003	0
4	1.2. Fixed Design	Winner	0.19	0.007	0.006	0.785	0.003	0.009
4	1.2. OBF for both	A	1	0	0	0	0	0
4	1.2. OBF for both	B	0.971	0.029	0	0	0	0
4	1.2. OBF for both	C	0.939	0.026	0.035	0	0	0
4	1.2. OBF for both	D	0.206	0.006	0.009	0.779	0	0
4	1.2. OBF for both	E	0.204	0.006	0.009	0.779	0.002	0
4	1.2. OBF for both	Winner	0.199	0.006	0.008	0.779	0.002	0.006
4	1.2. Pocock for futility	A	1	0	0	0	0	0
4	1.2. Pocock for futility	B	0.97	0.03	0	0	0	0
4	1.2. Pocock for futility	C	0.948	0.03	0.022	0	0	0
4	1.2. Pocock for futility	D	0.189	0.004	0.004	0.803	0	0
4	1.2. Pocock for futility	E	0.187	0.004	0.004	0.803	0.002	0
4	1.2. Pocock for futility	Winner	0.182	0.004	0.004	0.803	0.002	0.005

Table 6: Track of Comparisons Between Arms, Scenario 1.3

Look	Boundary	Comparison	S	A	B	C	D	E
2	1.3. Fixed Design	A	1	0	0	0	0	0
2	1.3. Fixed Design	B	0.977	0.023	0	0	0	0
2	1.3. Fixed Design	C	0.943	0.022	0.035	0	0	0
2	1.3. Fixed Design	D	0.925	0.021	0.034	0.02	0	0
2	1.3. Fixed Design	E	0.906	0.021	0.034	0.02	0.019	0
2	1.3. Fixed Design	Winner	0.189	0.005	0.007	0	0.003	0.796
2	1.3. OBF for both	A	1	0	0	0	0	0
2	1.3. OBF for both	B	0.975	0.025	0	0	0	0
2	1.3. OBF for both	C	0.946	0.024	0.03	0	0	0
2	1.3. OBF for both	D	0.929	0.023	0.029	0.019	0	0
2	1.3. OBF for both	E	0.909	0.023	0.029	0.019	0.02	0
2	1.3. OBF for both	Winner	0.185	0.007	0.007	0	0.005	0.796
2	1.3. Pocock for futility	A	1	0	0	0	0	0
2	1.3. Pocock for futility	B	0.972	0.028	0	0	0	0
2	1.3. Pocock for futility	C	0.947	0.026	0.027	0	0	0
2	1.3. Pocock for futility	D	0.927	0.026	0.027	0.02	0	0
2	1.3. Pocock for futility	E	0.907	0.026	0.025	0.02	0.022	0
2	1.3. Pocock for futility	Winner	0.206	0.006	0.007	0.002	0.004	0.775
4	1.3. Fixed Design	A	1	0	0	0	0	0
4	1.3. Fixed Design	B	0.977	0.023	0	0	0	0
4	1.3. Fixed Design	C	0.943	0.022	0.035	0	0	0
4	1.3. Fixed Design	D	0.925	0.021	0.034	0.02	0	0
4	1.3. Fixed Design	E	0.906	0.021	0.034	0.02	0.019	0
4	1.3. Fixed Design	Winner	0.189	0.005	0.007	0	0.003	0.796
4	1.3. OBF for both	A	1	0	0	0	0	0
4	1.3. OBF for both	B	0.971	0.029	0	0	0	0
4	1.3. OBF for both	C	0.939	0.026	0.035	0	0	0
4	1.3. OBF for both	D	0.922	0.026	0.034	0.018	0	0
4	1.3. OBF for both	E	0.902	0.025	0.033	0.018	0.022	0
4	1.3. OBF for both	Winner	0.189	0.008	0.008	0.002	0.004	0.789
4	1.3. Pocock for futility	A	1	0	0	0	0	0
4	1.3. Pocock for futility	B	0.97	0.03	0	0	0	0
4	1.3. Pocock for futility	C	0.948	0.03	0.022	0	0	0
4	1.3. Pocock for futility	D	0.934	0.03	0.022	0.014	0	0
4	1.3. Pocock for futility	E	0.913	0.029	0.021	0.014	0.023	0
4	1.3. Pocock for futility	Winner	0.186	0.003	0.007	0.001	0.004	0.799

Table 7: Track of Comparisons Between Arms, Scenario 1.4

Look	Boundary	Comparison	S	A	B	C	D	E
2	1.4. Fixed Design	A	1	0	0	0	0	0
2	1.4. Fixed Design	B	0.977	0.023	0	0	0	0
2	1.4. Fixed Design	C	0.943	0.022	0.035	0	0	0
2	1.4. Fixed Design	D	0	0	0	1	0	0
2	1.4. Fixed Design	E	0	0	0	1	0	0
2	1.4. Fixed Design	Winner	0	0	0	1	0	0
2	1.4. OBF for both	A	1	0	0	0	0	0
2	1.4. OBF for both	B	0.975	0.025	0	0	0	0
2	1.4. OBF for both	C	0.946	0.024	0.03	0	0	0
2	1.4. OBF for both	D	0	0	0	1	0	0
2	1.4. OBF for both	E	0	0	0	1	0	0
2	1.4. OBF for both	Winner	0	0	0	1	0	0
2	1.4. Pocock for futility	A	1	0	0	0	0	0
2	1.4. Pocock for futility	B	0.972	0.028	0	0	0	0
2	1.4. Pocock for futility	C	0.947	0.026	0.027	0	0	0
2	1.4. Pocock for futility	D	0	0	0	1	0	0
2	1.4. Pocock for futility	E	0	0	0	1	0	0
2	1.4. Pocock for futility	Winner	0	0	0	1	0	0
4	1.4. Fixed Design	A	1	0	0	0	0	0
4	1.4. Fixed Design	B	0.977	0.023	0	0	0	0
4	1.4. Fixed Design	C	0.943	0.022	0.035	0	0	0
4	1.4. Fixed Design	D	0	0	0	1	0	0
4	1.4. Fixed Design	E	0	0	0	1	0	0
4	1.4. Fixed Design	Winner	0	0	0	1	0	0
4	1.4. OBF for both	A	1	0	0	0	0	0
4	1.4. OBF for both	B	0.971	0.029	0	0	0	0
4	1.4. OBF for both	C	0.939	0.026	0.035	0	0	0
4	1.4. OBF for both	D	0	0	0	1	0	0
4	1.4. OBF for both	E	0	0	0	1	0	0
4	1.4. OBF for both	Winner	0	0	0	1	0	0
4	1.4. Pocock for futility	A	1	0	0	0	0	0
4	1.4. Pocock for futility	B	0.97	0.03	0	0	0	0
4	1.4. Pocock for futility	C	0.948	0.03	0.022	0	0	0
4	1.4. Pocock for futility	D	0	0	0	1	0	0
4	1.4. Pocock for futility	E	0	0	0	1	0	0
4	1.4. Pocock for futility	Winner	0	0	0	1	0	0

Table 8: Track of Comparisons Between Arms, Scenario 2.1

Look	Boundary	Comparison	S	A	B	C	D	E
2	2.1. Fixed Design	A	1	0	0	0	0	0
2	2.1. Fixed Design	B	0.977	0.023	0	0	0	0
2	2.1. Fixed Design	C	0.19	0.004	0.806	0	0	0
2	2.1. Fixed Design	D	0.185	0.004	0.806	0.005	0	0
2	2.1. Fixed Design	E	0.041	0	0.788	0.004	0.167	0
2	2.1. Fixed Design	Winner	0.04	0	0.788	0.004	0.167	0.001
2	2.1. OBF for both	A	1	0	0	0	0	0
2	2.1. OBF for both	B	0.975	0.025	0	0	0	0
2	2.1. OBF for both	C	0.205	0.003	0.792	0	0	0
2	2.1. OBF for both	D	0.202	0.003	0.792	0.003	0	0
2	2.1. OBF for both	E	0.041	0	0.776	0.002	0.181	0
2	2.1. OBF for both	Winner	0.04	0	0.776	0.002	0.181	0.001
2	2.1. Pocock for futility	A	1	0	0	0	0	0
2	2.1. Pocock for futility	B	0.972	0.028	0	0	0	0
2	2.1. Pocock for futility	C	0.195	0.008	0.797	0	0	0
2	2.1. Pocock for futility	D	0.192	0.008	0.797	0.003	0	0
2	2.1. Pocock for futility	E	0.039	0.002	0.777	0.002	0.18	0
2	2.1. Pocock for futility	Winner	0.039	0.002	0.777	0.002	0.18	0
4	2.1. Fixed Design	A	1	0	0	0	0	0
4	2.1. Fixed Design	B	0.977	0.023	0	0	0	0
4	2.1. Fixed Design	C	0.19	0.004	0.806	0	0	0
4	2.1. Fixed Design	D	0.185	0.004	0.806	0.005	0	0
4	2.1. Fixed Design	E	0.041	0	0.788	0.004	0.167	0
4	2.1. Fixed Design	Winner	0.04	0	0.788	0.004	0.167	0.001
4	2.1. OBF for both	A	1	0	0	0	0	0
4	2.1. OBF for both	B	0.971	0.029	0	0	0	0
4	2.1. OBF for both	C	0.196	0.008	0.796	0	0	0
4	2.1. OBF for both	D	0.194	0.008	0.796	0.002	0	0
4	2.1. OBF for both	E	0.042	0.001	0.778	0.001	0.178	0
4	2.1. OBF for both	Winner	0.042	0.001	0.778	0.001	0.178	0
4	2.1. Pocock for futility	A	1	0	0	0	0	0
4	2.1. Pocock for futility	B	0.97	0.03	0	0	0	0
4	2.1. Pocock for futility	C	0.181	0.003	0.816	0	0	0
4	2.1. Pocock for futility	D	0.177	0.003	0.816	0.004	0	0
4	2.1. Pocock for futility	E	0.038	0	0.797	0.003	0.162	0
4	2.1. Pocock for futility	Winner	0.037	0	0.797	0.003	0.162	0.001

Table 9: Track of Comparisons Between Arms, Scenario 2.2

Look	Boundary	Comparison	S	A	B	C	D	E
2	2.2. Fixed Design	A	1	0	0	0	0	0
2	2.2. Fixed Design	B	0.977	0.023	0	0	0	0
2	2.2. Fixed Design	C	0	0	1	0	0	0
2	2.2. Fixed Design	D	0	0	1	0	0	0
2	2.2. Fixed Design	E	0	0	1	0	0	0
2	2.2. Fixed Design	Winner	0	0	1	0	0	0
2	2.2. OBF for both	A	1	0	0	0	0	0
2	2.2. OBF for both	B	0.975	0.025	0	0	0	0
2	2.2. OBF for both	C	0	0	1	0	0	0
2	2.2. OBF for both	D	0	0	1	0	0	0
2	2.2. OBF for both	E	0	0	1	0	0	0
2	2.2. OBF for both	Winner	0	0	1	0	0	0
2	2.2. Pocock for futility	A	1	0	0	0	0	0
2	2.2. Pocock for futility	B	0.972	0.028	0	0	0	0
2	2.2. Pocock for futility	C	0	0	1	0	0	0
2	2.2. Pocock for futility	D	0	0	1	0	0	0
2	2.2. Pocock for futility	E	0	0	1	0	0	0
2	2.2. Pocock for futility	Winner	0	0	1	0	0	0
4	2.2. Fixed Design	A	1	0	0	0	0	0
4	2.2. Fixed Design	B	0.977	0.023	0	0	0	0
4	2.2. Fixed Design	C	0	0	1	0	0	0
4	2.2. Fixed Design	D	0	0	1	0	0	0
4	2.2. Fixed Design	E	0	0	1	0	0	0
4	2.2. Fixed Design	Winner	0	0	1	0	0	0
4	2.2. OBF for both	A	1	0	0	0	0	0
4	2.2. OBF for both	B	0.971	0.029	0	0	0	0
4	2.2. OBF for both	C	0	0	1	0	0	0
4	2.2. OBF for both	D	0	0	1	0	0	0
4	2.2. OBF for both	E	0	0	1	0	0	0
4	2.2. OBF for both	Winner	0	0	1	0	0	0
4	2.2. Pocock for futility	A	1	0	0	0	0	0
4	2.2. Pocock for futility	B	0.97	0.03	0	0	0	0
4	2.2. Pocock for futility	C	0	0	1	0	0	0
4	2.2. Pocock for futility	D	0	0	1	0	0	0
4	2.2. Pocock for futility	E	0	0	1	0	0	0
4	2.2. Pocock for futility	Winner	0	0	1	0	0	0

Table 10: Track of Comparisons Between Arms, Scenario 2.3

Look	Boundary	Comparison	S	A	B	C	D	E
2	2.3. Fixed Design	A	1	0	0	0	0	0
2	2.3. Fixed Design	B	0.977	0.023	0	0	0	0
2	2.3. Fixed Design	C	0.19	0.004	0.806	0	0	0
2	2.3. Fixed Design	D	0.185	0.004	0.806	0.005	0	0
2	2.3. Fixed Design	E	0	0	0	0	1	0
2	2.3. Fixed Design	Winner	0	0	0	0	1	0
2	2.3. OBF for both	A	1	0	0	0	0	0
2	2.3. OBF for both	B	0.975	0.025	0	0	0	0
2	2.3. OBF for both	C	0.205	0.003	0.792	0	0	0
2	2.3. OBF for both	D	0.202	0.003	0.792	0.003	0	0
2	2.3. OBF for both	E	0	0	0	0	1	0
2	2.3. OBF for both	Winner	0	0	0	0	1	0
2	2.3. Pocock for futility	A	1	0	0	0	0	0
2	2.3. Pocock for futility	B	0.972	0.028	0	0	0	0
2	2.3. Pocock for futility	C	0.195	0.008	0.797	0	0	0
2	2.3. Pocock for futility	D	0.192	0.008	0.797	0.003	0	0
2	2.3. Pocock for futility	E	0	0	0	0	1	0
2	2.3. Pocock for futility	Winner	0	0	0	0	1	0
4	2.3. Fixed Design	A	1	0	0	0	0	0
4	2.3. Fixed Design	B	0.977	0.023	0	0	0	0
4	2.3. Fixed Design	C	0.19	0.004	0.806	0	0	0
4	2.3. Fixed Design	D	0.185	0.004	0.806	0.005	0	0
4	2.3. Fixed Design	E	0	0	0	0	1	0
4	2.3. Fixed Design	Winner	0	0	0	0	1	0
4	2.3. OBF for both	A	1	0	0	0	0	0
4	2.3. OBF for both	B	0.971	0.029	0	0	0	0
4	2.3. OBF for both	C	0.196	0.008	0.796	0	0	0
4	2.3. OBF for both	D	0.194	0.008	0.796	0.002	0	0
4	2.3. OBF for both	E	0	0	0	0	1	0
4	2.3. OBF for both	Winner	0	0	0	0	1	0
4	2.3. Pocock for futility	A	1	0	0	0	0	0
4	2.3. Pocock for futility	B	0.97	0.03	0	0	0	0
4	2.3. Pocock for futility	C	0.181	0.003	0.816	0	0	0
4	2.3. Pocock for futility	D	0.177	0.003	0.816	0.004	0	0
4	2.3. Pocock for futility	E	0	0	0	0	1	0
4	2.3. Pocock for futility	Winner	0	0	0	0	1	0

Table 11: Track of Comparisons Between Arms, Scenario 3.1

Look	Boundary	Comparison	S	A	B	C	D	E
2	3.1. Fixed Design	A	1	0	0	0	0	0
2	3.1. Fixed Design	B	0.982	0.018	0	0	0	0
2	3.1. Fixed Design	C	0.952	0.017	0.031	0	0	0
2	3.1. Fixed Design	D	0.929	0.015	0.03	0.026	0	0
2	3.1. Fixed Design	E	0.904	0.014	0.029	0.026	0.027	0
2	3.1. Fixed Design	Winner	0.88	0.014	0.029	0.025	0.027	0.025
2	3.1. OBF for both	A	1	0	0	0	0	0
2	3.1. OBF for both	B	0.982	0.018	0	0	0	0
2	3.1. OBF for both	C	0.953	0.017	0.03	0	0	0
2	3.1. OBF for both	D	0.929	0.017	0.03	0.024	0	0
2	3.1. OBF for both	E	0.908	0.016	0.029	0.024	0.023	0
2	3.1. OBF for both	Winner	0.885	0.016	0.029	0.023	0.023	0.024
2	3.1. Pocock for futility	A	1	0	0	0	0	0
2	3.1. Pocock for futility	B	0.977	0.023	0	0	0	0
2	3.1. Pocock for futility	C	0.952	0.023	0.025	0	0	0
2	3.1. Pocock for futility	D	0.924	0.022	0.023	0.031	0	0
2	3.1. Pocock for futility	E	0.899	0.022	0.021	0.031	0.027	0
2	3.1. Pocock for futility	Winner	0.875	0.021	0.021	0.031	0.027	0.025
4	3.1. Fixed Design	A	1	0	0	0	0	0
4	3.1. Fixed Design	B	0.982	0.018	0	0	0	0
4	3.1. Fixed Design	C	0.952	0.017	0.031	0	0	0
4	3.1. Fixed Design	D	0.929	0.015	0.03	0.026	0	0
4	3.1. Fixed Design	E	0.904	0.014	0.029	0.026	0.027	0
4	3.1. Fixed Design	Winner	0.88	0.014	0.029	0.025	0.027	0.025
4	3.1. OBF for both	A	1	0	0	0	0	0
4	3.1. OBF for both	B	0.981	0.019	0	0	0	0
4	3.1. OBF for both	C	0.952	0.018	0.03	0	0	0
4	3.1. OBF for both	D	0.923	0.017	0.03	0.03	0	0
4	3.1. OBF for both	E	0.899	0.016	0.029	0.03	0.026	0
4	3.1. OBF for both	Winner	0.881	0.016	0.029	0.03	0.025	0.019
4	3.1. Pocock for futility	A	1	0	0	0	0	0
4	3.1. Pocock for futility	B	0.97	0.03	0	0	0	0
4	3.1. Pocock for futility	C	0.935	0.03	0.035	0	0	0
4	3.1. Pocock for futility	D	0.912	0.029	0.034	0.025	0	0
4	3.1. Pocock for futility	E	0.888	0.029	0.032	0.023	0.028	0
4	3.1. Pocock for futility	Winner	0.864	0.029	0.032	0.02	0.028	0.027

Table 12: Track of Comparisons Between Arms, Scenario 3.2

Look	Boundary	Comparison	S	A	B	C	D	E
2	3.2. Fixed Design	A	1	0	0	0	0	0
2	3.2. Fixed Design	B	0.982	0.018	0	0	0	0
2	3.2. Fixed Design	C	0.952	0.017	0.031	0	0	0
2	3.2. Fixed Design	D	0.056	0	0.001	0.943	0	0
2	3.2. Fixed Design	E	0.055	0	0.001	0.943	0.001	0
2	3.2. Fixed Design	Winner	0.053	0	0.001	0.943	0.001	0.002
2	3.2. OBF for both	A	1	0	0	0	0	0
2	3.2. OBF for both	B	0.982	0.018	0	0	0	0
2	3.2. OBF for both	C	0.953	0.017	0.03	0	0	0
2	3.2. OBF for both	D	0.063	0.002	0.001	0.934	0	0
2	3.2. OBF for both	E	0.063	0.002	0.001	0.934	0	0
2	3.2. OBF for both	Winner	0.061	0.002	0.001	0.934	0	0.002
2	3.2. Pocock for futility	A	1	0	0	0	0	0
2	3.2. Pocock for futility	B	0.977	0.023	0	0	0	0
2	3.2. Pocock for futility	C	0.952	0.023	0.025	0	0	0
2	3.2. Pocock for futility	D	0.057	0	0.001	0.942	0	0
2	3.2. Pocock for futility	E	0.057	0	0.001	0.942	0	0
2	3.2. Pocock for futility	Winner	0.057	0	0.001	0.942	0	0
4	3.2. Fixed Design	A	1	0	0	0	0	0
4	3.2. Fixed Design	B	0.982	0.018	0	0	0	0
4	3.2. Fixed Design	C	0.952	0.017	0.031	0	0	0
4	3.2. Fixed Design	D	0.056	0	0.001	0.943	0	0
4	3.2. Fixed Design	E	0.055	0	0.001	0.943	0.001	0
4	3.2. Fixed Design	Winner	0.053	0	0.001	0.943	0.001	0.002
4	3.2. OBF for both	A	1	0	0	0	0	0
4	3.2. OBF for both	B	0.981	0.019	0	0	0	0
4	3.2. OBF for both	C	0.952	0.018	0.03	0	0	0
4	3.2. OBF for both	D	0.059	0.001	0.002	0.938	0	0
4	3.2. OBF for both	E	0.059	0.001	0.002	0.938	0	0
4	3.2. OBF for both	Winner	0.058	0.001	0.002	0.938	0	0.001
4	3.2. Pocock for futility	A	1	0	0	0	0	0
4	3.2. Pocock for futility	B	0.97	0.03	0	0	0	0
4	3.2. Pocock for futility	C	0.935	0.03	0.035	0	0	0
4	3.2. Pocock for futility	D	0.063	0.002	0.003	0.932	0	0
4	3.2. Pocock for futility	E	0.063	0.002	0.003	0.932	0	0
4	3.2. Pocock for futility	Winner	0.062	0.002	0.003	0.932	0	0.001

Table 13: Track of Comparisons Between Arms, Scenario 3.3

Look	Boundary	Comparison	S	A	B	C	D	E
2	3.3. Fixed Design	A	1	0	0	0	0	0
2	3.3. Fixed Design	B	0.978	0.022	0	0	0	0
2	3.3. Fixed Design	C	0.948	0.021	0.031	0	0	0
2	3.3. Fixed Design	D	0.935	0.02	0.031	0.014	0	0
2	3.3. Fixed Design	E	0.915	0.019	0.031	0.013	0.022	0
2	3.3. Fixed Design	Winner	0.893	0.018	0.031	0.013	0.022	0.023
2	3.3. OBF for both	A	1	0	0	0	0	0
2	3.3. OBF for both	B	0.987	0.013	0	0	0	0
2	3.3. OBF for both	C	0.951	0.012	0.037	0	0	0
2	3.3. OBF for both	D	0.93	0.012	0.037	0.021	0	0
2	3.3. OBF for both	E	0.912	0.012	0.036	0.02	0.02	0
2	3.3. OBF for both	Winner	0.889	0.011	0.035	0.019	0.02	0.026
2	3.3. Pocock for futility	A	1	0	0	0	0	0
2	3.3. Pocock for futility	B	0.985	0.015	0	0	0	0
2	3.3. Pocock for futility	C	0.962	0.015	0.023	0	0	0
2	3.3. Pocock for futility	D	0.949	0.014	0.023	0.014	0	0
2	3.3. Pocock for futility	E	0.931	0.014	0.023	0.013	0.019	0
2	3.3. Pocock for futility	Winner	0.903	0.014	0.023	0.011	0.019	0.03
4	3.3. Fixed Design	A	1	0	0	0	0	0
4	3.3. Fixed Design	B	0.978	0.022	0	0	0	0
4	3.3. Fixed Design	C	0.948	0.021	0.031	0	0	0
4	3.3. Fixed Design	D	0.935	0.02	0.031	0.014	0	0
4	3.3. Fixed Design	E	0.915	0.019	0.031	0.013	0.022	0
4	3.3. Fixed Design	Winner	0.893	0.018	0.031	0.013	0.022	0.023
4	3.3. OBF for both	A	1	0	0	0	0	0
4	3.3. OBF for both	B	0.979	0.021	0	0	0	0
4	3.3. OBF for both	C	0.946	0.02	0.034	0	0	0
4	3.3. OBF for both	D	0.926	0.02	0.034	0.02	0	0
4	3.3. OBF for both	E	0.905	0.02	0.034	0.019	0.022	0
4	3.3. OBF for both	Winner	0.879	0.019	0.033	0.019	0.022	0.028
4	3.3. Pocock for futility	A	1	0	0	0	0	0
4	3.3. Pocock for futility	B	0.984	0.016	0	0	0	0
4	3.3. Pocock for futility	C	0.953	0.015	0.032	0	0	0
4	3.3. Pocock for futility	D	0.932	0.015	0.032	0.021	0	0
4	3.3. Pocock for futility	E	0.906	0.014	0.032	0.02	0.028	0
4	3.3. Pocock for futility	Winner	0.883	0.014	0.032	0.018	0.027	0.026

Table 14: Track of Comparisons Between Arms, Scenario 3.4

Look	Boundary	Comparison	S	A	B	C	D	E
2	3.4. Fixed Design	A	1	0	0	0	0	0
2	3.4. Fixed Design	B	0.978	0.022	0	0	0	0
2	3.4. Fixed Design	C	0.948	0.021	0.031	0	0	0
2	3.4. Fixed Design	D	0.49	0.01	0.018	0.482	0	0
2	3.4. Fixed Design	E	0.477	0.01	0.018	0.482	0.013	0
2	3.4. Fixed Design	Winner	0.464	0.009	0.018	0.482	0.013	0.014
2	3.4. OBF for both	A	1	0	0	0	0	0
2	3.4. OBF for both	B	0.987	0.013	0	0	0	0
2	3.4. OBF for both	C	0.951	0.012	0.037	0	0	0
2	3.4. OBF for both	D	0.486	0.007	0.019	0.488	0	0
2	3.4. OBF for both	E	0.475	0.007	0.019	0.488	0.011	0
2	3.4. OBF for both	Winner	0.463	0.006	0.019	0.488	0.011	0.013
2	3.4. Pocock for futility	A	1	0	0	0	0	0
2	3.4. Pocock for futility	B	0.985	0.015	0	0	0	0
2	3.4. Pocock for futility	C	0.962	0.015	0.023	0	0	0
2	3.4. Pocock for futility	D	0.486	0.01	0.013	0.491	0	0
2	3.4. Pocock for futility	E	0.479	0.01	0.013	0.491	0.007	0
2	3.4. Pocock for futility	Winner	0.462	0.01	0.013	0.491	0.007	0.017
4	3.4. Fixed Design	A	1	0	0	0	0	0
4	3.4. Fixed Design	B	0.978	0.022	0	0	0	0
4	3.4. Fixed Design	C	0.948	0.021	0.031	0	0	0
4	3.4. Fixed Design	D	0.49	0.01	0.018	0.482	0	0
4	3.4. Fixed Design	E	0.477	0.01	0.018	0.482	0.013	0
4	3.4. Fixed Design	Winner	0.464	0.009	0.018	0.482	0.013	0.014
4	3.4. OBF for both	A	1	0	0	0	0	0
4	3.4. OBF for both	B	0.979	0.021	0	0	0	0
4	3.4. OBF for both	C	0.946	0.02	0.034	0	0	0
4	3.4. OBF for both	D	0.475	0.013	0.018	0.494	0	0
4	3.4. OBF for both	E	0.466	0.013	0.018	0.494	0.009	0
4	3.4. OBF for both	Winner	0.451	0.013	0.018	0.494	0.009	0.015
4	3.4. Pocock for futility	A	1	0	0	0	0	0
4	3.4. Pocock for futility	B	0.984	0.016	0	0	0	0
4	3.4. Pocock for futility	C	0.953	0.015	0.032	0	0	0
4	3.4. Pocock for futility	D	0.465	0.006	0.024	0.505	0	0
4	3.4. Pocock for futility	E	0.455	0.006	0.024	0.505	0.01	0
4	3.4. Pocock for futility	Winner	0.437	0.006	0.024	0.505	0.01	0.018

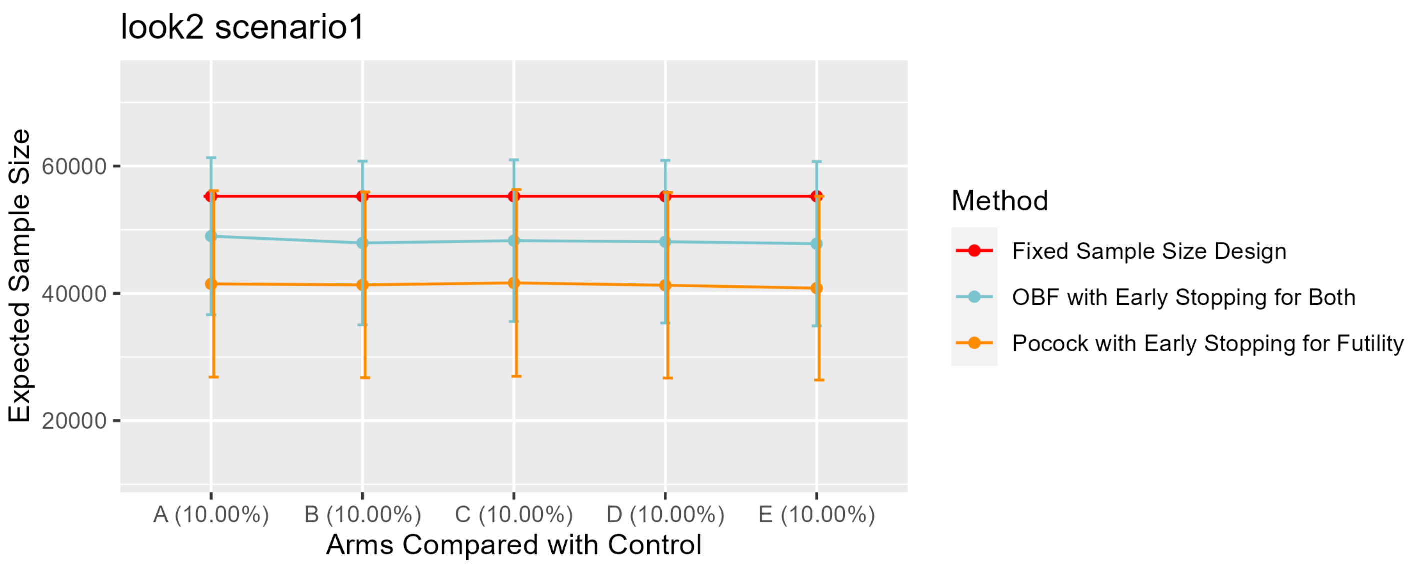
look2 scenario1
Expected Sample Size
60000
40000
20000
A (10.00%)
B (10.00%)
C (10.00%)
D (10.00%)
E (10.00%)
Arms Compared with Control
Method
Fixed Sample Size Design
OBF with Early Stopping for Both
Pocock with Early Stopping for Futility

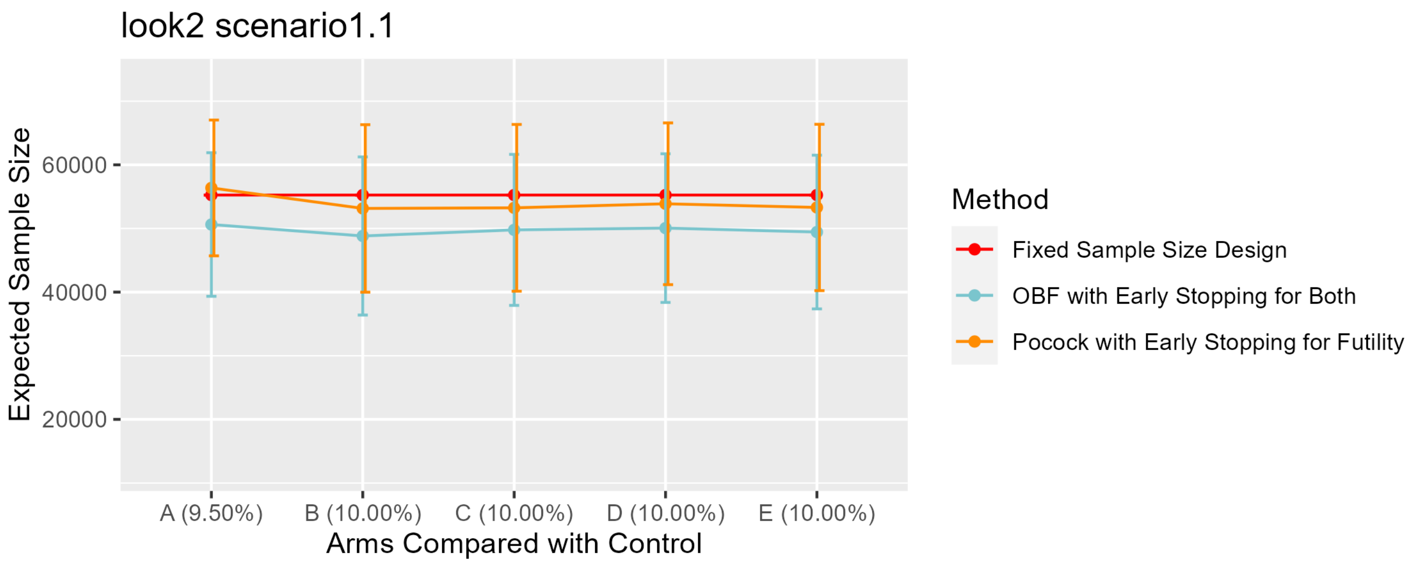
look2 scenario1.1
Expected Sample Size
60000
40000
20000
A (9.50%)
B (10.00%)
C (10.00%)
D (10.00%)
E (10.00%)
Arms Compared with Control
Method
Fixed Sample Size Design
OBF with Early Stopping for Both
Pocock with Early Stopping for Futility

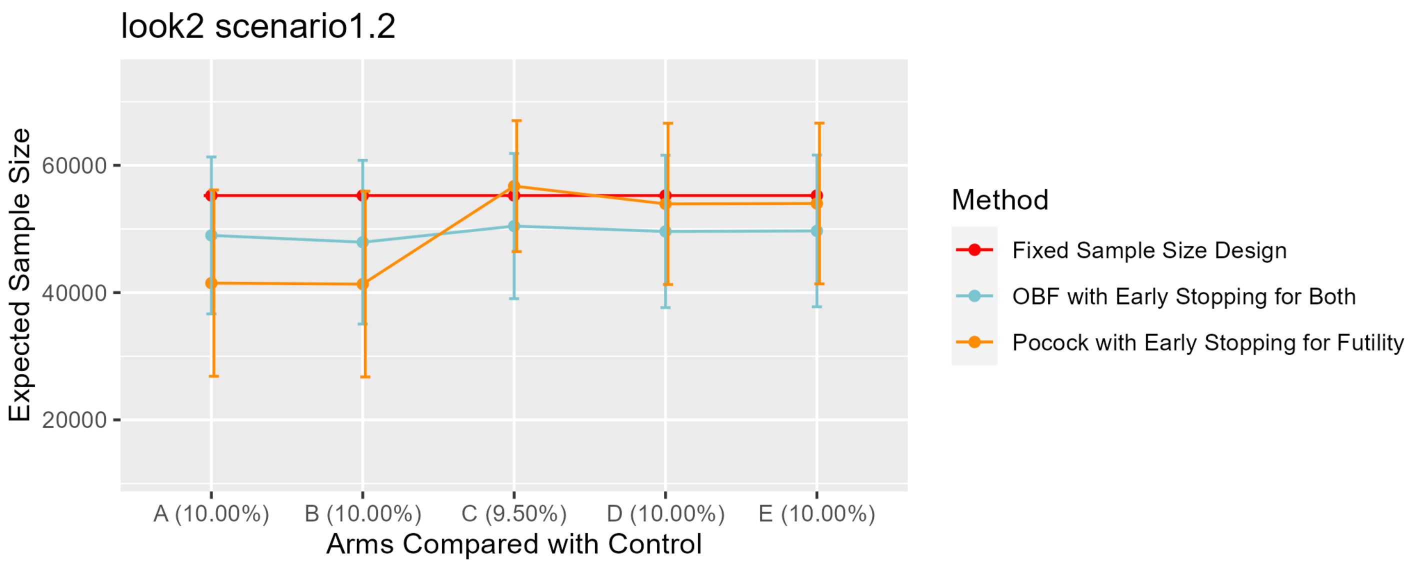
look2 scenario1.2
Expected Sample Size
60000
40000
20000
A (10.00%)
B (10.00%)
C (9.50%)
D (10.00%)
E (10.00%)
Arms Compared with Control
Method
Fixed Sample Size Design
OBF with Early Stopping for Both
Pocock with Early Stopping for Futility

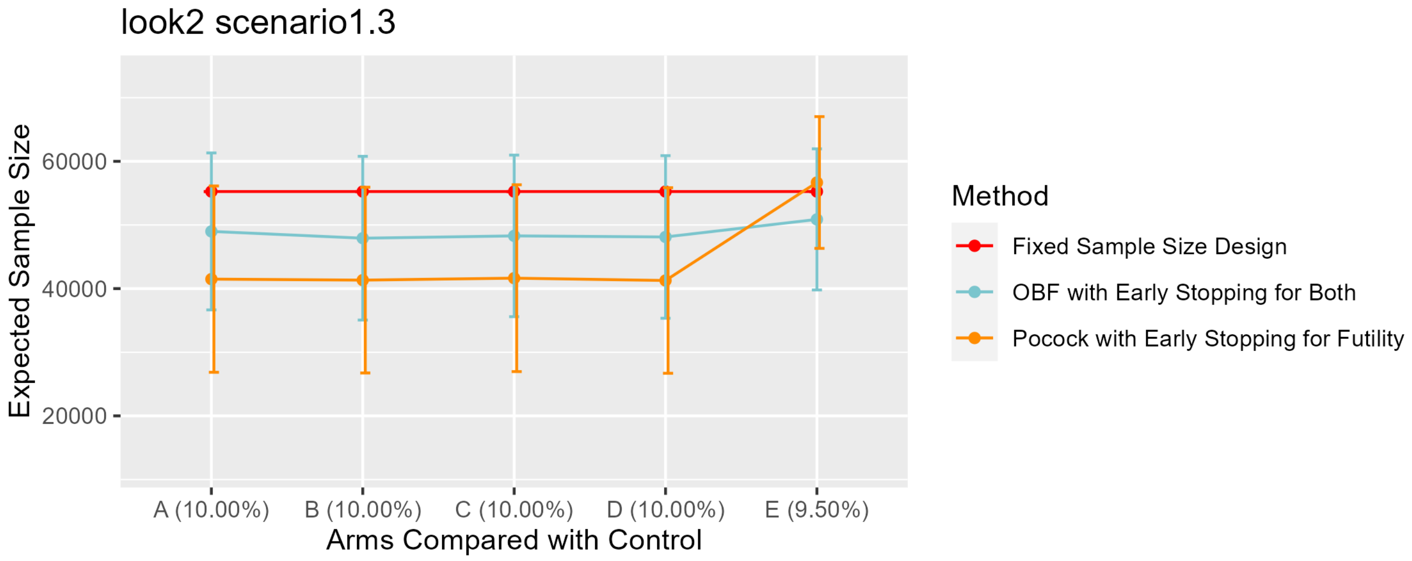
look2 scenario1.3
Expected Sample Size
60000
40000
20000
A (10.00%)
B (10.00%)
C (10.00%)
D (10.00%)
E (9.50%)
Arms Compared with Control
Method
Fixed Sample Size Design
OBF with Early Stopping for Both
Pocock with Early Stopping for Futility

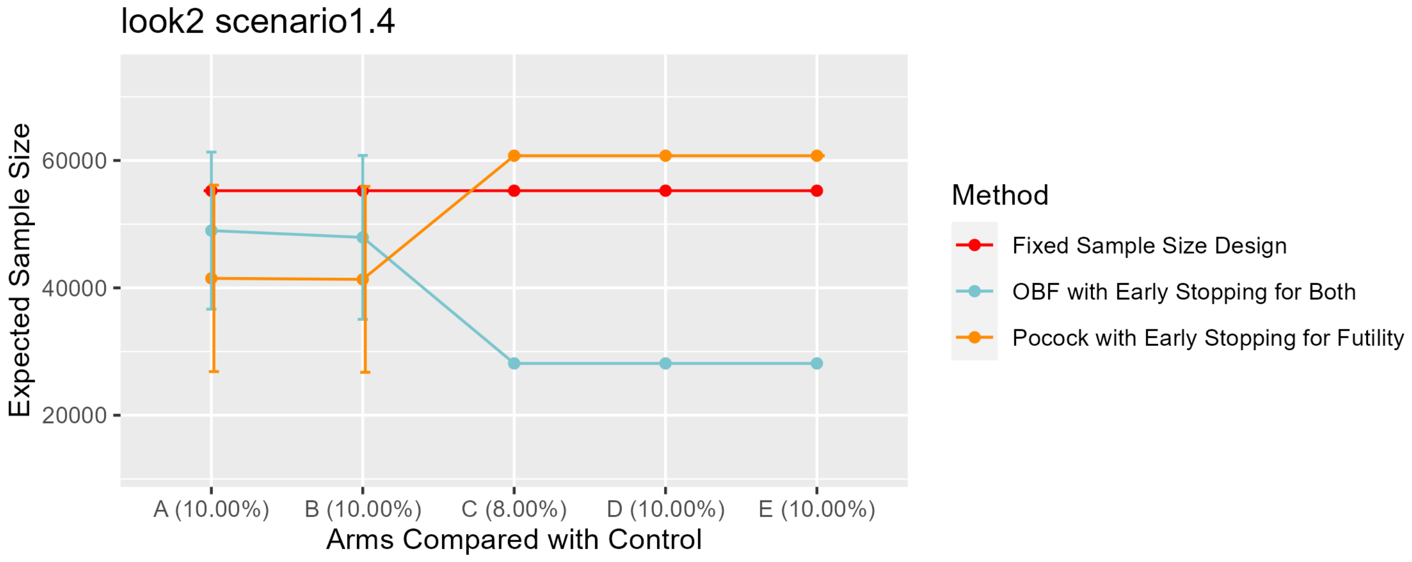
look2 scenario1.4
Expected Sample Size
60000
40000
20000
A (10.00%)
B (10.00%)
C (8.00%)
D (10.00%)
E (10.00%)
Arms Compared with Control
Method
Fixed Sample Size Design
OBF with Early Stopping for Both
Pocock with Early Stopping for Futility

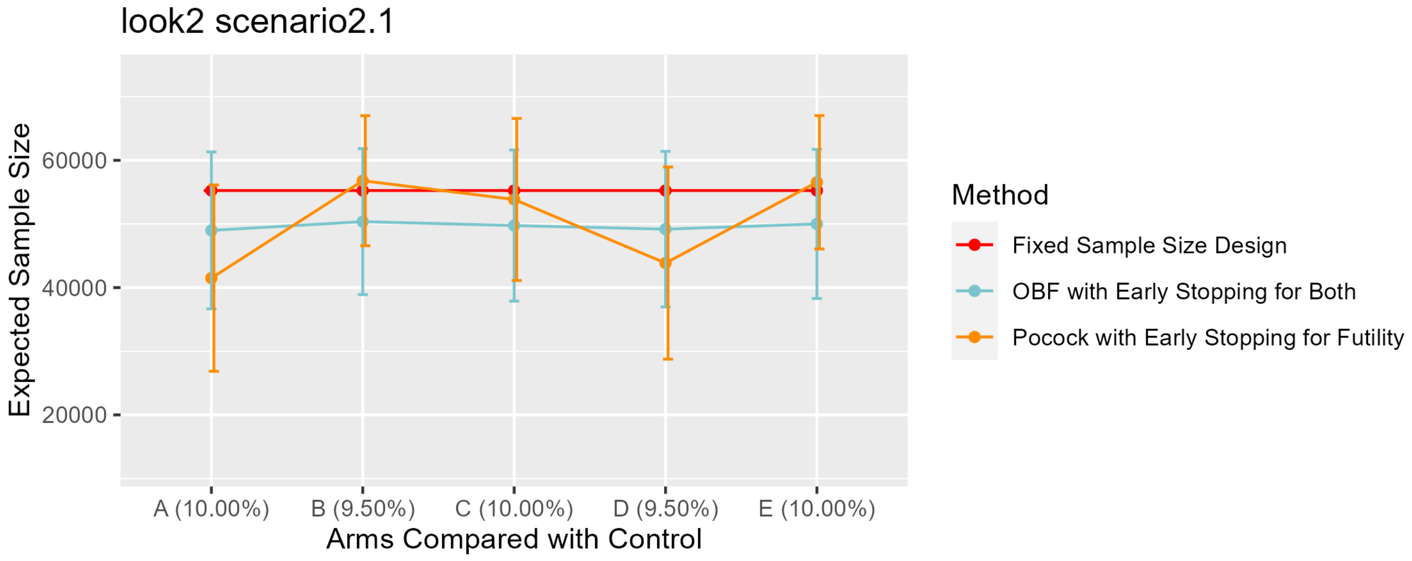
look2 scenario2.1
Expected Sample Size
60000
40000
20000
A (10.00%)
B (9.50%)
C (10.00%)
D (9.50%)
E (10.00%)
Arms Compared with Control
Method
Fixed Sample Size Design
OBF with Early Stopping for Both
Pocock with Early Stopping for Futility

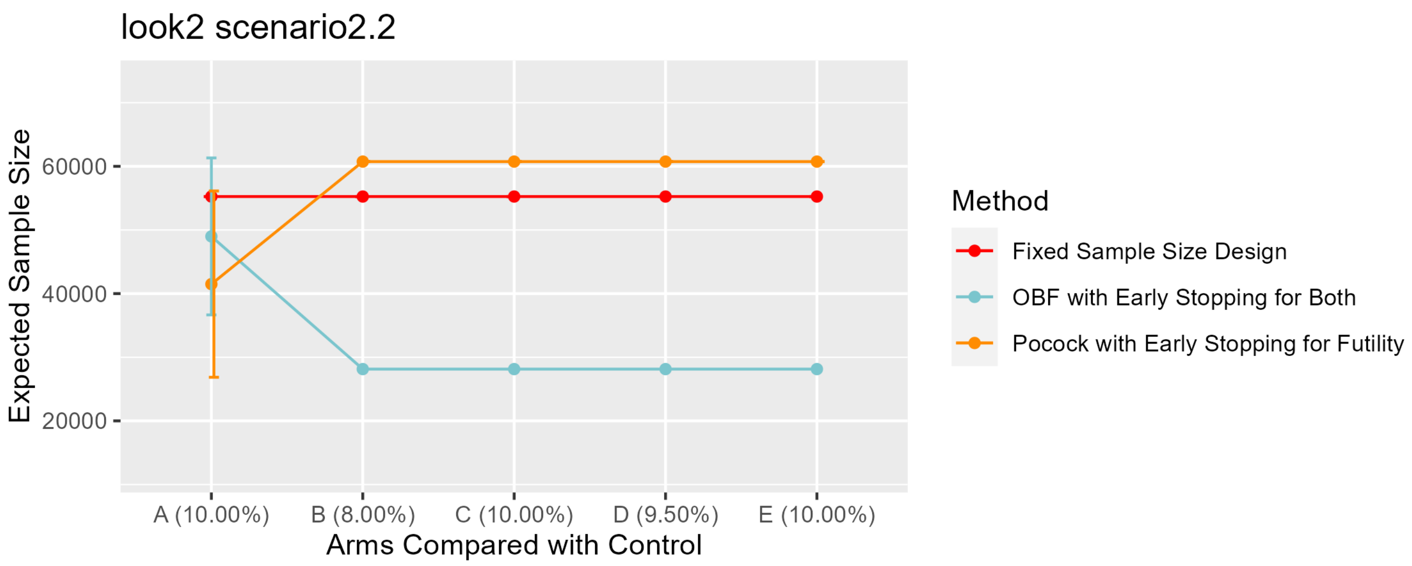
look2 scenario2.2
Expected Sample Size
60000
40000
20000
A (10.00%)
B (8.00%)
C (10.00%)
D (9.50%)
E (10.00%)
Arms Compared with Control
Method
Fixed Sample Size Design
OBF with Early Stopping for Both
Pocock with Early Stopping for Futility

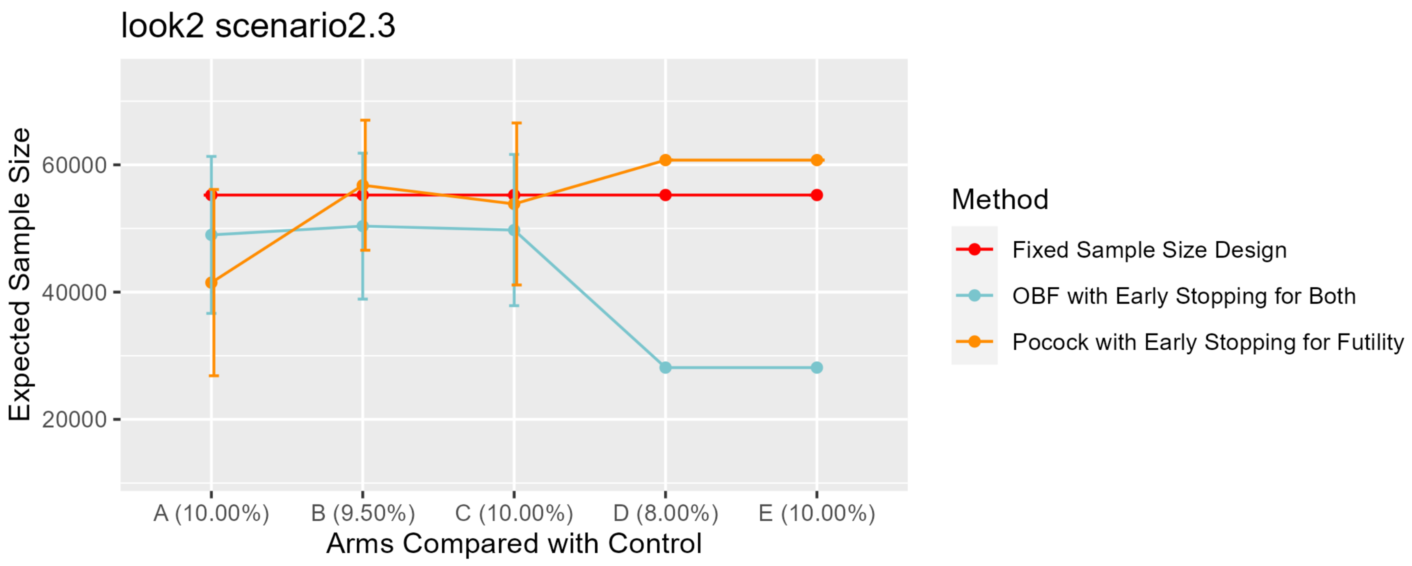
look2 scenario2.3
Expected Sample Size
60000
40000
20000
A (10.00%)
B (9.50%)
C (10.00%)
D (8.00%)
E (10.00%)
Arms Compared with Control
Method
Fixed Sample Size Design
OBF with Early Stopping for Both
Pocock with Early Stopping for Futility

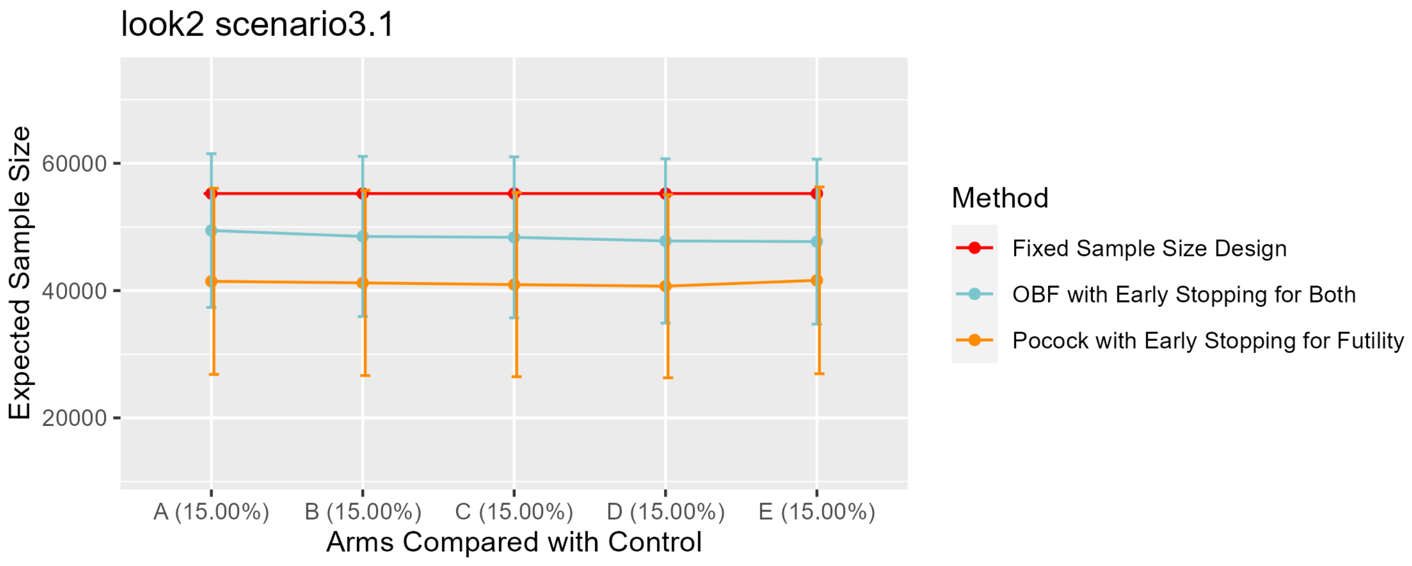
look2 scenario3.1
Expected Sample Size
60000
40000
20000
A (15.00%)
B (15.00%)
C (15.00%)
D (15.00%)
E (15.00%)
Arms Compared with Control
Method
Fixed Sample Size Design
OBF with Early Stopping for Both
Pocock with Early Stopping for Futility

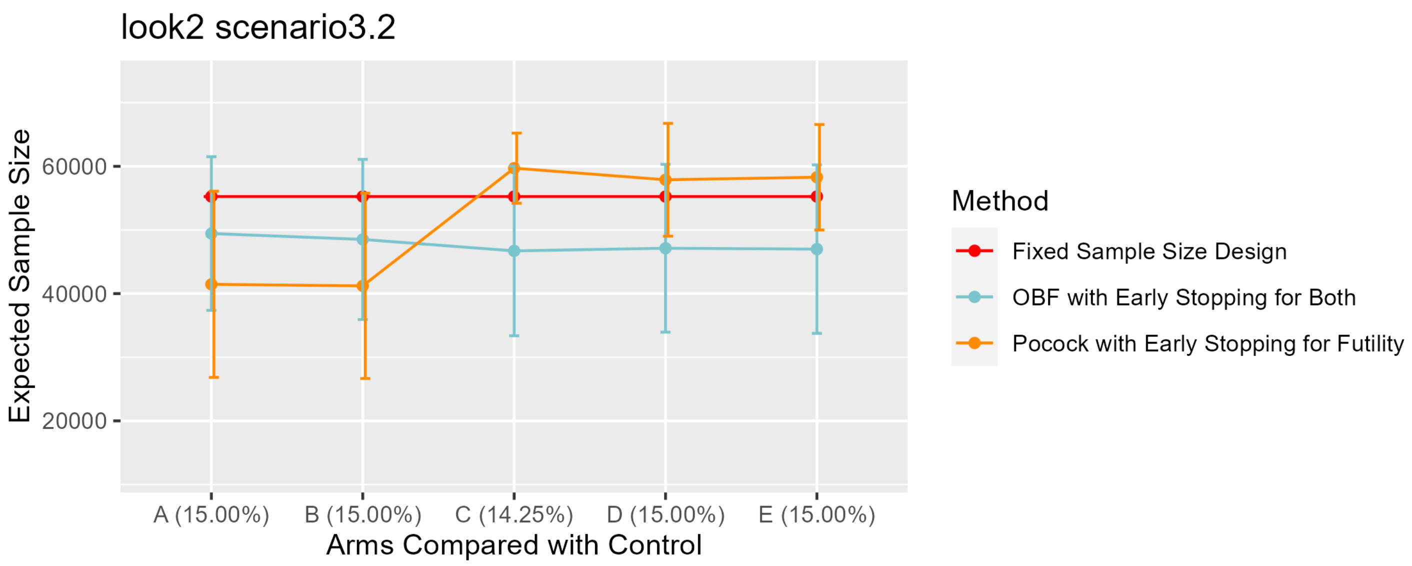
look2 scenario3.2
Expected Sample Size
60000
40000
20000
A (15.00%)
B (15.00%)
C (14.25%)
D (15.00%)
E (15.00%)
Arms Compared with Control
Method
Fixed Sample Size Design
OBF with Early Stopping for Both
Pocock with Early Stopping for Futility

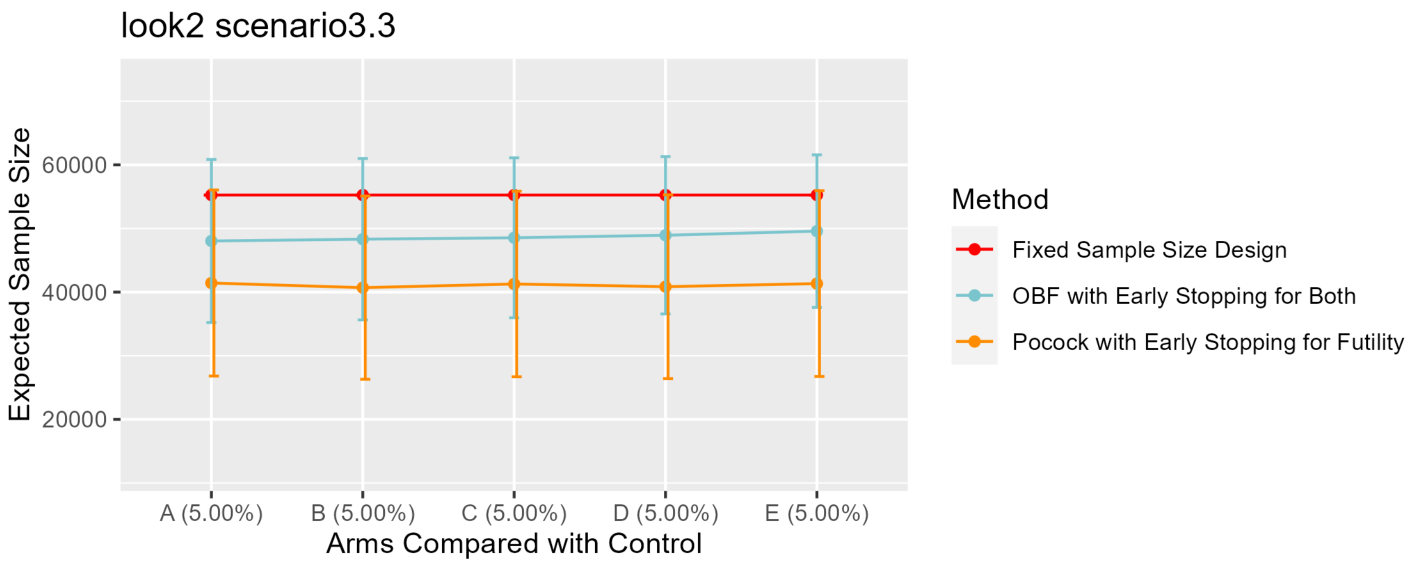
look2 scenario3.3
Expected Sample Size
60000
40000
20000
A (5.00%)
B (5.00%)
C (5.00%)
D (5.00%)
E (5.00%)
Arms Compared with Control
Method
Fixed Sample Size Design
OBF with Early Stopping for Both
Pocock with Early Stopping for Futility

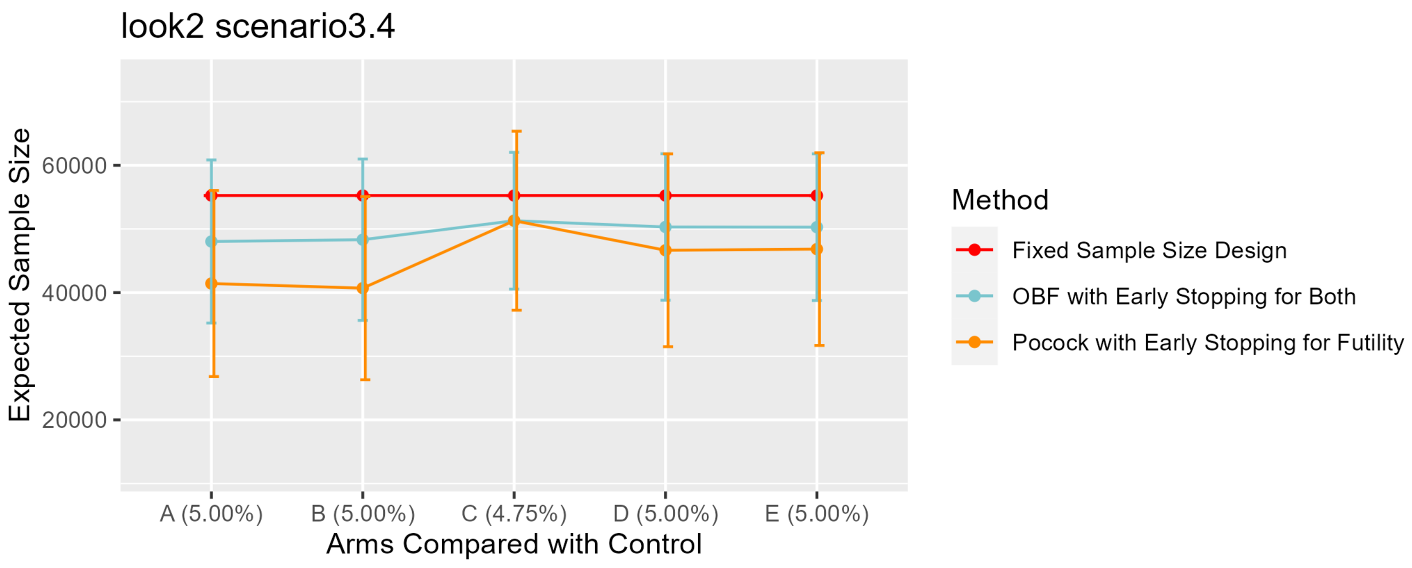
look2 scenario3.4
Expected Sample Size
60000
40000
20000
A (5.00%)
B (5.00%)
C (4.75%)
D (5.00%)
E (5.00%)
Arms Compared with Control
Method
Fixed Sample Size Design
OBF with Early Stopping for Both
Pocock with Early Stopping for Futility

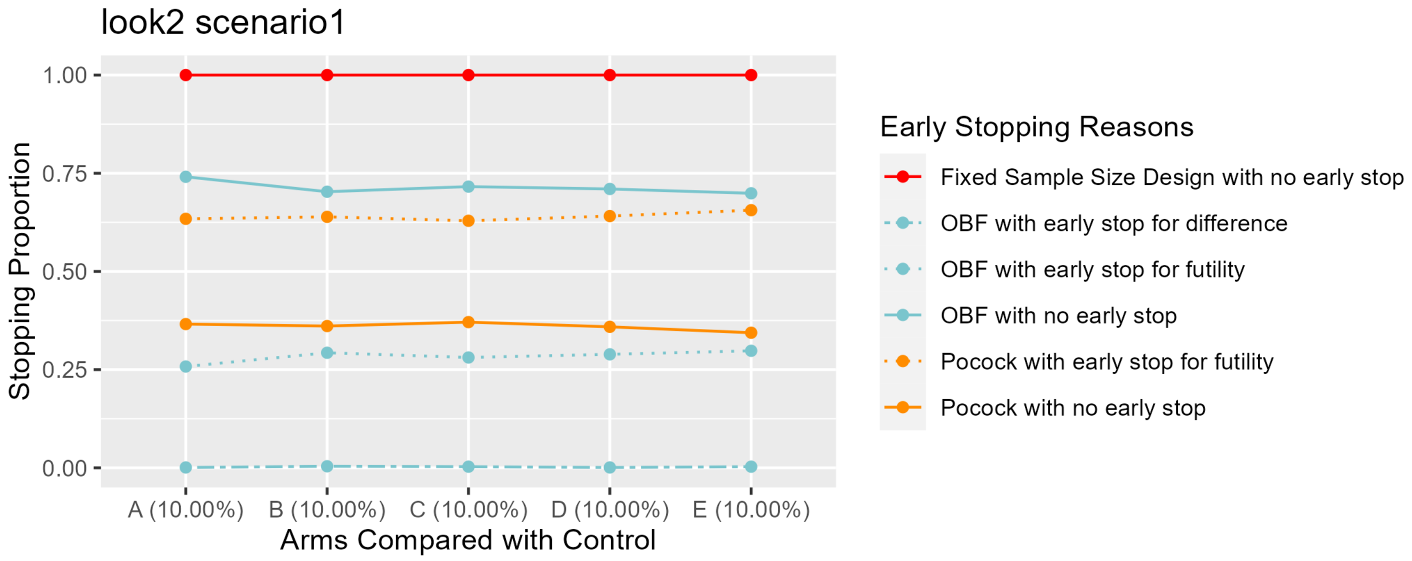
look2 scenario1
Stopping Proportion
1.00
0.75
0.50
0.25
0.00
A (10.00%)
B (10.00%)
C (10.00%)
D (10.00%)
E (10.00%)
Arms Compared with Control
Early Stopping Reasons
Fixed Sample Size Design with no early stop
OBF with early stop for difference
OBF with early stop for futility
OBF with no early stop
Pocock with early stop for futility
Pocock with no early stop

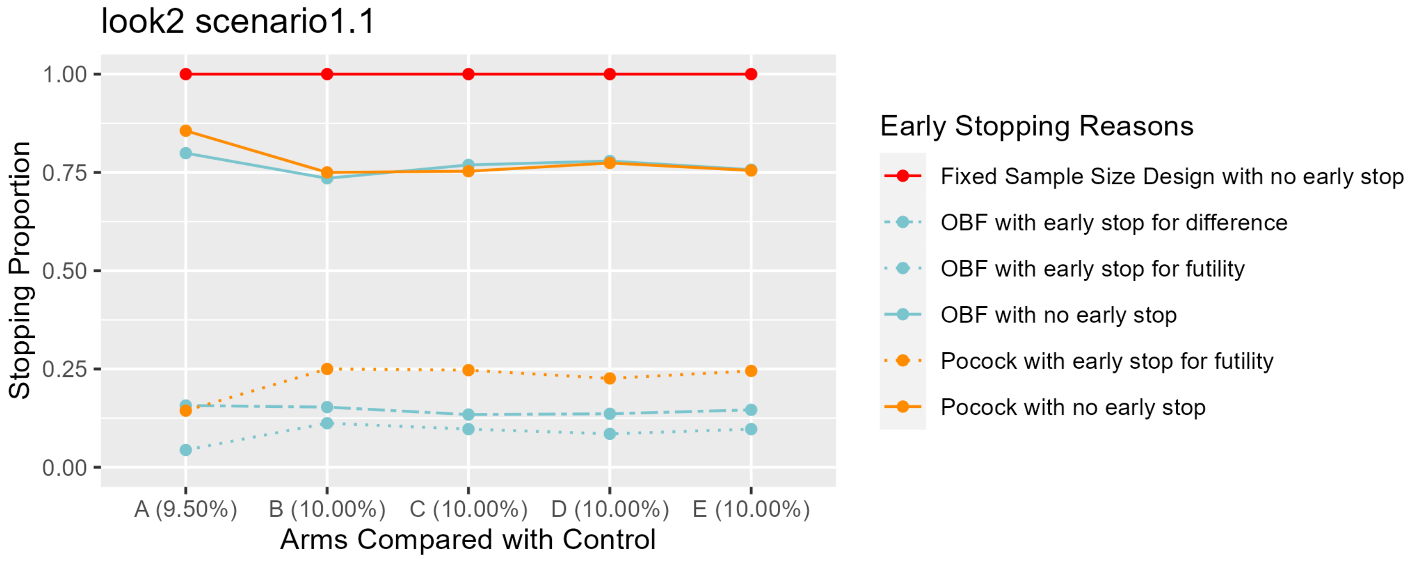
look2 scenario1.1
Stopping Proportion
1.00
0.75
0.50
0.25
0.00
A (9.50%)
B (10.00%)
C (10.00%)
D (10.00%)
E (10.00%)
Arms Compared with Control
Early Stopping Reasons
Fixed Sample Size Design with no early stop
OBF with early stop for difference
OBF with early stop for futility
OBF with no early stop
Pocock with early stop for futility
Pocock with no early stop

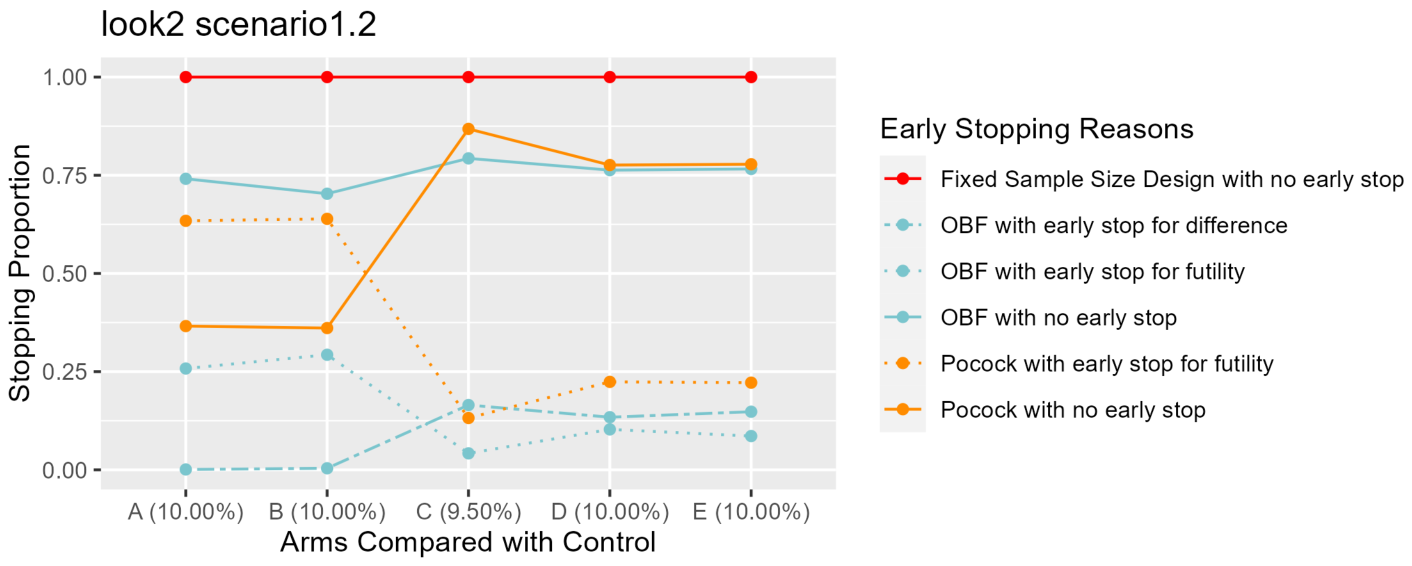
look2 scenario1.2
Stopping Proportion
1.00
0.75
0.50
0.25
0.00
A (10.00%)
B (10.00%)
C (9.50%)
D (10.00%)
E (10.00%)
Arms Compared with Control
Early Stopping Reasons
Fixed Sample Size Design with no early stop
OBF with early stop for difference
OBF with early stop for futility
OBF with no early stop
Pocock with early stop for futility
Pocock with no early stop

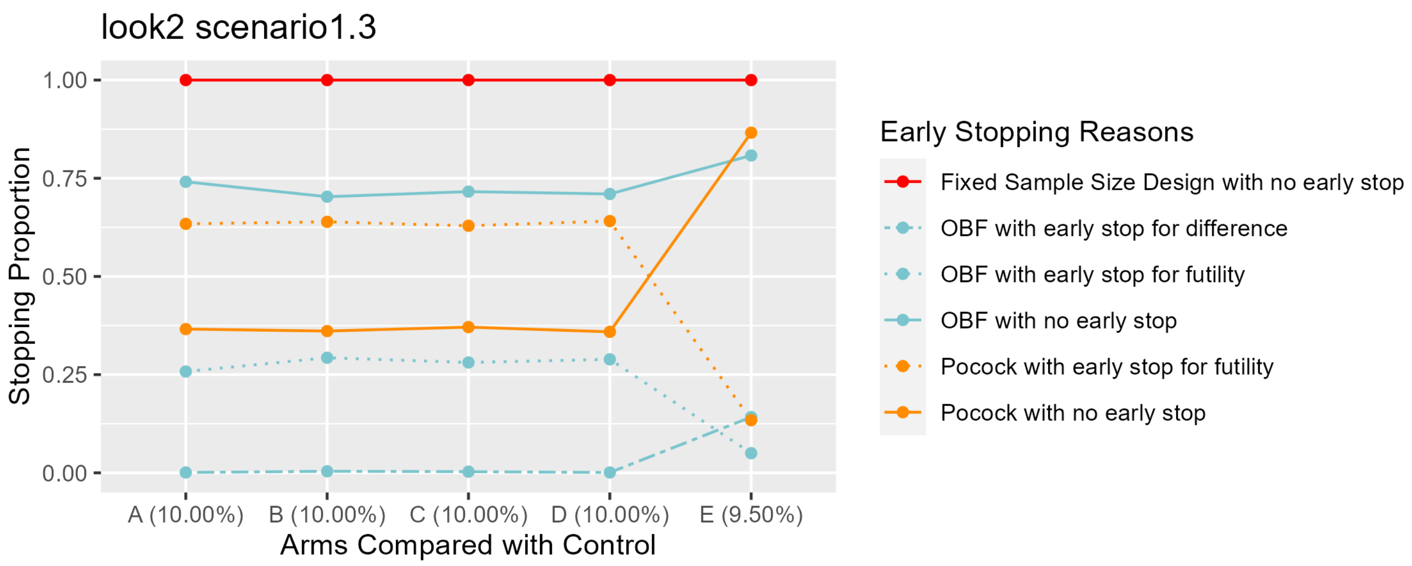
look2 scenario1.3
Stopping Proportion
1.00
0.75
0.50
0.25
0.00
A (10.00%)
B (10.00%)
C (10.00%)
D (10.00%)
E (9.50%)
Arms Compared with Control
Early Stopping Reasons
Fixed Sample Size Design with no early stop
OBF with early stop for difference
OBF with early stop for futility
OBF with no early stop
Pocock with early stop for futility
Pocock with no early stop

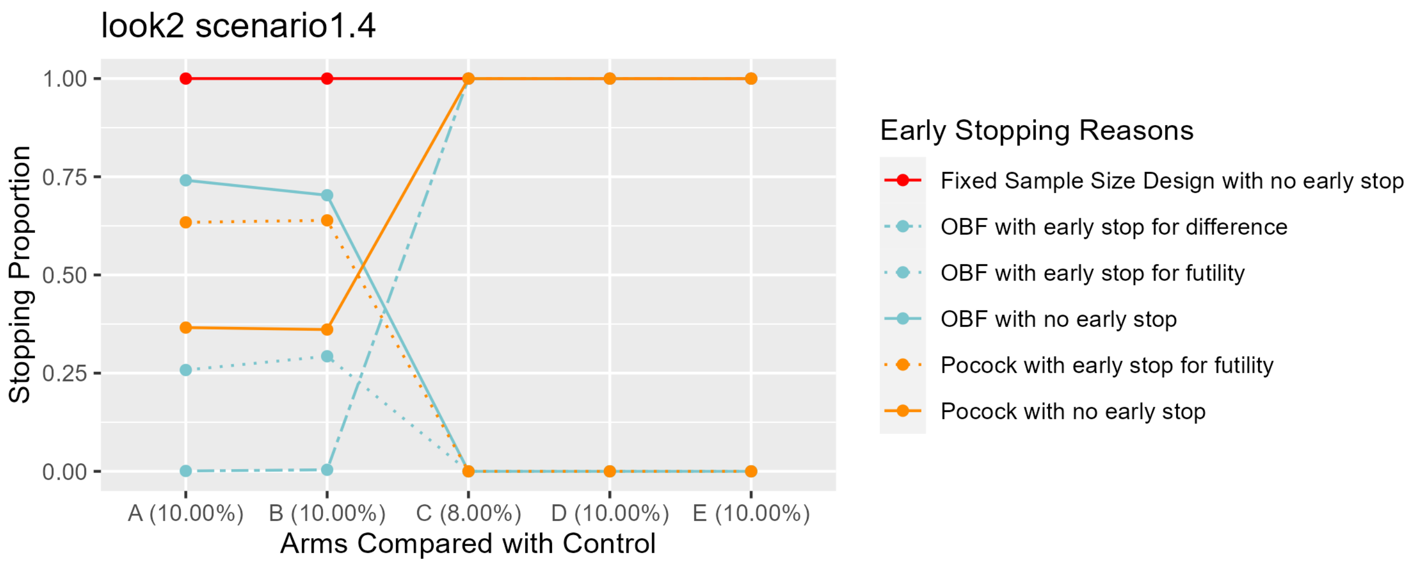
look2 scenario1.4
Stopping Proportion
1.00
0.75
0.50
0.25
0.00
A (10.00%)
B (10.00%)
C (8.00%)
D (10.00%)
E (10.00%)
Arms Compared with Control
Early Stopping Reasons
Fixed Sample Size Design with no early stop
OBF with early stop for difference
OBF with early stop for futility
OBF with no early stop
Pocock with early stop for futility
Pocock with no early stop

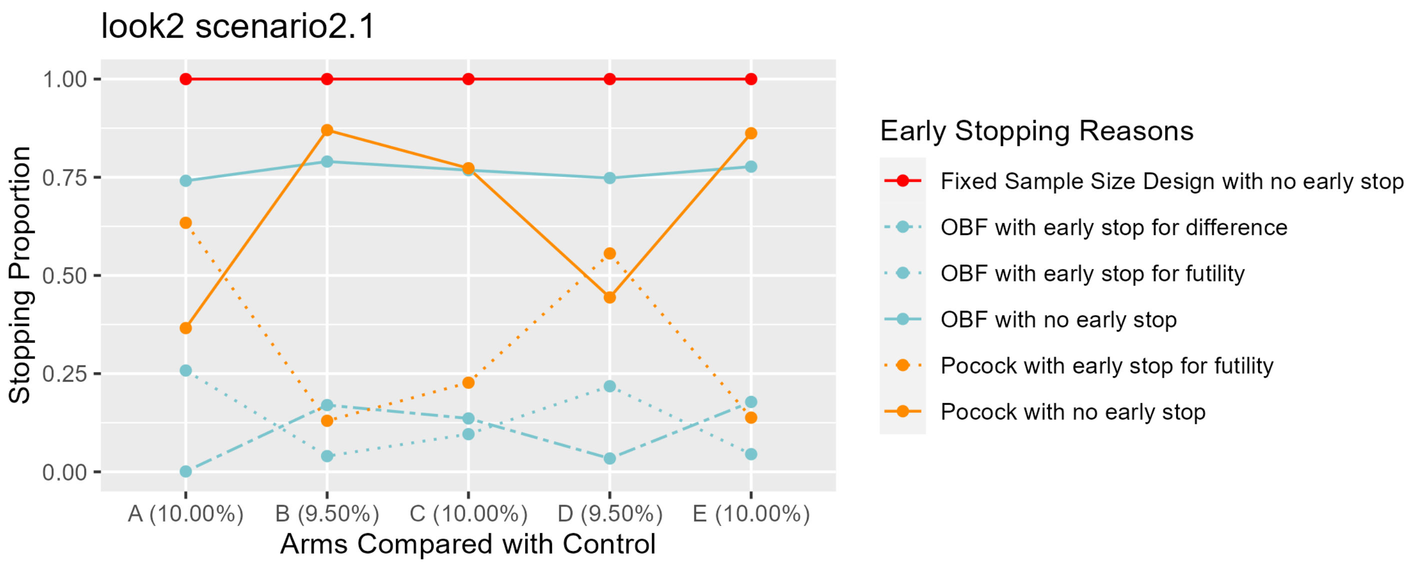
look2 scenario2.1
Stopping Proportion
1.00
0.75
0.50
0.25
0.00
A (10.00%)
B (9.50%)
C (10.00%)
D (9.50%)
E (10.00%)
Arms Compared with Control
Early Stopping Reasons
Fixed Sample Size Design with no early stop
OBF with early stop for difference
OBF with early stop for futility
OBF with no early stop
Pocock with early stop for futility
Pocock with no early stop

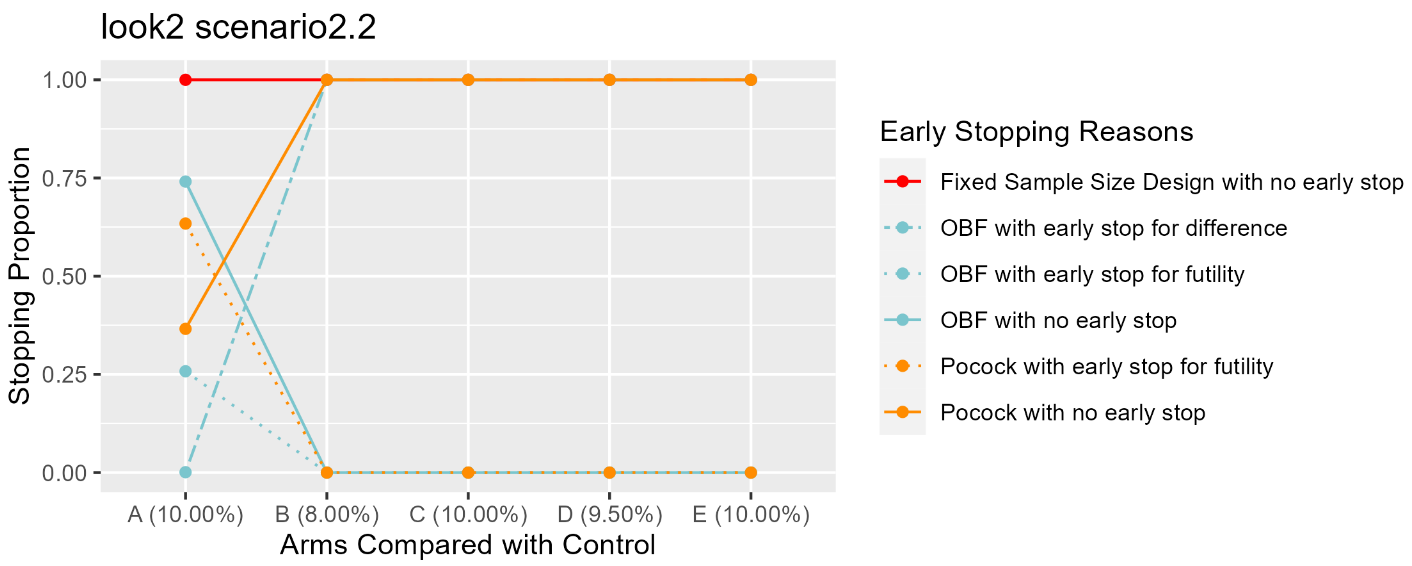
look2 scenario2.2
Stopping Proportion
1.00
0.75
0.50
0.25
0.00
A (10.00%)
B (8.00%)
C (10.00%)
D (9.50%)
E (10.00%)
Arms Compared with Control
Early Stopping Reasons
Fixed Sample Size Design with no early stop
OBF with early stop for difference
OBF with early stop for futility
OBF with no early stop
Pocock with early stop for futility
Pocock with no early stop

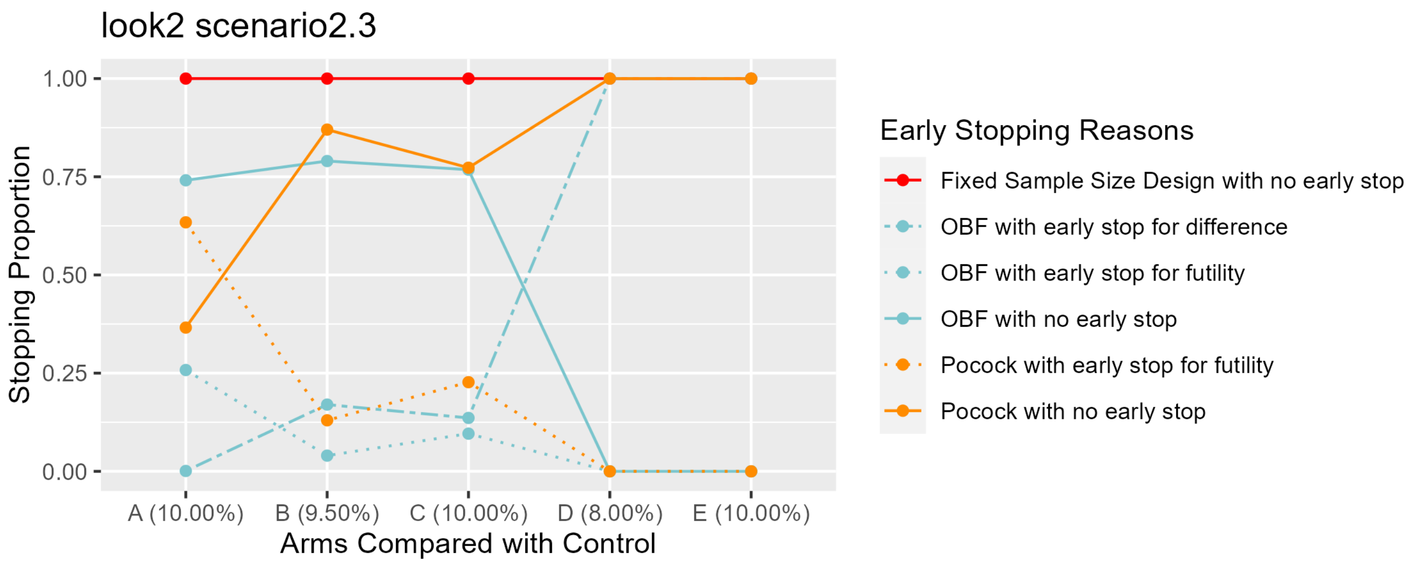
look2 scenario2.3
Stopping Proportion
1.00
0.75
0.50
0.25
0.00
A (10.00%)
B (9.50%)
C (10.00%)
D (8.00%)
E (10.00%)
Arms Compared with Control
Early Stopping Reasons
Fixed Sample Size Design with no early stop
OBF with early stop for difference
OBF with early stop for futility
OBF with no early stop
Pocock with early stop for futility
Pocock with no early stop

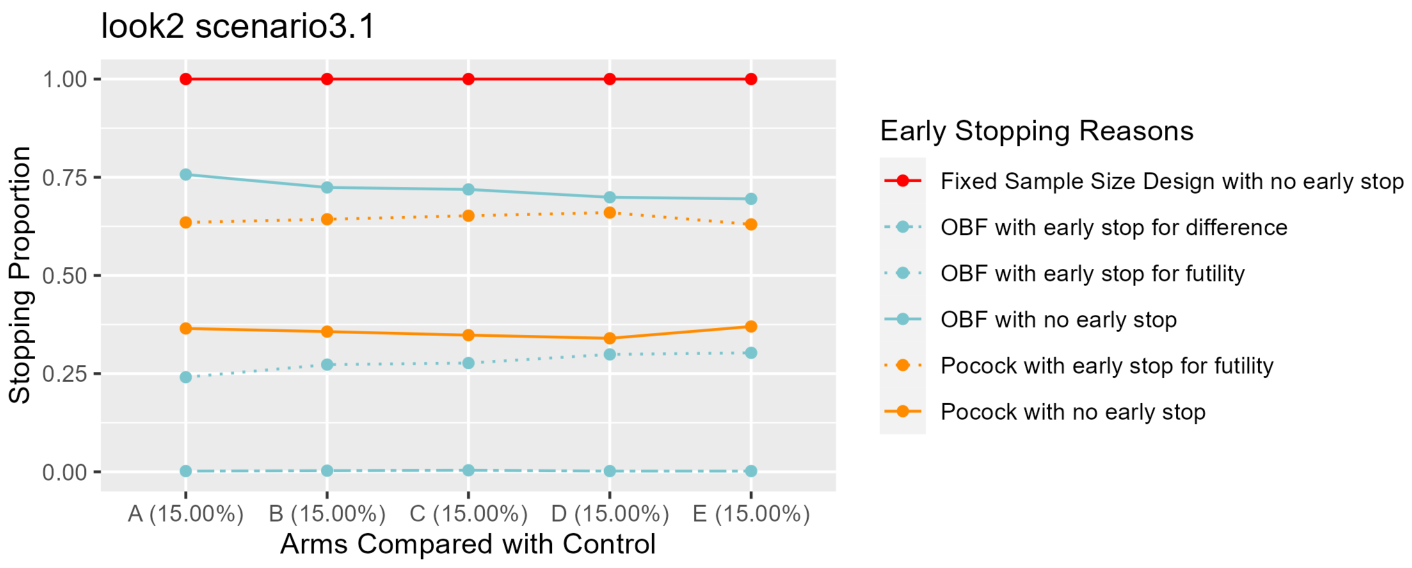
look2 scenario3.1
Stopping Proportion
1.00
0.75
0.50
0.25
0.00
A (15.00%)
B (15.00%)
C (15.00%)
D (15.00%)
E (15.00%)
Arms Compared with Control
Early Stopping Reasons
Fixed Sample Size Design with no early stop
OBF with early stop for difference
OBF with early stop for futility
OBF with no early stop
Pocock with early stop for futility
Pocock with no early stop

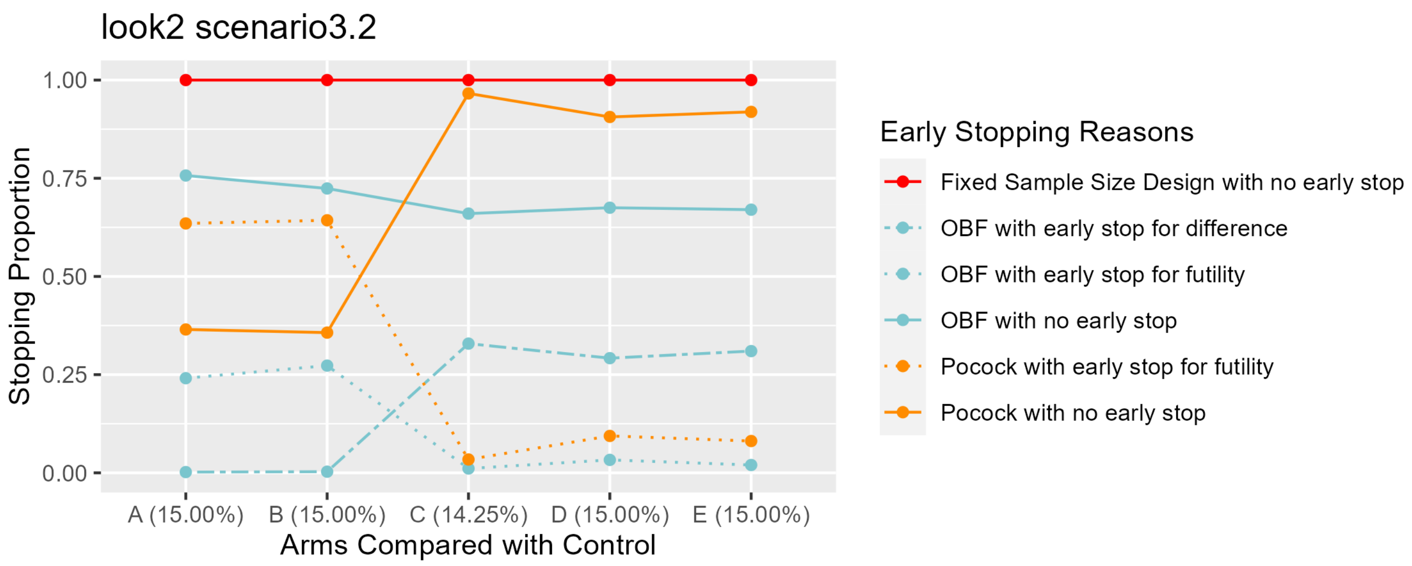
look2 scenario3.2
Stopping Proportion
1.00
0.75
0.50
0.25
0.00
A (15.00%)
B (15.00%)
C (14.25%)
D (15.00%)
E (15.00%)
Arms Compared with Control
Early Stopping Reasons
Fixed Sample Size Design with no early stop
OBF with early stop for difference
OBF with early stop for futility
OBF with no early stop
Pocock with early stop for futility
Pocock with no early stop

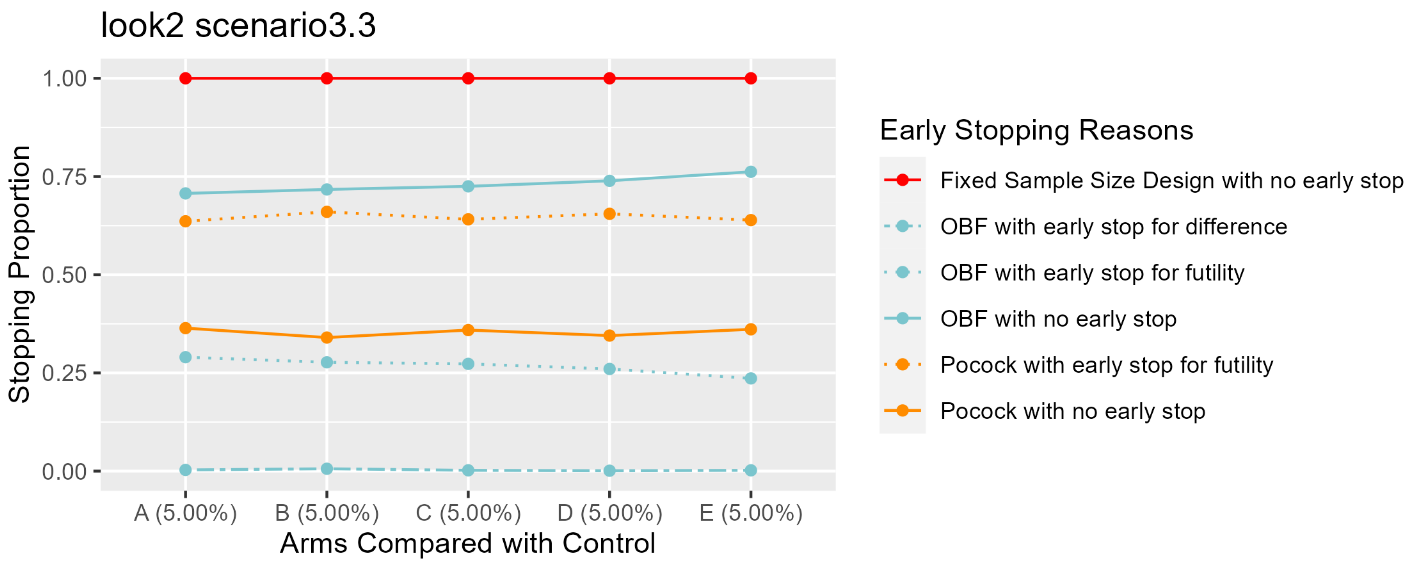
look2 scenario3.3
Stopping Proportion
1.00
0.75
0.50
0.25
0.00
A (5.00%)
B (5.00%)
C (5.00%)
D (5.00%)
E (5.00%)
Arms Compared with Control
Early Stopping Reasons
Fixed Sample Size Design with no early stop
OBF with early stop for difference
OBF with early stop for futility
OBF with no early stop
Pocock with early stop for futility
Pocock with no early stop

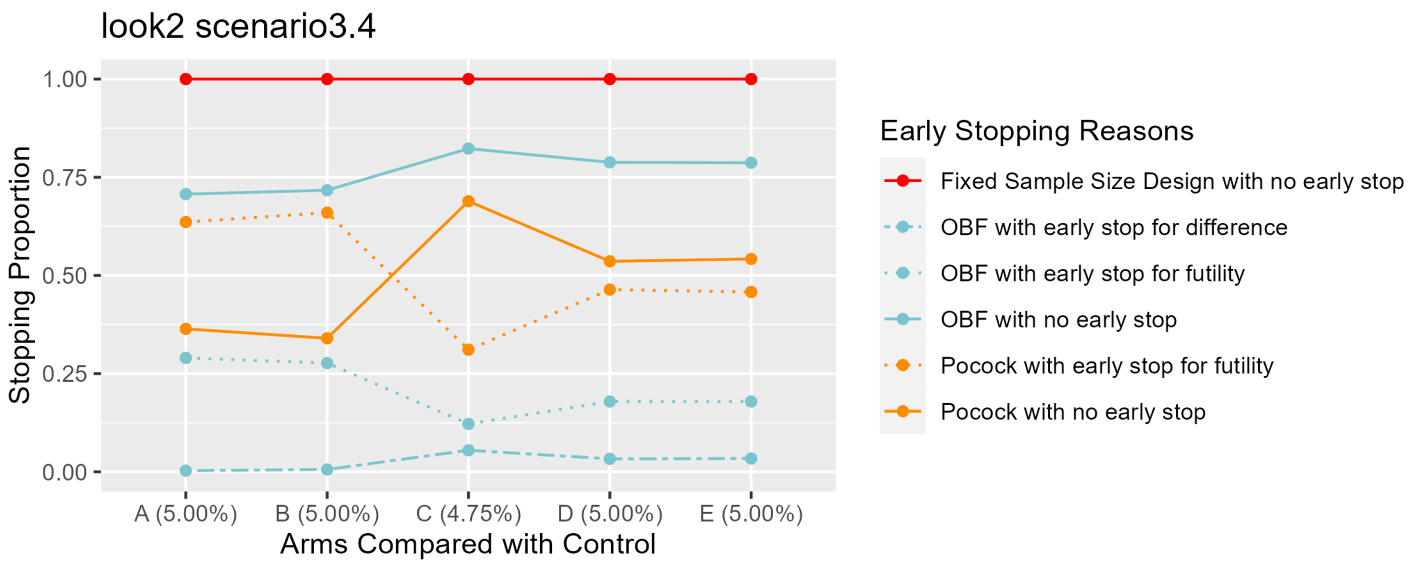
look2 scenario3.4
Stopping Proportion
1.00
0.75
0.50
0.25
0.00
A (5.00%)
B (5.00%)
C (4.75%)
D (5.00%)
E (5.00%)
Arms Compared with Control
Early Stopping Reasons
Fixed Sample Size Design with no early stop
OBF with early stop for difference
OBF with early stop for futility
OBF with no early stop
Pocock with early stop for futility
Pocock with no early stop

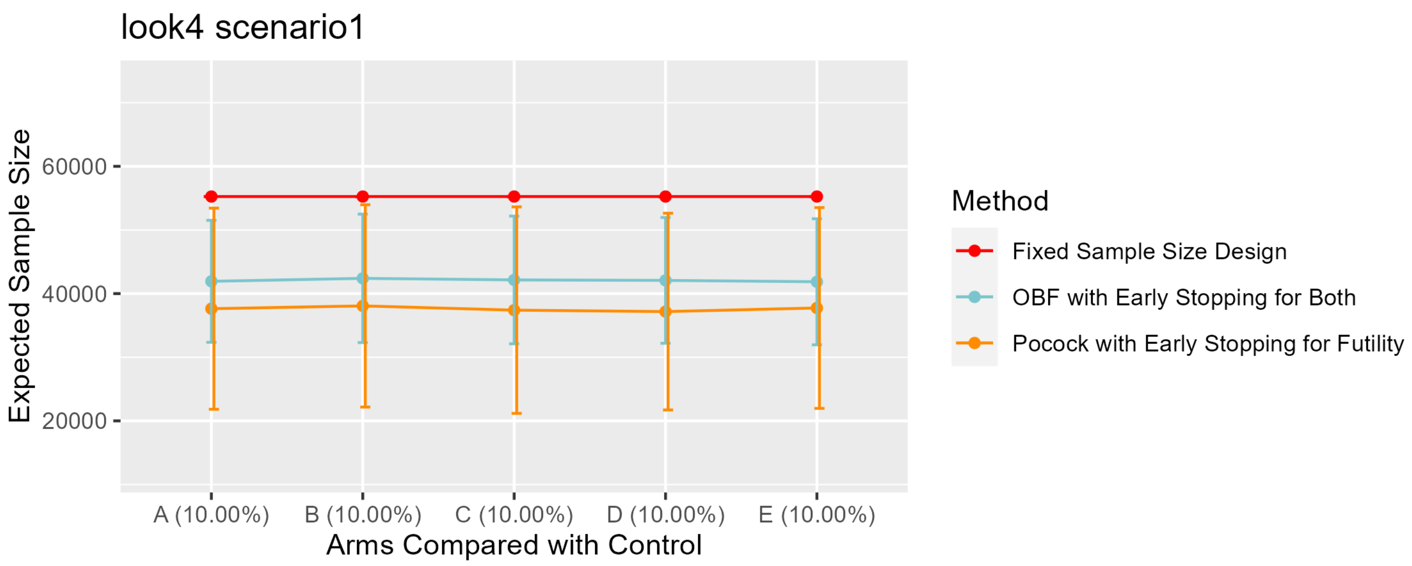
look4 scenario1
Expected Sample Size
60000
40000
20000
A (10.00%)
B (10.00%)
C (10.00%)
D (10.00%)
E (10.00%)
Arms Compared with Control
Method
Fixed Sample Size Design
OBF with Early Stopping for Both
Pocock with Early Stopping for Futility

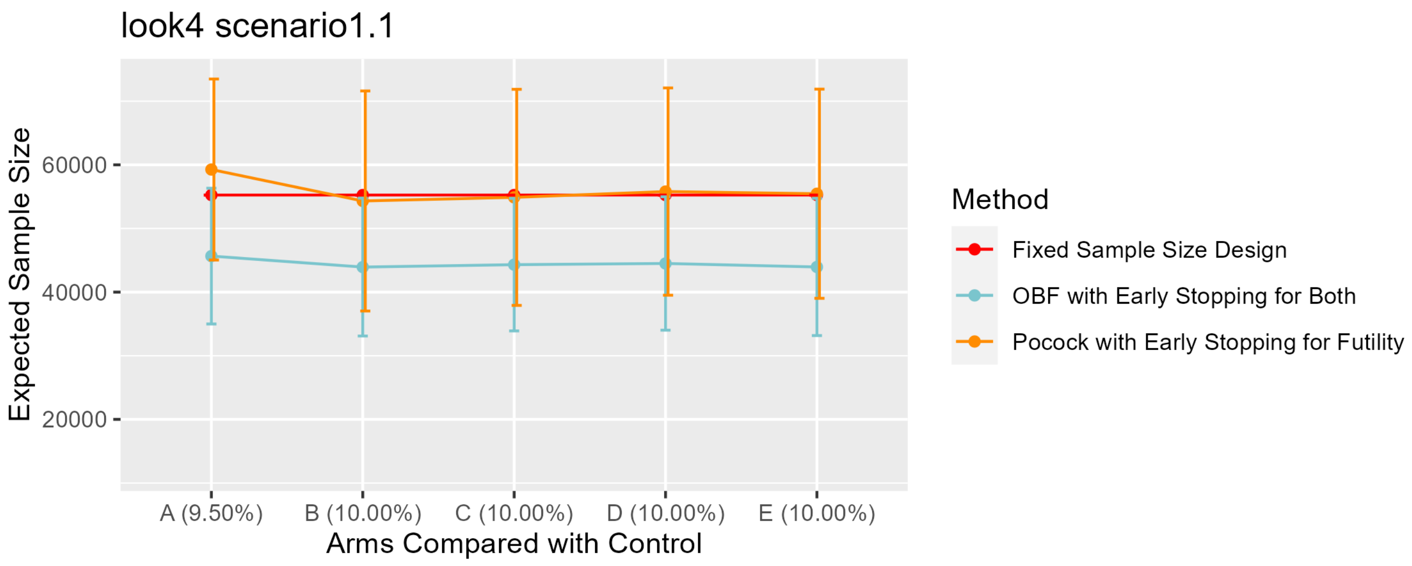
look4 scenario1.1
Expected Sample Size
60000
40000
20000
A (9.50%)
B (10.00%)
C (10.00%)
D (10.00%)
E (10.00%)
Arms Compared with Control
Method
Fixed Sample Size Design
OBF with Early Stopping for Both
Pocock with Early Stopping for Futility

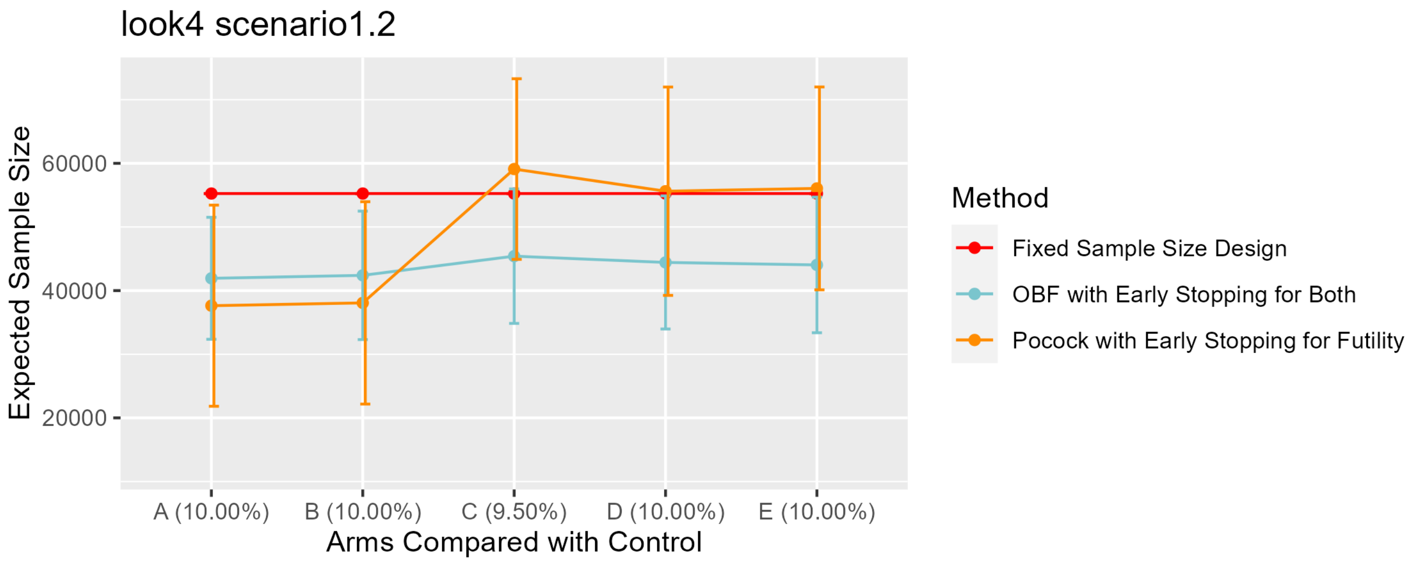
look4 scenario1.2
Expected Sample Size
60000
40000
20000
A (10.00%)
B (10.00%)
C (9.50%)
D (10.00%)
E (10.00%)
Arms Compared with Control
Method
Fixed Sample Size Design
OBF with Early Stopping for Both
Pocock with Early Stopping for Futility

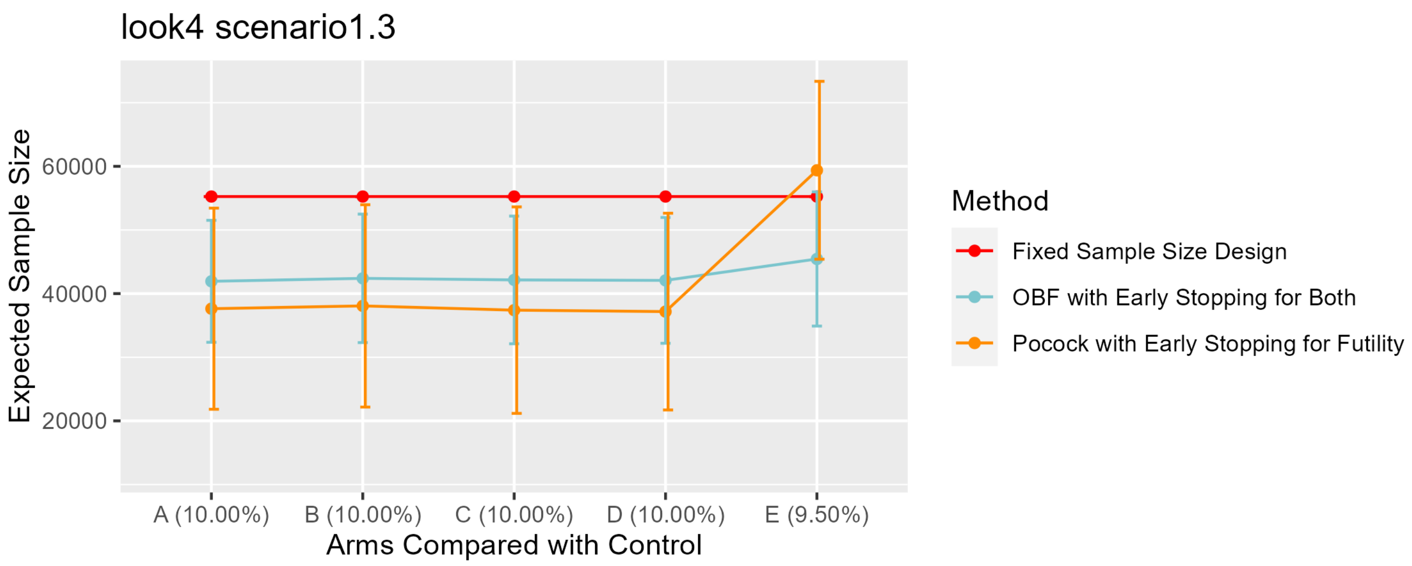

look4 scenario1.3
Expected Sample Size
60000
40000
20000
A (10.00%)
B (10.00%)
C (10.00%)
D (10.00%)
E (9.50%)
Arms Compared with Control
Method
Fixed Sample Size Design
OBF with Early Stopping for Both
Pocock with Early Stopping for Futility

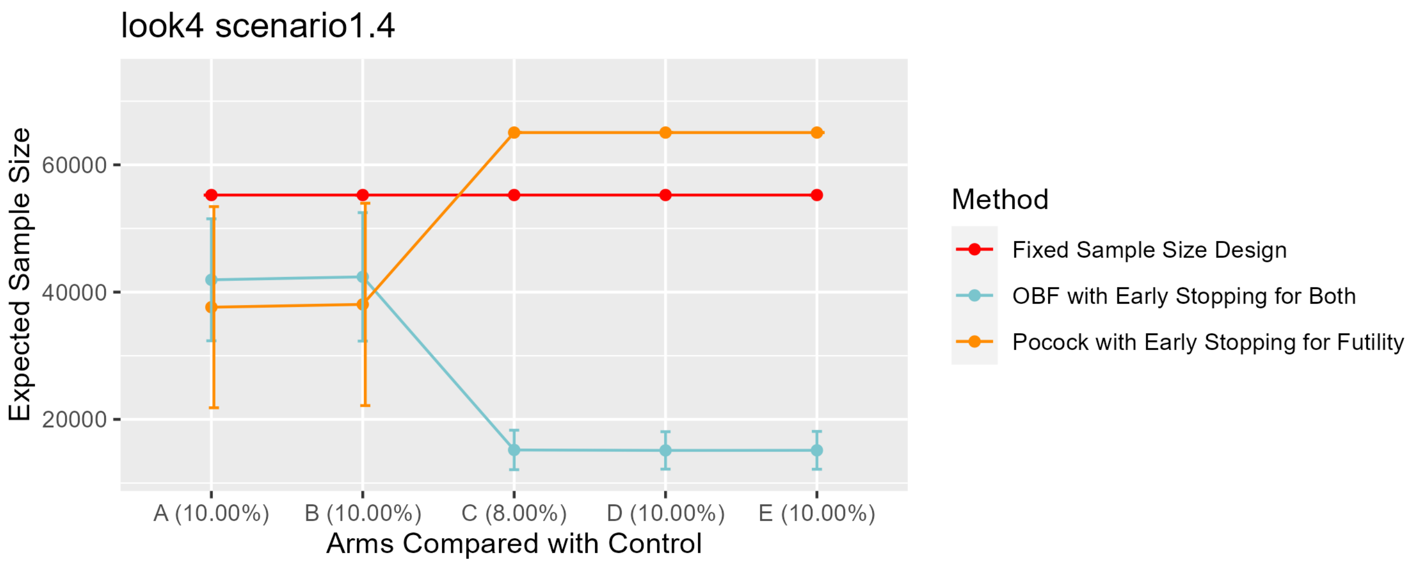

look4 scenario1.4
Expected Sample Size
60000
40000
20000
A (10.00%)
B (10.00%)
C (8.00%)
D (10.00%)
E (10.00%)
Arms Compared with Control
Method
Fixed Sample Size Design
OBF with Early Stopping for Both
Pocock with Early Stopping for Futility

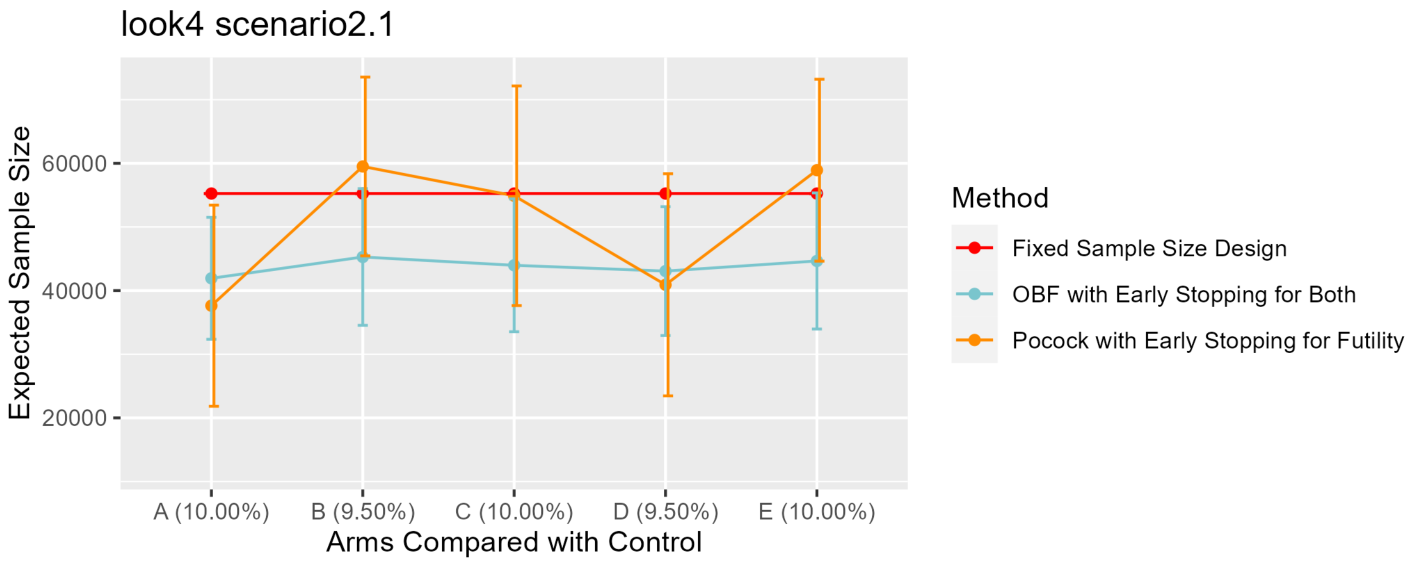

look4 scenario2.1
Expected Sample Size
60000
40000
20000
A (10.00%)
B (9.50%)
C (10.00%)
D (9.50%)
E (10.00%)
Arms Compared with Control
Method
Fixed Sample Size Design
OBF with Early Stopping for Both
Pocock with Early Stopping for Futility

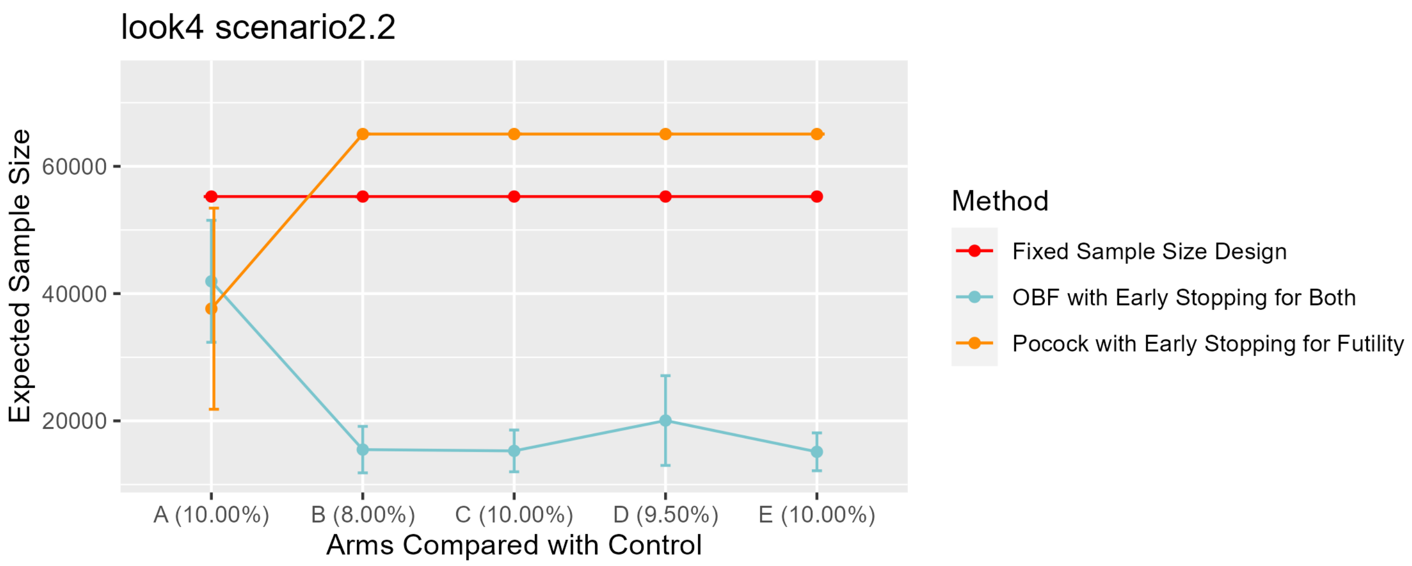
look4 scenario2.2
Expected Sample Size
60000
40000
20000
A (10.00%)
B (8.00%)
C (10.00%)
D (9.50%)
E (10.00%)
Arms Compared with Control
Method
Fixed Sample Size Design
OBF with Early Stopping for Both
Pocock with Early Stopping for Futility

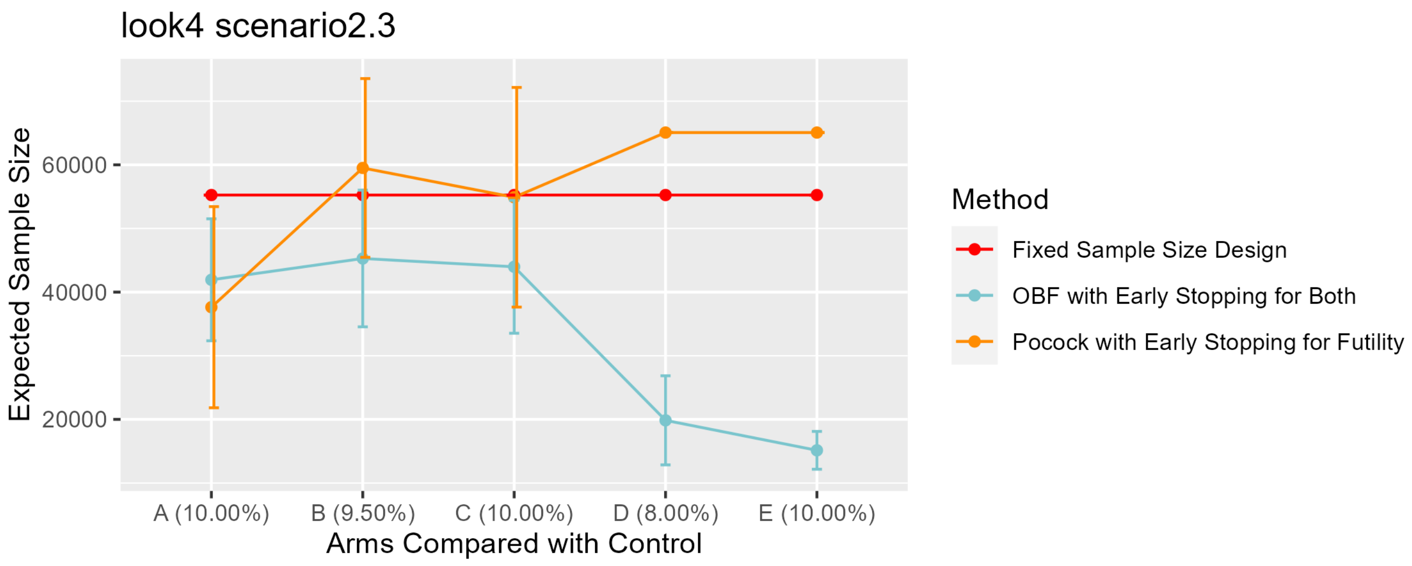
look4 scenario2.3
Expected Sample Size
60000
40000
20000
A (10.00%)
B (9.50%)
C (10.00%)
D (8.00%)
E (10.00%)
Arms Compared with Control
Method
Fixed Sample Size Design
OBF with Early Stopping for Both
Pocock with Early Stopping for Futility

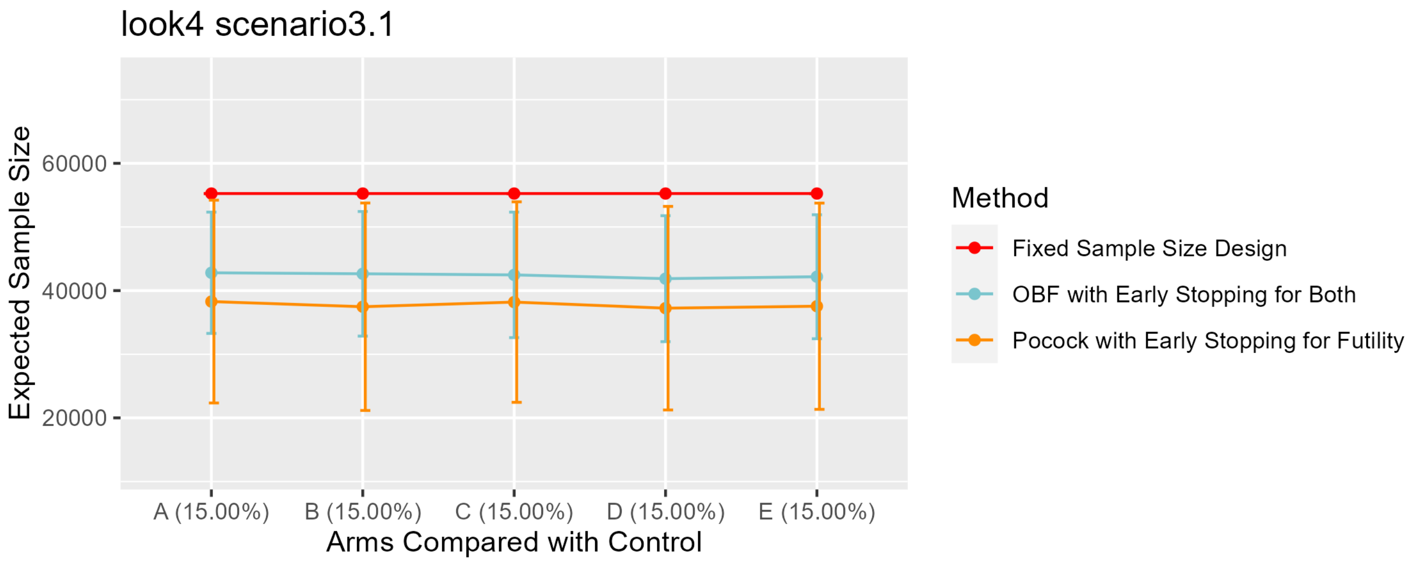
look4 scenario3.1
Expected Sample Size
60000
40000
20000
A (15.00%)
B (15.00%)
C (15.00%)
D (15.00%)
E (15.00%)
Arms Compared with Control
Method
Fixed Sample Size Design
OBF with Early Stopping for Both
Pocock with Early Stopping for Futility

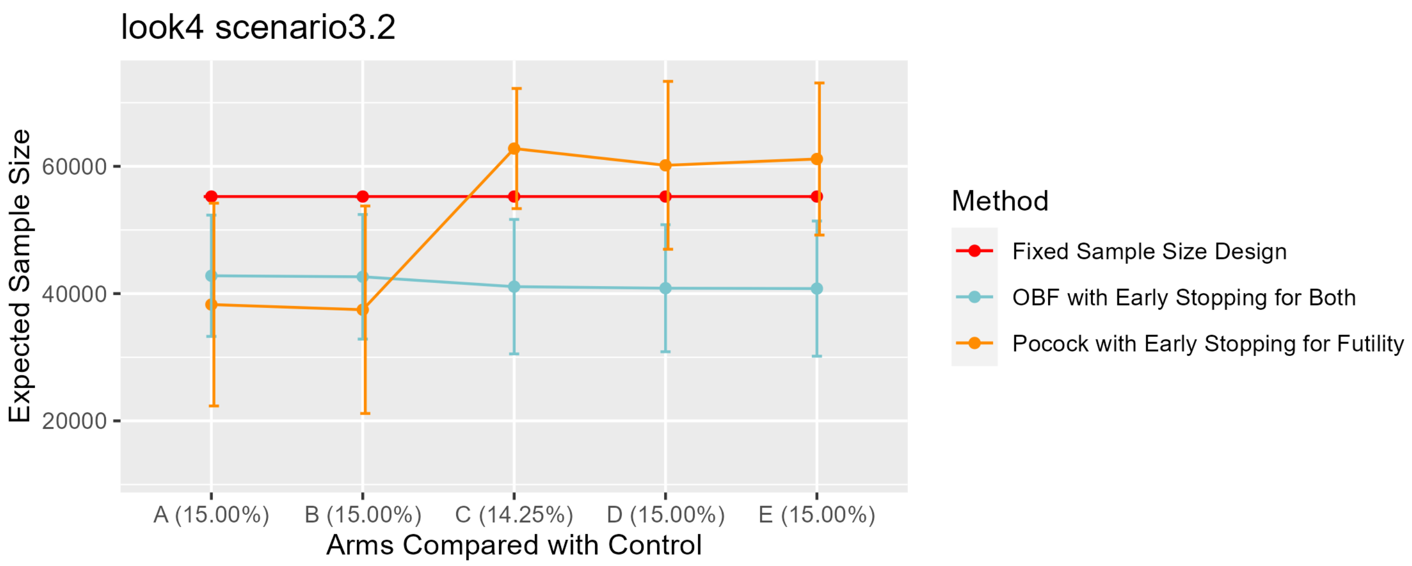
look4 scenario3.2
Expected Sample Size
60000
40000
20000
A (15.00%)
B (15.00%)
C (14.25%)
D (15.00%)
E (15.00%)
Arms Compared with Control
Method
Fixed Sample Size Design
OBF with Early Stopping for Both
Pocock with Early Stopping for Futility

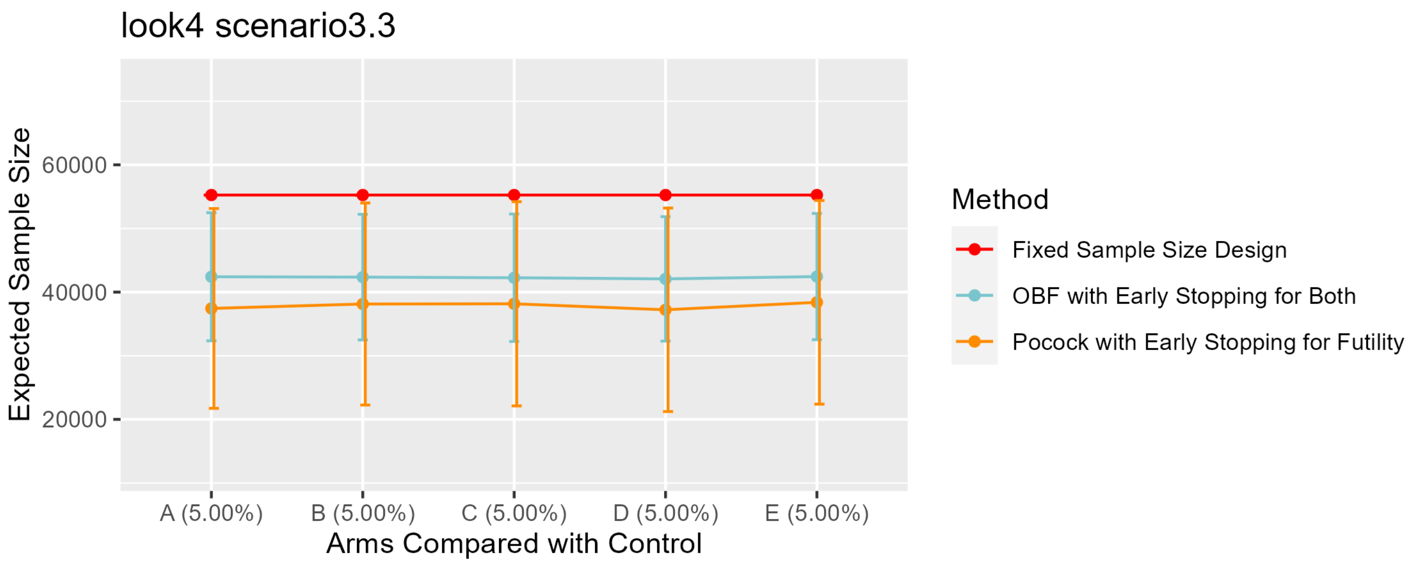
look4 scenario3.3
Expected Sample Size
60000
40000
20000
A (5.00%)
B (5.00%)
C (5.00%)
D (5.00%)
E (5.00%)
Arms Compared with Control
Method
Fixed Sample Size Design
OBF with Early Stopping for Both
Pocock with Early Stopping for Futility

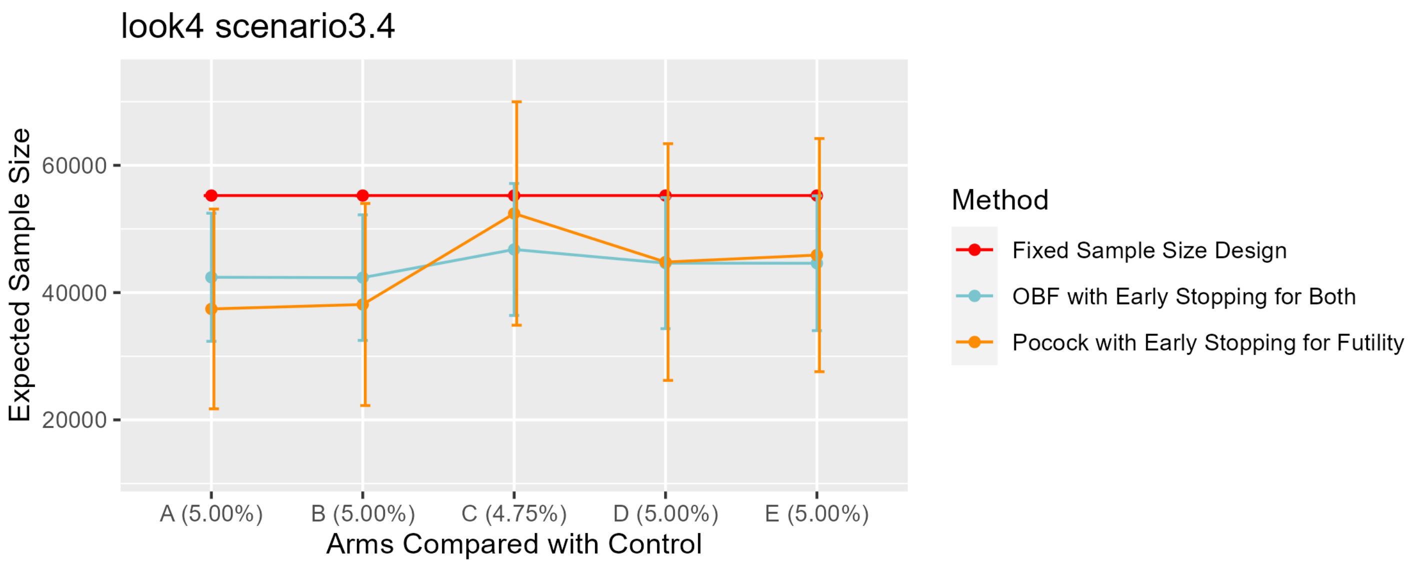
look4 scenario3.4
Expected Sample Size
60000
40000
20000
A (5.00%)
B (5.00%)
C (4.75%)
D (5.00%)
E (5.00%)
Arms Compared with Control
Method
Fixed Sample Size Design
OBF with Early Stopping for Both
Pocock with Early Stopping for Futility

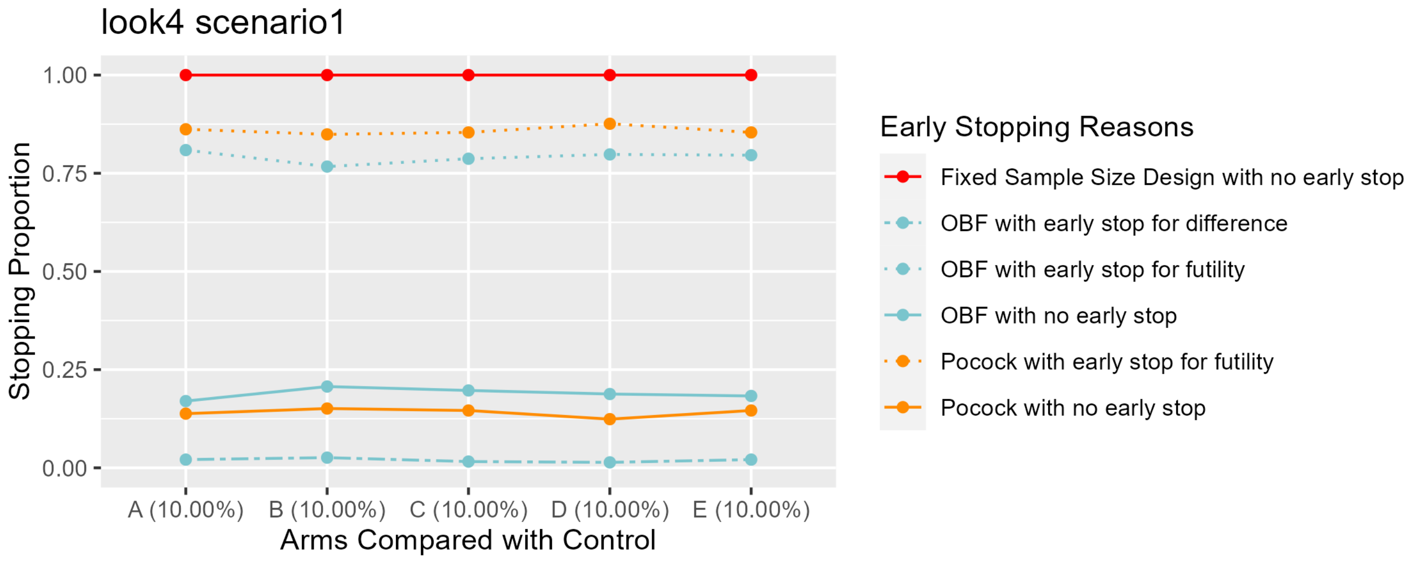
look4 scenario1
Stopping Proportion
1.00
0.75
0.50
0.25
0.00
A (10.00%)
B (10.00%)
C (10.00%)
D (10.00%)
E (10.00%)
Arms Compared with Control
Early Stopping Reasons
Fixed Sample Size Design with no early stop
OBF with early stop for difference
OBF with early stop for futility
OBF with no early stop
Pocock with early stop for futility
Pocock with no early stop

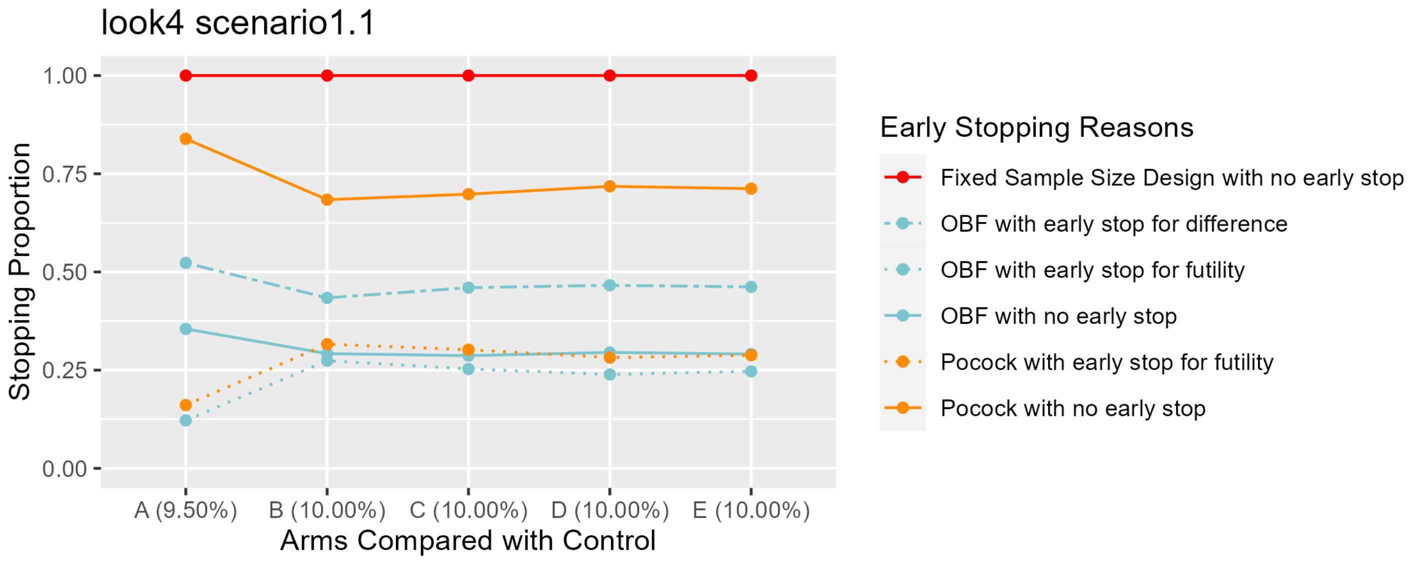
look4 scenario1.1
Stopping Proportion
1.00
0.75
0.50
0.25
0.00
A (9.50%)
B (10.00%)
C (10.00%)
D (10.00%)
E (10.00%)
Arms Compared with Control
Early Stopping Reasons
Fixed Sample Size Design with no early stop
OBF with early stop for difference
OBF with early stop for futility
OBF with no early stop
Pocock with early stop for futility
Pocock with no early stop

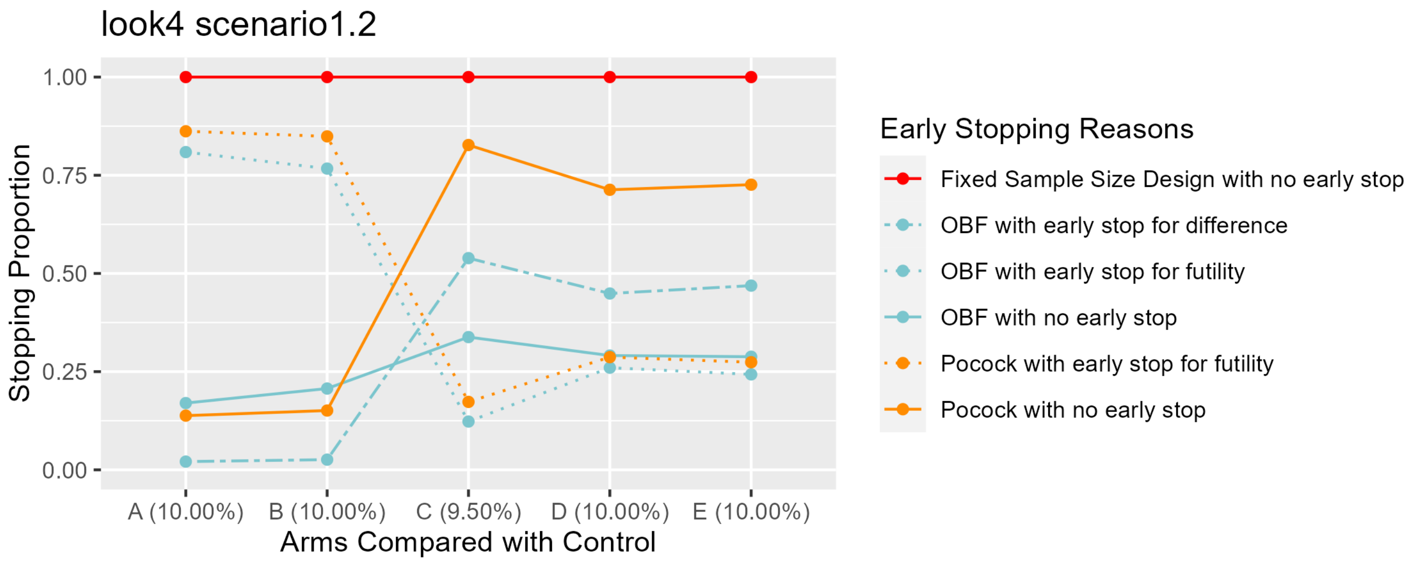
look4 scenario1.2
Stopping Proportion
1.00
0.75
0.50
0.25
0.00
A (10.00%)
B (10.00%)
C (9.50%)
D (10.00%)
E (10.00%)
Arms Compared with Control
Early Stopping Reasons
Fixed Sample Size Design with no early stop
OBF with early stop for difference
OBF with early stop for futility
OBF with no early stop
Pocock with early stop for futility
Pocock with no early stop

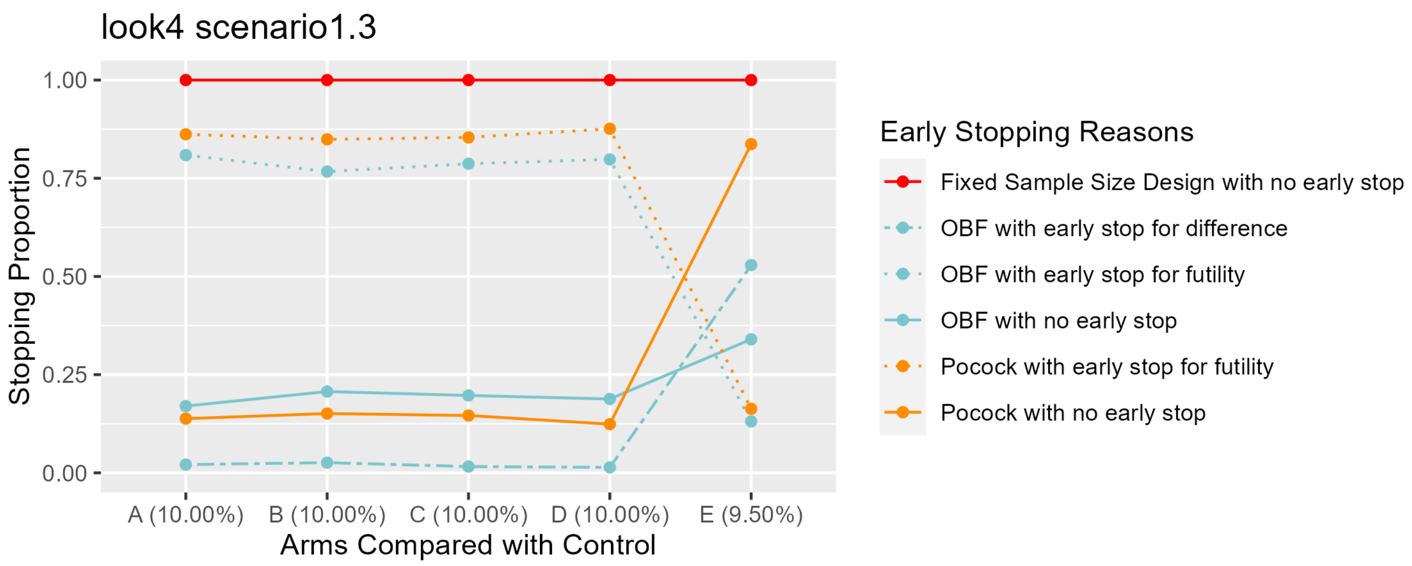
look4 scenario1.3
1.00
0.75
0.50
0.25
0.00
Stopping Proportion
A (10.00%)
B (10.00%)
C (10.00%)
D (10.00%)
E (9.50%)
Arms Compared with Control
Early Stopping Reasons
Fixed Sample Size Design with no early stop
OBF with early stop for difference
OBF with early stop for futility
OBF with no early stop
Pocock with early stop for futility
Pocock with no early stop

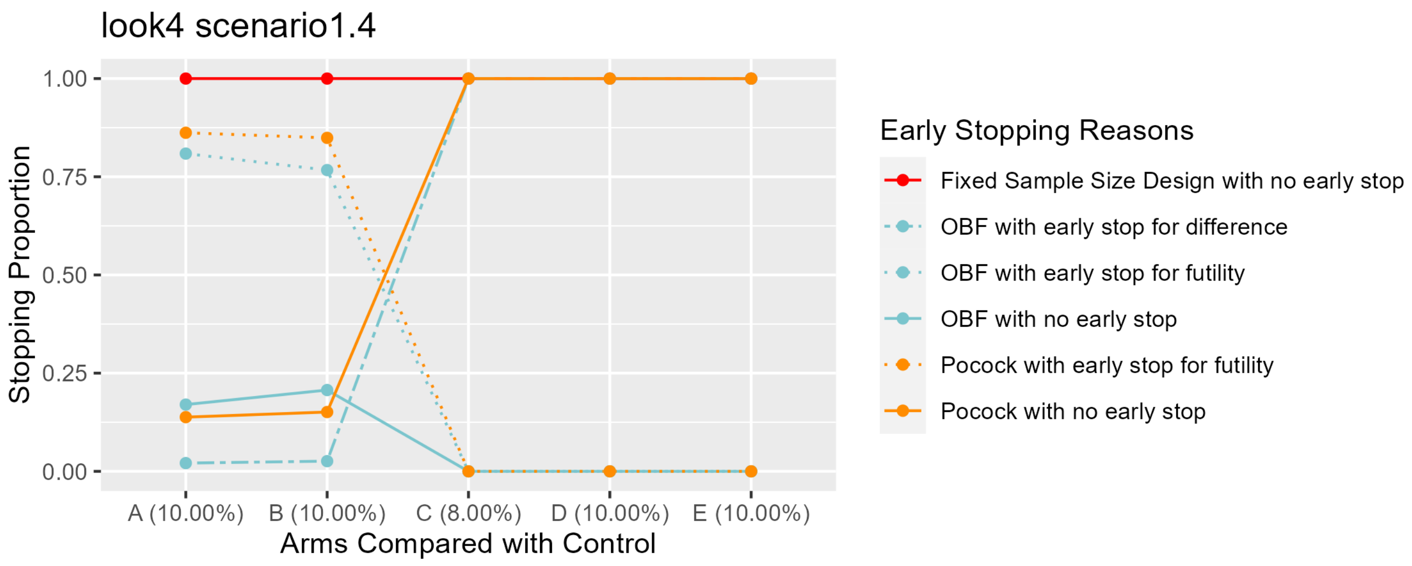
look4 scenario1.4
1.00
0.75
0.50
0.25
0.00
Stopping Proportion
A (10.00%)
B (10.00%)
C (8.00%)
D (10.00%)
E (10.00%)
Arms Compared with Control
Early Stopping Reasons
Fixed Sample Size Design with no early stop
OBF with early stop for difference
OBF with early stop for futility
OBF with no early stop
Pocock with early stop for futility
Pocock with no early stop

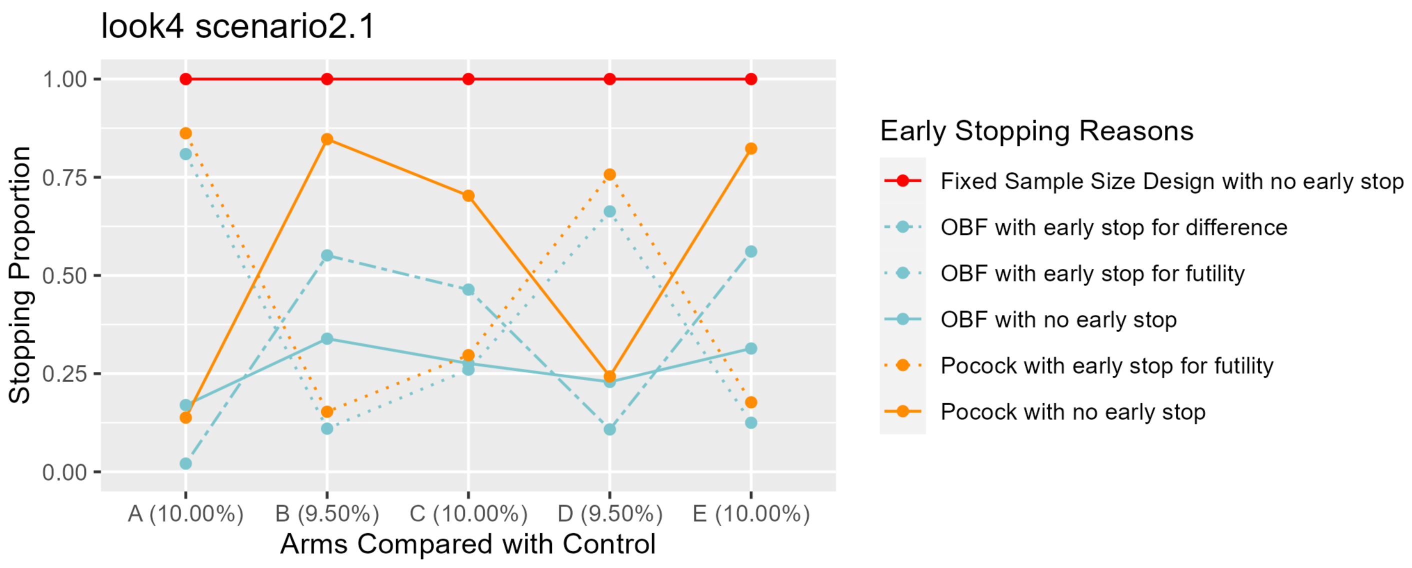
look4 scenario2.1
1.00
0.75
0.50
0.25
0.00
Stopping Proportion
A (10.00%)
B (9.50%)
C (10.00%)
D (9.50%)
E (10.00%)
Arms Compared with Control
Early Stopping Reasons
Fixed Sample Size Design with no early stop
OBF with early stop for difference
OBF with early stop for futility
OBF with no early stop
Pocock with early stop for futility
Pocock with no early stop

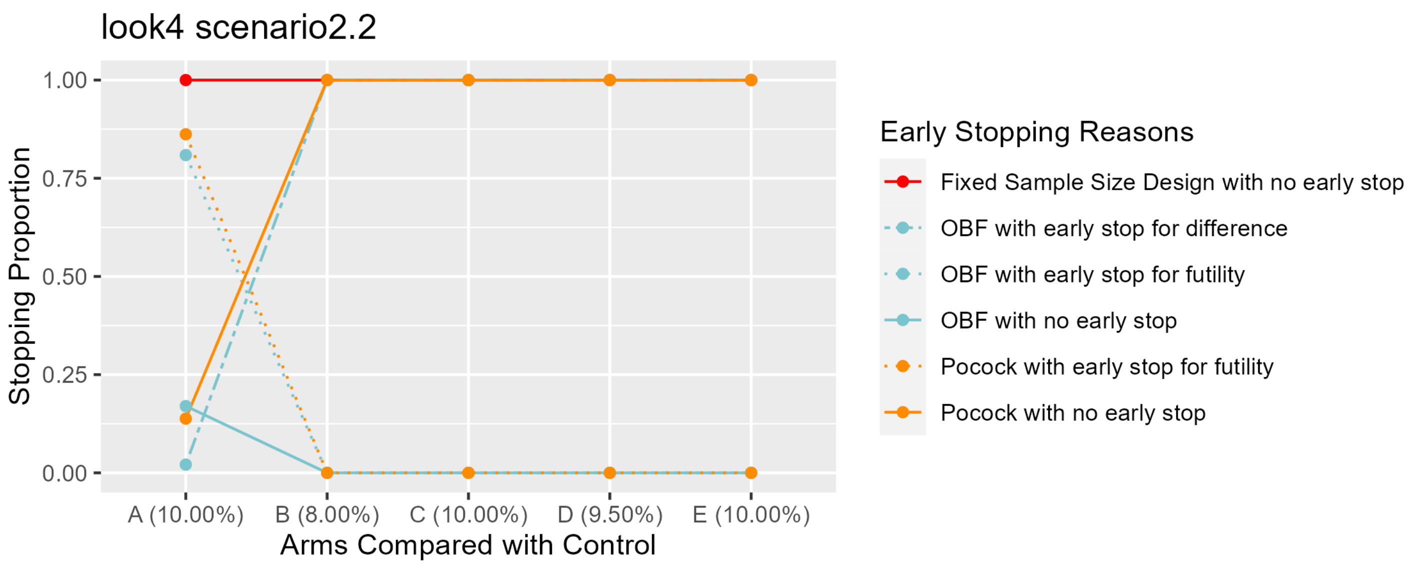
look4 scenario2.2
Stopping Proportion
1.00
0.75
0.50
0.25
0.00
A (10.00%)
B (8.00%)
C (10.00%)
D (9.50%)
E (10.00%)
Arms Compared with Control
Early Stopping Reasons
Fixed Sample Size Design with no early stop
OBF with early stop for difference
OBF with early stop for futility
OBF with no early stop
Pocock with early stop for futility
Pocock with no early stop

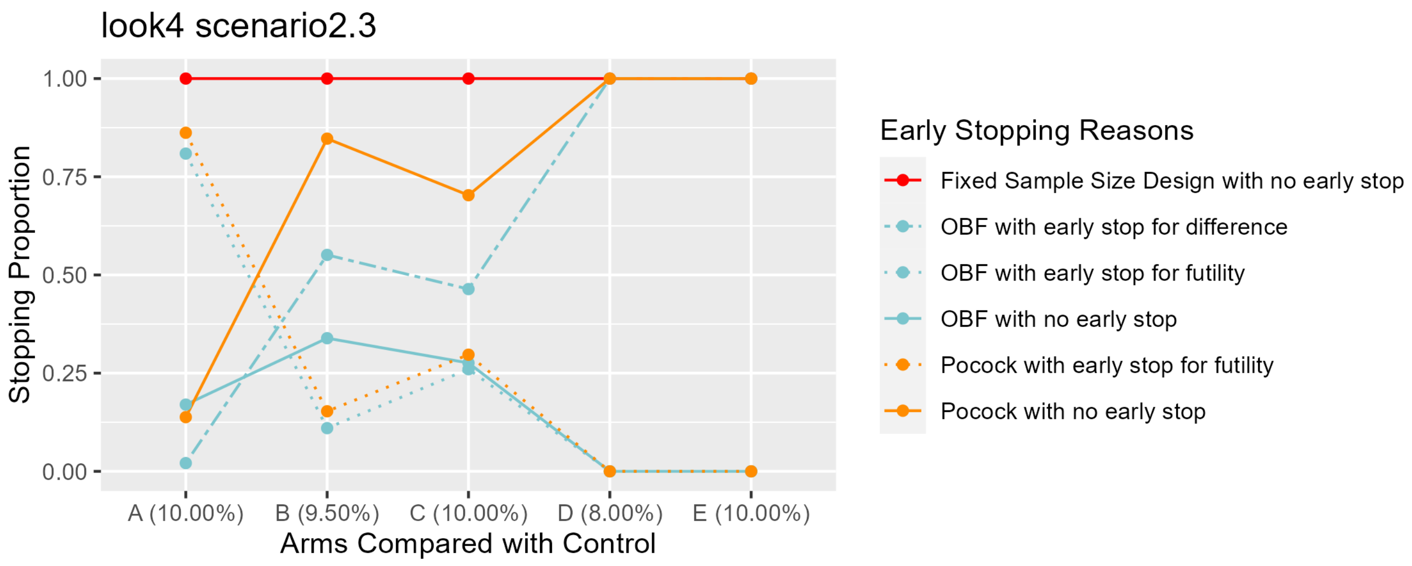
look4 scenario2.3
Stopping Proportion
1.00
0.75
0.50
0.25
0.00
A (10.00%)
B (9.50%)
C (10.00%)
D (8.00%)
E (10.00%)
Arms Compared with Control
Early Stopping Reasons
Fixed Sample Size Design with no early stop
OBF with early stop for difference
OBF with early stop for futility
OBF with no early stop
Pocock with early stop for futility
Pocock with no early stop

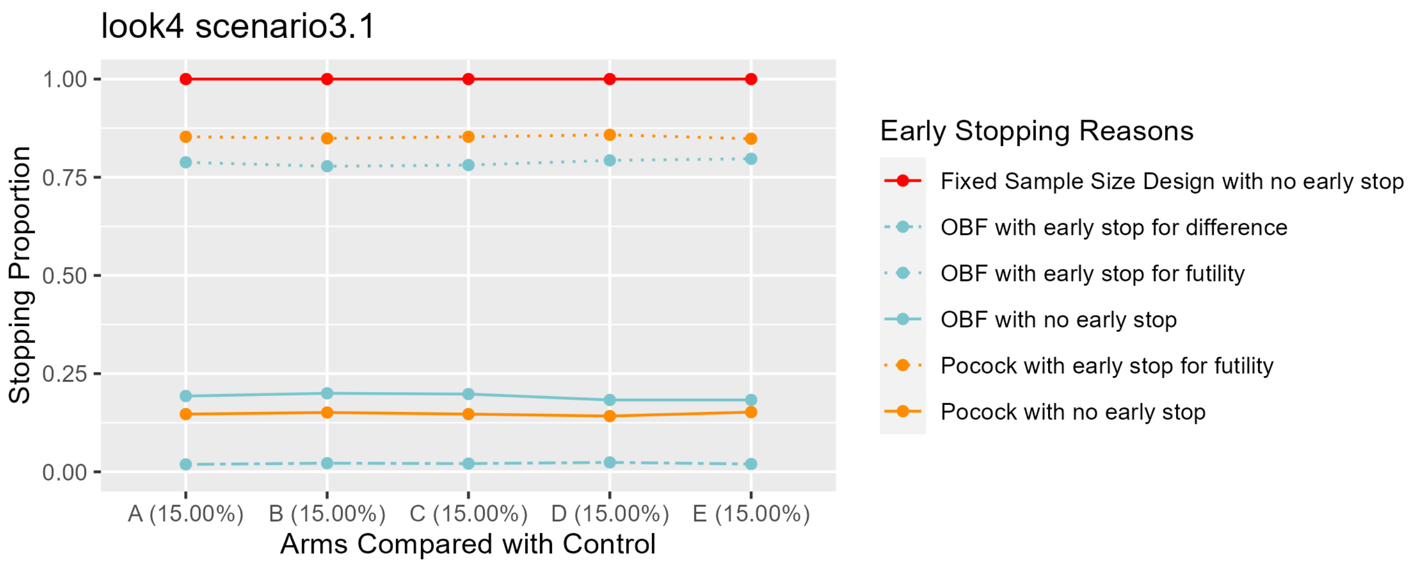
look4 scenario3.1
Stopping Proportion
1.00
0.75
0.50
0.25
0.00
A (15.00%)
B (15.00%)
C (15.00%)
D (15.00%)
E (15.00%)
Arms Compared with Control
Early Stopping Reasons
Fixed Sample Size Design with no early stop
OBF with early stop for difference
OBF with early stop for futility
OBF with no early stop
Pocock with early stop for futility
Pocock with no early stop

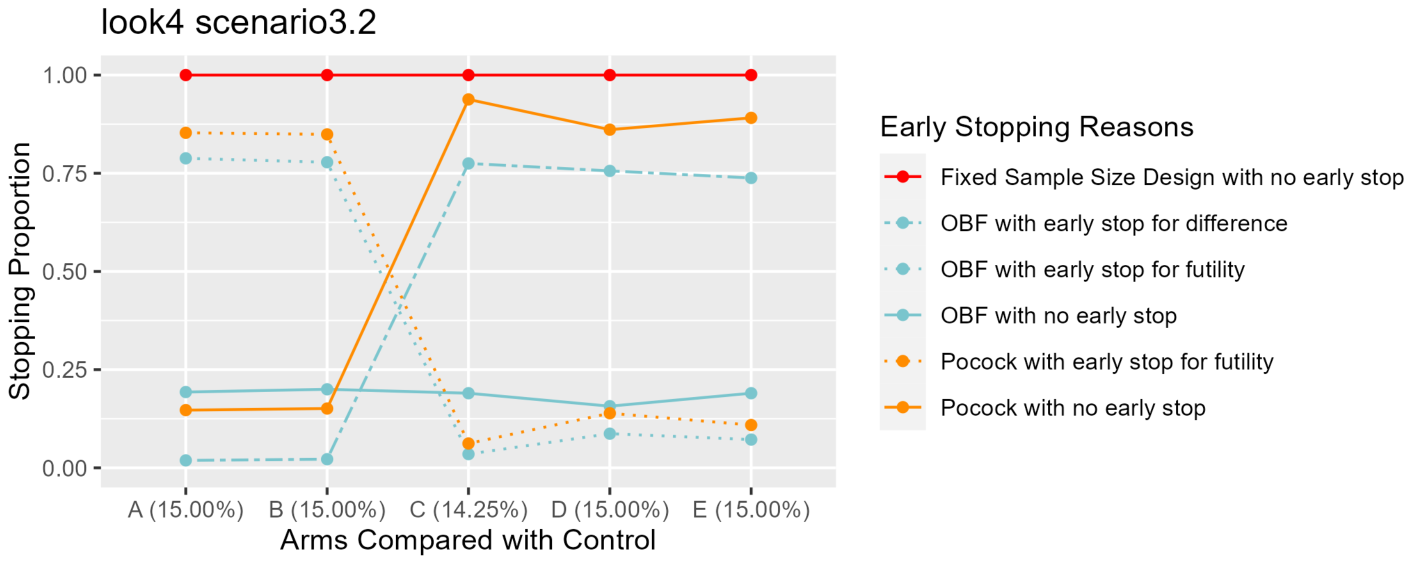
look4 scenario3.2
Stopping Proportion
1.00
0.75
0.50
0.25
0.00
A (15.00%)
B (15.00%)
C (14.25%)
D (15.00%)
E (15.00%)
Arms Compared with Control
Early Stopping Reasons
Fixed Sample Size Design with no early stop
OBF with early stop for difference
OBF with early stop for futility
OBF with no early stop
Pocock with early stop for futility
Pocock with no early stop

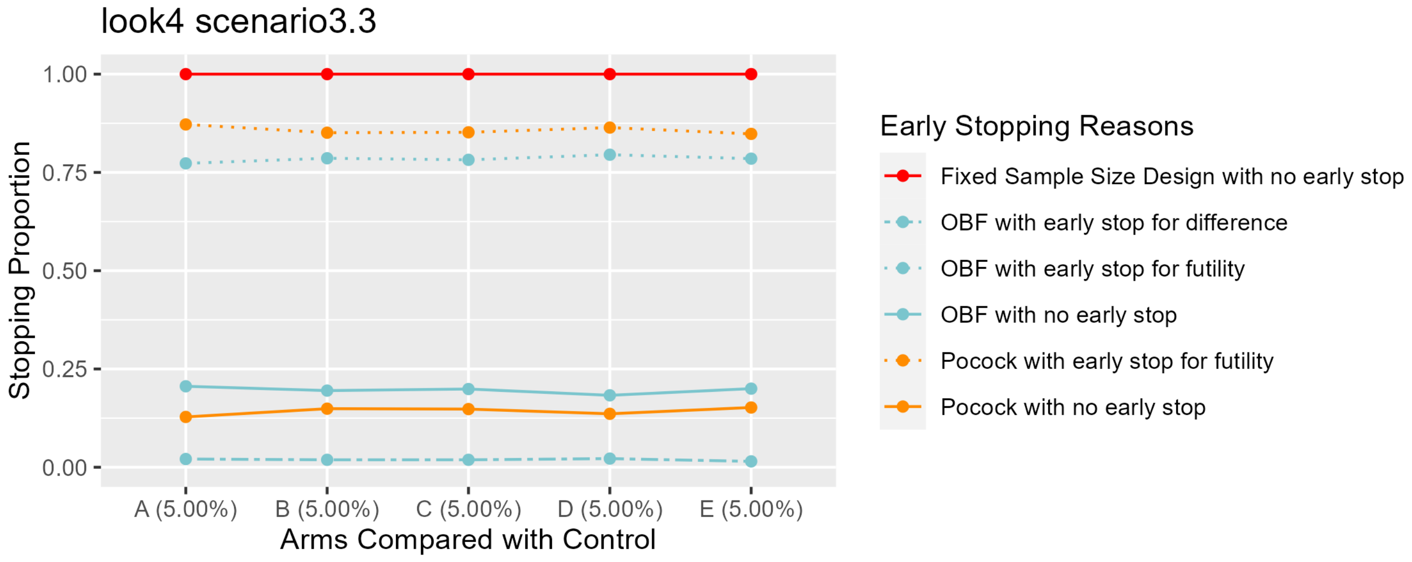
look4 scenario3.3
Stopping Proportion
1.00
0.75
0.50
0.25
0.00
A (5.00%)
B (5.00%)
C (5.00%)
D (5.00%)
E (5.00%)
Arms Compared with Control
Early Stopping Reasons
Fixed Sample Size Design with no early stop
OBF with early stop for difference
OBF with early stop for futility
OBF with no early stop
Pocock with early stop for futility
Pocock with no early stop

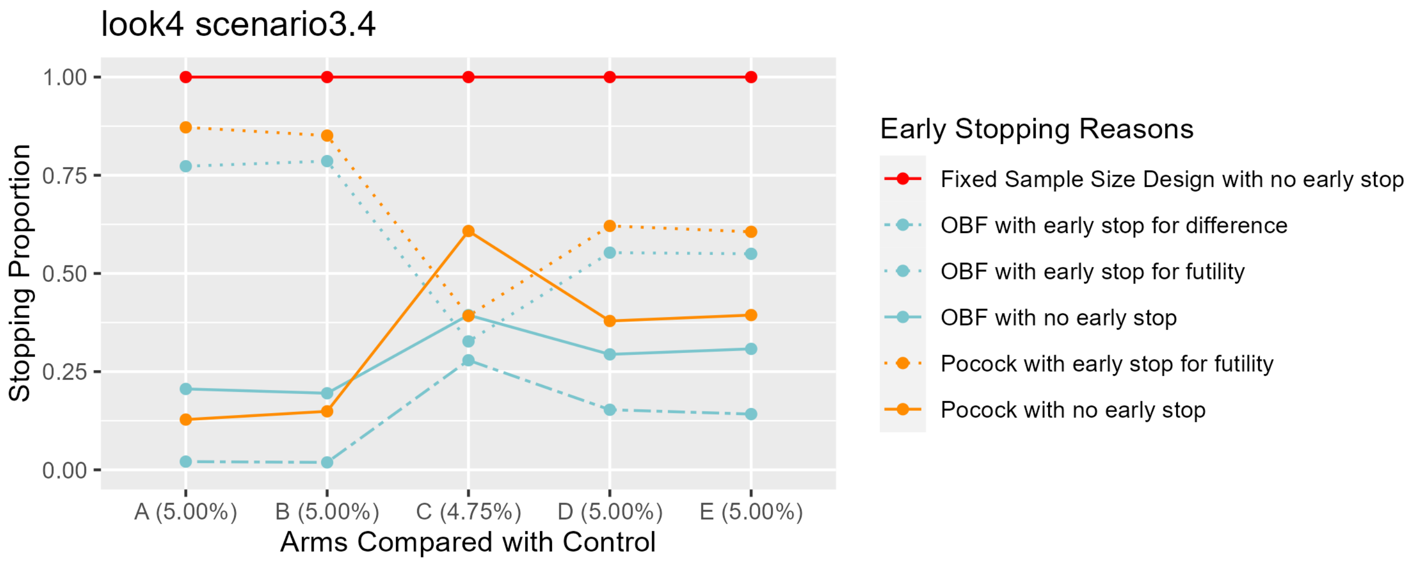
look4 scenario3.4
Stopping Proportion
1.00
0.75
0.50
0.25
0.00
A (5.00%)
B (5.00%)
C (4.75%)
D (5.00%)
E (5.00%)
Arms Compared with Control
Early Stopping Reasons
Fixed Sample Size Design with no early stop
OBF with early stop for difference
OBF with early stop for futility
OBF with no early stop
Pocock with early stop for futility
Pocock with no early stop
